# Supplementary material for: Quantifying information accumulation encoded in the dynamics of biochemical signaling
Source: Nat Commun. 2021 Feb 24;12:1272. doi: 10.1038/s41467-021-21562-0 (PMC7904837; doi:10.1038/s41467-021-21562-0)
Supplement: Supplementary file 1 — Supplementary Information [file 41467_2021_21562_MOESM1_ESM.pdf]

Supplemental Information: Quantifying information accumulation encoded in the dynamics of biochemical signaling

Ying Tang,[1,2] Adewunmi Adelaja,[1,2] Felix X.-F. Ye,[3] Eric Deeds,[1,4] Roy Wollman,[1,4,5,*] and Alexander Hoffmann[1,2,†]

[1]*Institute for Quantitative and Computational Biosciences, University of California, Los Angeles, CA 90095*
[2]*Department of Microbiology, Immunology, and Molecular Genetics, University of California, Los Angeles, CA 90095*
[3]*Department of Applied Mathematics and Statistics, Johns Hopkins University, Baltimore, MD 21218, USA*
[4]*Department of Integrative Biology and Physiology, University of California, Los Angeles, CA 90095*
[5]*Department of Chemistry and Biochemistry, University of California, Los Angeles, CA 90095*

In the Supplemental Information, we provide details on the results of the main text, in a self-consistent manner. We first formulate the dynamical mutual information in Sect. I. Sections II, III are the numerical estimation on the dynamical mutual information by the hidden Markov and the time-inhomogeneous Markov model separately. In both sections, we also list the previous formulation on the entropy for the two models. They differ from the present trajectory-wise statistics defined for each measured trajectory. For the hidden Markov model, we consider its minimal model to validate the method of calculating the dynamical mutual information. In Sect. IV, we compare the various approaches on the mutual information estimation, and demonstrate the biological insights from the present method. We end up with providing the experimental information in Sect. V.

CONTENTS

I. Supplementary Note 1: Theoretical formulation of the dynamical mutual information 2
   A. The trajectory entropy 2
   B. The dynamical mutual information and its maximum 3
   C. Remarks on the dynamical mutual information 4

II. Supplementary Note 2: Quantifying the dynamical mutual information by the hidden Markov model 5
   A. Theoretical formulations for the hidden Markov model 6
      1. The trajectory probability 6
      2. The trajectory entropy 6
   B. Numerical implementations on the dynamical mutual information 7
      1. Training the hidden Markov model by experimental data 7
      2. Quantification on the model performance 8
      3. Estimation on the dynamical mutual information 10
      4. The dependence on the number of parameters, measured cells and time points 13
   C. The other relevant statistical quantities from the hidden Markov model 16
      1. The entropy rate and normalized trajectory probability 16
      2. The model-wise statistics from the hidden Markov model 17
   D. A minimal model of the hidden Markov process 18
      1. The trajectory entropy 19
      2. The dynamical mutual information 20
      3. Numerical estimation on the dynamical mutual information 23

III. Supplementary Note 3: Quantifying the dynamical mutual information by the time-inhomogeneous Markov model 23
   A. Numerical implementations on the dynamical mutual information 23
      1. Training the time-inhomogeneous Markov model by experimental data 23

* rwollman@ucla.edu
† ahoffmann@ucla.edu

2. Quantification on the model performance 24
3. The suitability of the two models for representing various signaling dynamics 24
4. Estimation on the dynamical mutual information 25
B. The model-wise mutual information from the time-inhomogeneous Markov model 25

IV. Supplementary Note 4: Comparison with the previous methods of calculating the mutual information 26
A. The time-point method 26
B. The vector method 27
C. The decoding-based method 27
D. The present method 28
1. Biological insights from the present method 29

V. Supplementary Note 5: Experimental information 30
A. Experimental information for the NFκB dataset 30
B. Tables of the datasets 31

References 31

VI. Supplementary Figures 33

VII. Supplementary Tables 55

I. SUPPLEMENTARY NOTE 1: THEORETICAL FORMULATION OF THE DYNAMICAL MUTUAL INFORMATION

In this section, we provide the formulation on the trajectory probability, the trajectory entropy, and the dynamical mutual information (dMI) for a stochastic process. Compared with the conventional estimation on the mutual information [1], here we formulated the mutual information in a trajectory-wise manner and used the trajectory probability inferred from dynamical models, e.g., the hidden Markov model. Therefore, we termed the estimated result as the dMI in this manuscript. For clarity, we listed the mathematical symbols in Table S1.

A. The trajectory entropy

For a given stochastic dynamics, we denote a single stochastic trajectory by $y(t)$ as a function of time t. To describe a series of measurements in experiment, time is a discrete variable and the trajectory represents the time series: $y(t) = y_{1:N}$, where the subscript $1 : N$ denotes from the 1-st to the N-th measurements. In the following, we will mainly use the discrete subscript and will specify the continuous case when necessary.

Under a set of M conditions of adding various stimulus, each condition contains m_i measured trajectories of the signaling response for different cells, giving a trajectory ensemble $\{y^{i,j}_{1:N}\}_{1\leq j\leq m_i}$. The first index i ($i = 1, \ldots, M$) in superscript denotes the index of conditions. The second index j is to identify the trajectories. Each measured trajectory contains the noise due to inherent heterogeneity between individual cells and technical noise from measurements.

For each condition i, we will use stochastic models, including the hidden Markov model (this section and Sect. II) and the time-inhomogeneous Markov model (Sect. III), to learn the trajectory ensemble of the experimental data and sample trajectories as an approximation to the data. For the hidden Markov model, there is another hidden layer of states that emit the observed time series. The time series of corresponding hidden states are represented by $x(t) = x_{1:N}$, which follows a Markovian dynamics [2].

For a continuous-time stochastic process, each trajectory with probability $p[y(t)]$ has its the trajectory entropy defined as [3, 4]:

$$s[y(t)] = -\log_2 p[y(t)]. \tag{S1.1}$$

All the logarithm function in this manuscript has base 2: $\log_2$. For each trajectory measured on the discrete time, $y_{1:n}$, its the trajectory entropy is similarly defined as:

$$H(y_{1:n}) = -\log_2 p(y_{1:n}) \tag{S1.2}$$

To calculate the trajectory entropy, we thus need to estimate the trajectory probability $p(y_{1:n})$. We will provide the method for the estimation through separately using the hidden Markov model and the time-inhomogeneous Markov model in next sections.

B. The dynamical mutual information and its maximum

In this subsection, we estimate the mutual information between the condition (S) and the trajectories $\{y^{i,j}_{1:N}\}_{1\leq j\leq m_i}$ of responses (R). First, we need to estimate the trajectory entropy for the data of each condition. Following [5], we use the differential entropy as the Shannon entropy. For the set of m_i trajectories $\{y^{i,j}_{1:N}\}_{m_i}$ under the stimulus $S=i$, the conditional differential entropy is estimated with the probability distribution of observing the trajectories:

$$H(R^i_{1:n}|S=i) = -\sum_{j=1}^{m_i} \delta_{i,j} \log_2 p(R^i_{1:n} = y^{i,j}_{1:n}|S=i), \tag{S1.3}$$

where the subscript of R denotes the time points. Here, $p(R^i_{1:n} = y^{i,j}_{1:n}|S=i)$ is the trajectory probability of the j-th trajectory up to the n-th time point, under the i-th stimulus. It can be estimated by Eq. (S2.3) below. To avoid confusion, we remark that here S does not denote "sender", and $H(R^i_{1:n}|S=i)$ is the conditional trajectory entropy rather than the mutual information between "receiver" and "sender".

Here, we did not include the prefactor when calculating the trajectory entropy: we used $H(y_{1:n}) = -\log_2 p(y_{1:n})$ instead of $\hat{H}(y_{1:n}) = -p(y_{1:n})\log_2 p(y_{1:n})$. The reason is that we considered the differential entropy defined in the trajectory configuration space. Given the discrete measurements in a continuous trajectory space, the differential entropy is a proper choice [5]. It is also practically unfeasible if we used the trajectory probability as the prefactor, because the trajectory probabilities become infinitesimal when time points increase and are not normalizable, which will give huge bias on weighting the trajectory entropy.

Consequently, the prefactor $\delta_{i,j}$ in Eq. (S1.3) needs a special treatment. For each trajectory ensemble, $\delta_{i,j}$ is the prior weight of observing the trajectory $y^{i,j}$ under the stimulus i. Following the treatment in [5], we set $\delta_{i,j} = 1/m_i$ for each condition, because all m_i trajectory entropies are equally likely when no prior knowledge was applied. This treatment did the Monte-Carlo type of sampling from the trajectory probability space and calculated the mean trajectory entropy of the subsamples. Once the measured trajectories are sufficient to cover the major configurations of the full probability distribution, the average trajectory entropy approximates to the entropy of the full distribution. The treatment leads to the trajectory entropy Eq. (S1.2) averaged on all the trajectories under the stimulus. Then, the conditional trajectory entropy for the i-th stimulus is:

$$H(R^i_{1:n}|S=i) = -\sum_{j=1}^{m_i} \frac{1}{m_i} \log_2 p(R^i_{1:n} = y^{i,j}_{1:n}|S=i). \tag{S1.4}$$

Given the probability of various stimulus condition $q_i = p(S=i)$, we get the total conditional entropy by summing over the conditional entropies for all the stimuli:

$$\begin{aligned} H(R_{1:n}|S) &= \sum_{i=1}^{M} q_i H(R^i_{1:n}|S=i) \\ &= -\sum_{i=1}^{M} q_i \sum_{j=1}^{m_i} \frac{1}{m_i} \log_2 p(R^i_{1:n} = y^{i,j}_{1:n}|S=i). \end{aligned} \tag{S1.5}$$

We now move to calculate the unconditional entropy, which requires a more careful treatment. Since the trajectories collected in experiment are under specific stimulus, we do not directly have the unconditional trajectory probabilities. We borrow the approach in [5], and the unconditional trajectory probabilities are obtained by weighting the conditional trajectory probabilities with the stimulus probability distribution q_i. For each set of responses under one condition, e.g. for the trajectories of i-th stimulus $\{y^{i,j}_{1:N}\}_{1\leq j\leq m_i}$, their unconditional trajectory probabilities are approximated as:

$$p(R^i_{1:n} = y^{i,j}_{1:n}) = \sum_{k=1}^{M} q_k p(R^i_{1:n} = y^{i,j}_{1:n}|S=k). \tag{S1.6}$$

Note that we have used i, k as the index of stimulus conditions. The $p(R^i_{1:n} = y^{i,j}_{1:n}|S=k)$ is the conditional probabilities of the trajectories $\{y^{i,j}_{1:N}\}_{1\leq j\leq m_i}$ under the stimulus condition k, and can be estimated by applying the trajectories $\{y^{i,j}_{1:N}\}_{1\leq j\leq m_i}$ under the stimulus i to the model trained by the trajectory ensemble under the stimulus k.

Then, the total unconditional trajectory entropy is given by [5]:

$$\begin{aligned} H(R_{1:n}) &= -\sum_{i=1}^{M} q_i \sum_{j=1}^{m_i} \frac{1}{m_i} \log_2 p(R^i_{1:n} = y^{i,j}_{1:n}) \\ &= -\sum_{i=1}^{M} q_i \sum_{j=1}^{m_i} \frac{1}{m_i} \log_2 \Big[\sum_{k=1}^{M} q_k p(R^i_{1:n} = y^{i,j}_{1:n}|S=k)\Big]. \end{aligned} \tag{S1.7}$$

Thus, given an ensemble of trajectories under one stimulus, we only need to estimate the conditional trajectory probabilities $p(R^i_{1:n} = y^{i,j}_{1:n}|S = i)$, $p(R^i_{1:n} = y^{i,j}_{1:n}|S = k)$. They can be numerically estimated from the trained model, such as by the forward algorithm of the hidden Markov model, Eq. (S2.3). For each condition, the trajectory probability is calculated by using the transition and emission matrices of the trained hidden Markov model. For every trajectory, the forward algorithm Eq. (S2.3) constructs the trajectory probability with using the measured trajectory's value to take the emission probability at every time point. It can be recognized as a posterior probability under the hidden Markov model trained by all trajectories of one condition. We termed this method as the trajectory-wise method, because the trajectory entropy was calculated for each trajectory and the summation of the entropy was conducted on the finitely measured trajectories. Differently, in Sect. II C 2, we will also discuss the model-wise method, where the summation was on all the trajectory configurations allowed by the trained dynamical model, rather than for each trajectory.

Combining the above, we have a complete recipe to estimate the dynamical mutual information and its maximization. The dMI is:

$$\begin{aligned} I(R_{1:n};S) &= H(R_{1:n}) - H(R_{1:n}|S) \\ &= -\sum_{i=1}^{M} q_i \sum_{j=1}^{m_i} \frac{1}{m_i} \log_2 \Big[\sum_{k=1}^{M} q_k p(R^i_{1:n} = y^{i,j}_{1:n}|S=k)\Big] + \sum_{i=1}^{M} q_i \sum_{j=1}^{m_i} \frac{1}{m_i} \log_2 p(R^i_{1:n} = y^{i,j}_{1:n}|S=i). \end{aligned} \tag{S1.8}$$

It corresponds to the degree of discriminating the categorical stimuli set S.

We get the maximum dynamical mutual information with respect to the probability distribution of the stimulus conditions by

$$I_{max}(R_{1:n};S) = \max_{\mathbf{q}} I(R_{1:n};S), \tag{S1.9}$$

where $\mathbf{q} = \{q_1, q_2, \ldots, q_M\}$ with $\sum_{i=1}^{M} q_i = 1$ and $q_i \geq 0$. In numerical calculations, we conduct the optimization to get the maximum dMI $I_{max}(R_{1:n};S)$ at each time point.

The maximum dynamical mutual information quantifies the degree of distinguishable on various stimuli in a temporal manner. It increases if more diverse dynamical patterns appear, and may decrease if the dynamical patterns get similar. The maximum dynamical mutual information converges to the channel capacity when the stimulus conditions are sufficiently sampled in experiment.

In practice, one can only employ the finitely measured trajectories to acquire the knowledge on the possible trajectory configurations, and the measurements are subject to noise. Here we used the trajectory configurations generated from the dynamical models to approximate the possible configurations under each stimulus. The model was trained by all the available data and is the maximum likelihood estimate (Baum-Welch algorithm [6]) of the measured trajectory configurations. It generates the trajectory configuration space that can match with the measured trajectories with maximum likelihood. That is, the trajectory configurations generated from the model are a best possible estimation of the allowable configurations based on the available data. An unbiased estimator would then be reached when the hidden Markov model can accurately represent the measured trajectory ensemble without overfitting.

C. Remarks on the dynamical mutual information

The dMI here was for the categorical stimuli. Indeed, Eq. (S1.8) can be rewritten as:

$$\begin{aligned} I(R_{1:n};S) &= -\sum_{i=1}^{M} q_i \sum_{j=1}^{m_i} \frac{1}{m_i} \log_2 \Big[\sum_{k=1}^{M} q_k w_k(j)\Big] + \sum_{i=1}^{M} q_i \sum_{j=1}^{m_i} \frac{1}{m_i} \log_2 [w_i(j)] \\ &= -\sum_{j=1}^{m_i} \frac{1}{m_i} \sum_{i=1}^{M} q_i \log_2 \Big[\sum_{k=1}^{M} q_k \frac{w_k(j)}{w_i(j)}\Big], \end{aligned} \tag{S1.10}$$

where $w_k(j) \doteq p(R^i_{1:n} = y^{i,j}_{1:n}|S = k)$ denotes the weight on the distribution of stimuli q_k. If we further recognize the term inside the log function $Q_i(j) \doteq [\sum_{k=1}^M q_k w_k(j)/w_i(j)]/\mathcal{Z}$ as a distribution $Q_i(j)$ with the normalization factor $\mathcal{Z}$, then $I(R_{1:n}; S)$ is rewritten as the cross entropy between the distributions q_i and $Q_i(j)$, $H[q, Q] \doteq -\sum_{i=1}^M q_i \log_2[Q_i(j)]$, averaged over trajectories. The $Q_i(j)$ is the reweighted distribution on q_k, and the cross entropy is between the uniform distribution of the stimuli and their reweighted distribution.

In $Q_i(j)$, the weights on q_k are determined by the similarity between the trajectory ensembles of the stimuli. The trajectory probability calculated here provides the weight with considering the dynamical patterns of trajectory ensembles. The trajectory's density estimator in [5] gives another way of weighting the stimulus distribution. In both cases, the maximization $\max_{\mathbf{q}} I(R_{1:n}; S)$ leads to the maximum cross entropy, which measures the extent of discriminating the categorical stimuli [5].

Since trajectories are rarely sampled in the full trajectory space and their probabilities becomes infinitely small as time points increase. We then followed the method in [5] to formulate the quantity $I(R_{1:n}; S)$, which quantifies the discrimination on the stimuli as the standard mutual information did [7, 8]. We thus kept using the basic terminology of "mutual information", and further emphasized the inference from dynamical models by using "dynamical mutual information".

Next, we provide the theoretical upper and lower bound on the dynamical mutual information, which also indicates that it can represent the maximum distinguishability on the stimuli. For the upper bound, we can rewrite Eq. (S1.8) into the following form:

$$\begin{aligned} I(R_{1:n}; S) &= \sum_{j=1}^{m_i} \frac{1}{m_i}\Big\{ -\sum_{i=1}^{M} q_i \log_2 \Big[q_i + \sum_{k\neq i} q_k [p(R^i_{1:n} = y^{i,j}_{1:n}|S = k)/p(R^i_{1:n} = y^{i,j}_{1:n}|S = i)]\Big]\Big\} \\ &\leq \sum_{j=1}^{m_i} \frac{1}{m_i}\Big\{ -\sum_{i=1}^{M} q_i \log_2 q_i \Big\}, \end{aligned} \tag{S1.11}$$

because q_k are all non-negative. In addition, due to the consistency and efficiency properties of maximum likelihood estimation [9], we have the following property as $n \to +\infty$,

$$\limsup_{n\to+\infty} \frac{p(R^i_{1:n} = y^{i,j}_{1:n}|S = k)}{p(R^i_{1:n} = y^{i,j}_{1:n}|S = i)} = 0, \quad \text{when } k \neq i. \tag{S1.12}$$

Then, the long-time dynamical mutual information has the limit superior in Eq. (S1.11). Its maximum with respect to the distribution q_i gives the upper bound of the maximum dynamical mutual information under the long-time limit, $\log_2 M$. To reach this upper bound requires all the stimulus conditions to be distinct such that the limit value in Eq. (S1.12) can be reached. The analysis also implies that the long-time mutual information when using the trajectory entropy $\tilde{H}$ in Eq. (S2.8) with the prefactor $1/n$ is zero.

To show the non-negativity of the mutual information in Eq. (S1.8), we first employ the property that $p(R^i_{1:n} = y^{i,j}_{1:n}|S = k) \leq p(R^i_{1:n} = y^{i,j}_{1:n}|S = i)]$ for each trajectory and each k, because the trajectory probability based on the model of the original condition should be higher than that of the other conditions. Then, Eq. (S1.8) becomes

$$\begin{aligned} I(R_{1:n}; S) &= \sum_{j=1}^{m_i} \frac{1}{m_i}\Big\{ -\sum_{i=1}^{M} q_i \log_2 \Big[\sum_{k=1}^{M} q_k [p(R^i_{1:n} = y^{i,j}_{1:n}|S = k)/p(R^i_{1:n} = y^{i,j}_{1:n}|S = i)]\Big]\Big\} \\ &\geq \sum_{j=1}^{m_i} \frac{1}{m_i}\Big\{ -\sum_{i=1}^{M} q_i \log_2 \Big[\sum_{k=1}^{M} q_k \Big]\Big\} \\ &= 0, \end{aligned} \tag{S1.13}$$

where we have used the probability normalization condition and monotonic property of logarithm function.

II. SUPPLEMENTARY NOTE 2: QUANTIFYING THE DYNAMICAL MUTUAL INFORMATION BY THE HIDDEN MARKOV MODEL

In this section, we provide detailed methods on quantifying the dynamical mutual information from the hidden Markov model. The hidden Markov model has a wide range of applications, such as speech recognition [10], communications [11], and chromatin states' inference [12]. To our best knowledge, it has not been applied to model the time series of single-cell biochemical signaling responses.

A. Theoretical formulations for the hidden Markov model

1. The trajectory probability

For a given observed trajectory $y_{1:N}$, we use the forward algorithm for the hidden Markov model to compute the joint trajectory probability ($1 \le n \le N$) [1, 13]:

$$p(y_{1:n}, x_n) = \sum_{x_{n-1}} E(y_n|x_n)T(x_n|x_{n-1})p(x_{n-1}, y_{1:n-1}), \tag{S2.1}$$

where E is the emission matrix, and T is the transition matrix for the hidden states. The two matrices are inferred from the model training by the trajectory ensemble for each condition. We consider that there is B emission states and K hidden states.

We can rewrite the forward algorithm into a matrix form:

$$\mathbf{p}_n \doteq p(y_{1:n}, x_n) = \mathbf{p}_0 TD(y_1) \cdots TD(y_n), \tag{S2.2}$$

where $\mathbf{p}_n = p(y_{1:n}, x_n)$ with the k-th component $p(x_n = k, y_{1:n})$. Besides, $\mathbf{p}_0$ is the initial hidden state distribution, which needs to be assumed when the data only have the observed time series. The matrix $D(y_n)$ is diagonal with the (k, k)-th entry as $D_{kk}(y_n) = E(y_n|x_n = k)$, which are random matrices sampled from the emission matrix independently at each step, under the given observed time series. The forward probability is conducted for each trajectory, and the observed time series specify the elements of emission matrix taken at each time step.

The trajectory probability for an observed time series is given by summing over the hidden state:

$$p(y_{1:n}) = \sum_{k=1}^{K} p(y_{1:n}, x_n = k) = \sum_{k=1}^{K} \mathbf{p}_0 TD(y_1) \cdots TD(y_n). \tag{S2.3}$$

This trajectory probability is not conserved and converges to zero exponentially with the increasing number of time points. The decay rate mainly increases with the number of emission states. The usage of more states causes a finer classification on the trajectory ensemble, making the trajectory probability of each observed time series becomes smaller (cf. the discussion in Rescaled likelihood of Sect. II B 2). Thus, it may lead to a numerical problem of infinitesimal trajectory probabilities after several time steps. To circumvent this problem, one normalization method is to consider the filtered trajectory probability: $\hat{p}(x_n|y_{1:n}) \doteq p(y_{1:n}, x_n)/p(y_{1:n})$, which gives a probability for the hidden states. At each time step, it is a normalized probability for the hidden states under the observed trajectory (cf. Sect. II C 1).

In the next subsection, we will use the trajectory probability Eq. (S2.3) instead of the normalized one, as we need to estimate the probability for each observed trajectory instead of hidden states. We will deal with the data with around 150 time points. Though the trajectory probability decays exponentially with the number of time points, this finite amount of time points has not reached the long-time limit with the trajectory probability being approximately zero.

2. The trajectory entropy

The trajectory entropy can be calculated by Eq. (S1.2), with using the trajectory probability Eq. (S2.3). We emphasize that this trajectory entropy for the hidden Markov model does not have the prefactor $1/n$ for $\log_2 p(y_{1:n})$. The entropy rate with the prefactor $\tilde{H}(y_{1:n}) = -(1/n)\log_2 p(y_{1:n})$, i.e. Eq. (S2.8) below, was mainly adopted for the hidden Markov model in literature [13–15], as compatible to the first Lyapunov exponent [16]. The prefactor $1/n$ was added to compensate the decay of trajectory probability: $p(y_{1:n})$ decays with rate B^n, where B is the number of emission states. With the prefactor, the trajectory entropy formula can have a converged order of magnitude: $\tilde{H}(y_{1:n}) \sim -(1/n)\log_2 B^{-n} = \log_2 B$, which is another reason for its popularity in literature.

However, the presence of the prefactor alters the calculation on the mutual information. We will provide an example on the mutual information estimation when using the entropy formula with the prefactor $1/n$. As exemplified in Sect. II D 2 below, we will demonstrate in a minimal model the difference between the trajectory entropy with and without the prefactor $1/n$, where the one without the prefactor leads to the correct dMI. Thus, the formula with the prefactor $1/n$ is not suitable to estimate the mutual information. Consequently, we keep using the original definition on the trajectory entropy without the prefactor, Eq. (S1.2) in [3, 4]. In addition, we will provide an alternative method to rescue the issue of probability decay in Rescaled likelihood of Sect. II B 2. This method does not affect the estimation on the mutual information, as the mutual information is the difference between the conditional and unconditional entropies, which supports the drop of the prefactor $1/n$ in the trajectory entropy formula.

B. Numerical implementations on the dynamical mutual information

In this subsection, we provide the numerical implementations to train the hidden Markov model from data, quantify the training performance, and use the model to calculate the dMI.

In short, the numerical procedures include the model training, the evaluation on the training performance, the estimation on the conditional and unconditional trajectory probabilities from the hidden Markov model, model selection and the calculation on the dMI. In Fig. S9, we illustrated the whole numerical procedures, which also contains the usage of the Markov model introduced in Sect. III.

1. Training the hidden Markov model by experimental data

First, we trained the hidden Markov model from the experimental data, which is a trajectory ensemble of single-cell signaling responses for each condition. The training output includes the emission matrix and transition matrix. One hidden Markov model was trained through the whole set of single-cell signaling trajectories under one condition. The detailed procedures are listed below.

1. Pre-treatment on the data.

   For all the trajectories under various stimuli, we chose an upper cutoff 10 and set the values over 10 to be 10, such that the range of the values are fixed for all the conditions. We have plotted the histogram of the values for all the trajectories of each stimulus' condition in Fig. S1. It shows that very few data points have values higher than 10. Therefore, the upper cutoff does not dramatically affect the following analysis. The advantage for a fixed range of response $[0, 10]$ is that the emission states are binned in a same manner for all the conditions.

2. Model training.

   We used the standard algorithm on training a hidden Markov model from the data of time series. Specifically, we chose the Baum-Welch algorithm [6], which derives a maximum likelihood estimation on the parameters of the hidden Markov model given the input trajectory ensemble. There are alternative methods such as the Viterbi algorithm [17], which is a dynamic programming to find the most likely trajectory of hidden states that generates the input data. For our dataset, the Baum-Welch algorithm performs better than the Viterbi algorithm.

   We implemented the Baum-Welch algorithm by using a developed package in MATLAB. The function *hmmtrain* trains a hidden Markov model given a set of time series as input. For the training procedure, we have generated random matrices with column's sum 1, as the initial transition and emission matrices. We have tested different choices on these two initial matrices, and found that they do not alter the performance of model training. We have increased the maximum number of iterations in the default options on training the hidden Markov model, and found it does not significantly improve the training performance. It demonstrates that the default option of the algorithm is already near to the optimal. Improvement of the training performance may be achieved by having more times series as input data.

   Besides, the function *hmmdecode* returned a trajectory probability similar to the last-step trajectory probability from our estimation from Eq. (S2.3). Since we need the full time course of the trajectory probability for each trajectory, we chose to use the calculation by Eq. (S2.3). The function *hmmviterbi* can generate the most probable path of the hidden states, however, we did not have the data of the most probable path along the hidden states and thus did not use this function. All the simulations were done by MATLAB version R2018b.

3. Sampling trajectories from the hidden Markov model.

   We used the function *hmmgenerate* to sample multiple trajectories from the trained hidden Markov model, and compared it with the input data. We repeated this process for each condition, and plotted the trajectories as heatmap separately for the data and simulation. To have a better visualization on the heatmap, we smoothed both data and the sampled trajectories by applying the MABLAB function *smooth* with "loess" option and smooth parameter 0.07. We have also tested "rloess" option, but found that it generates less similar trajectories as data when compared with "loess". The plotting of the heatmap employed the MATLAB toolboxes at GitHub (https://github.com/Adewunmi91/MACKtrack).

   When generating the heatmap of trajectories, the range of response was taken as the smallest integer larger than all the response values in each stimulus' condition separately. For better visualization, we have used an enhanced sampling method to generate the heatmap from the hidden Markov model. In detail, we sampled two times number of the trajectories than that of the data. We then picked equal number of trajectories as that of

the data with relatively smaller distance to the trajectory ensemble of the data. This enhanced sampling was only used here for the better visualization on heatmap, and was not adopted to affect other analysis.

2. Quantification on the model performance

In this subsection, we provide three measures on quantifying the performance of model training: relative Kullback-Leibler (KL) divergence; false k-nearest neighbor probability; the rescaled likelihood. We then evaluate the training performance versus the number of states. We finally discuss the less well-trained conditions.

1. Relative Kullback-Leibler (KL) divergence.

   We estimated the KL-divergence between the sampled trajectories and the data. At each time point, the values of the trajectories sampled from the model and those from the data form two distributions, $p(y_n)$ and $q(y_n)$ separately. The KL-divergence between the two distributions is given by [18]:

   $$D_{KL}[p(y_n)||q(y_n)] = \sum_{y_n} p(y_n) \log_2 \left[\frac{p(y_n)}{q(y_n)}\right]. \tag{S2.4}$$

   The KL-divergence reflects the inefficiency caused by the approximation from the model. It is a measure of entropy increase by using of the approximated distribution rather than the distribution of the data. To better evaluate the performance by KL-divergence, we further calculated the entropy of the data distribution at each time point:

   $$S[q(y_n)] = \sum_{y_n} q(y_n) \log_2 q(y_n). \tag{S2.5}$$

   Then, we got the ratio between the KL-divergence and the entropy of the data:

   $$D_{KL}[p(y_n)||q(y_n)]/S[q(y_n)], \tag{S2.6}$$

   which is named as the relative KL-divergence. In Fig. S3, we plotted the time course of the three quantities: the KL-divergence, the entropy of the data, and the relative KL-divergence for one condition.

   For each model training with a fixed number of hidden and emission states, we calculated the ratio between the KL-divergence and the entropy of the data at each time point, and took the mean relative KL-divergence along the full time course. We plotted this mean relative KL-divergence for various conditions, and showed their dependence on the number of states in Fig. S4. The relative KL-divergence becomes smaller with the increasing number of states, indicating a better approximation by the hidden Markov model to reproduce the data.

2. False k-nearest neighbor probability.

   The false k-nearest neighbor probability is defined as the probability of the false k-nearest neighbor for the trajectories from the data to sample, or vice versus. Based on the hidden Markov model, we sampled the same number of trajectories as that of the data, and compare these two trajectory ensembles. When half of the neighbors come from the "false" trajectory ensemble, the similarity between the trajectory ensembles from the model and the data reaches maximum. Thus, the false k-nearest neighbor probability 0.5 corresponds to an optimal mixing of the sampled trajectories and data, indicating that the hidden Markov model can represent the data well.

   We first estimated the false k-nearest neighbor probability for a fixed number of neighbor k. For each trajectory in the sample from the hidden Markov model, we searched its k-nearest neighbors and count the number of neighbors from the data and sample separately. The number of neighbor from the data is the "false" nearest neighbor for the chosen trajectory from the model. We did the same procedure for each trajectory from the data, and counted its false k-nearest neighbor from the sampled trajectories. We then plotted the histogram for the number of false k-nearest neighbor from the data to sample and from the sample to the data separately in Fig. S3. We fitted a binomial distribution to the histogram, and got the parameter p of the binomial distribution as the false k-nearest neighbor probability. We remark that the binomial distribution is not always perfect to fit with the false k-nearest neighbor distribution, indicating that the data and sampled trajectories are not fully mixed with each other under certain stimulus.

We calculated the false k-nearest neighbor probability with using different parameter k: $k = 10, 20, 30, 40, 50, 60, 100$. As k increases, the false k-nearest neighbor probability increases to the ideal value 0.5. However, when k becomes too large, the radius of searching nearest neighbor may cover a whole cloud of sampling set that is not well mixed with data, which causes false-positive to identify the well-mixed neighbors. Thus, there should be an upper bound on k corresponding to the radius of the nearest neighbor, and the proper choice of k depends on the number of trajectories in the dataset. As for each condition in the current dataset, the number of trajectories is typically around 500, and the parameter k in the range of less than 100 should be proper. Here, we chose $k = 60$, and took the average of the false k-nearest neighbor probabilities from the data to the sample and from the sample to the data.

In Fig. S4, when the number of hidden states is around 60 and the number of emission states is around 30, the false k-nearest neighbor probability starts to saturate versus the number of states. The saturated probability of all conditions has a mean around 0.4, indicating that the hidden Markov model can generate trajectories similar to the data with true-positive rate 80%.

We remark that the false k-nearest neighbor probability from data to sample is lower than that from sample to data (Fig. S3). It indicates that the sampled trajectories are more concentrated and less diversely distributed than data. Thus, the model does not perfectly reproduce the data, and better model performance may be achieved by increasing the number of states or collecting more trajectories in the sparsely distributed region. Besides, the false k-nearest neighbor probability is based on a k-nearest neighbor search in the sense of mean square Euclidean distance. Different types of distance may reveal other characteristics of the similarity between the trajectory ensembles. Furthermore, the optimal number of states from the relative KL-divergence is slightly larger than that of false k-nearest neighbor probability. It implies that the relative KL-divergence and the false k-nearest neighbor probability are not correlated and serve as separate measures on the training performance.

3. Rescaled likelihood.

We quantified the likelihood for each condition as follows. For each condition, we randomly picked 30 trajectories as test dataset and the remaining as training dataset. We then trained a hidden Markov model by the training dataset, and got the trajectory probability of the test dataset based on the model. The likelihood is defined as log of the trajectory probability for the test dataset.

According to the formula of trajectory probability Eq. (S2.3), one diagonal element of matrix D was multiplied after each time step. As the elements in matrix D approximately scale inversely with the number of emission states, the trajectory probability decays with the inverse number of emission states to the power of time points, i.e, $(1/B)^n$, where B is the number of emission states and n is the current time point. In order to compare the likelihood with using various number of emission states, we need to rescale the trajectory probability with respect to the number of emission states. Otherwise, the trajectory probabilities are not directly comparable, as their decay rates vary with the number of emission states.

The rescaling procedure multiplies the trajectory probability by the factor B^n at the time point n. For each trajectory in the test dataset $\tilde{y}_{1:n}$, the rescaled likelihood is:

$$\mathit{Likelihood}(\tilde{y}_{1:n}) = \log_2[p(\tilde{y}_{1:n}) * B^n] = \log_2 p(\tilde{y}_{1:n}) + n \log_2 B. \tag{S2.7}$$

The rescaling term $n \log_2 B$ serves as a likelihood for the null hypothesis of the hidden Markov model. It increases logarithmically with respect to the number of emission states. The rescaled likelihood is a logarithm function on the ratio between the estimated trajectory probability and that from the null hypothesis.

After scaling, we took the likelihood at the last time point for each trajectory during the time course. We plot this likelihood versus the number of states, as shown in Fig. S4. The rescaled likelihood tends to increase with the number of emission states. It demonstrates that the likelihood of the test dataset generated from the hidden Markov model keeps improving. However, as the number of states increases, the parameters may become abundant. It then leads to an overfitting issue on estimating the dMI, as shown in Fig. S5. Thus, the proper number of states should not be too large.

Since the likelihood is rescaled, its absolute value does not have a specific meaning. Instead, it is a measure to show the relative training performance of using different number of states. If the rescaling procedure was not used, the log-likelihood keeps decreasing with the number of the states, as dominated by the decay of the trajectory probability. Besides, as the training dataset only has 30 trajectories, which is much less than the original dataset with around 500 trajectories, the training performance on the hidden Markov model is largely preserved when comparing with that trained by all the trajectories.

The mutual information does not depend on this rescaling procedure, because the term $n \log_2 B$ in the conditional and unconditional entropy cancel each other. Still, this term separately rescales the conditional and

unconditional entropy, as they both keep growing with respect to time points due to the decay of trajectory probability.

The rescaling method is supportive to use the trajectory entropy without the prefactor $1/n$, i.e., $H(y_{1:n}) = -\log_2 p(y_{1:n})$ instead of $\tilde{H}(y_{1:n}) = -(1/n)\log_2 p(y_{1:n})$ at each time point n. The latter was previously used to get a converged value of the entropy rate as comparable to the first Lyapunov exponent [13–16]. In those formulations, the prefactor $1/n$ was employed to counterbalance the decay of trajectory probability. Here, we have used an alternative method to rescue the issue of probability decay by the rescaling procedure. The advantage of the current method is that the mutual information does not depend the rescaling procedure, and thus is able to reveal the time-dependence of the mutual information without the alteration by the prefactor $1/n$, cf. Sec. II D 2.

When training the hidden Markov model for different conditions, the number of states may be chosen differently. For certain conditions, to train a good hidden Markov model may require more numbers of states than the others. Then, one needs to rescale the trajectory probability accordingly to keep the trajectory probability comparable across conditions. To avoid this complexity, we preferred to keep using the same number of states for all the conditions, and rescale the trajectory probabilities uniformly.

4. Training performance with scanning the number of states.

We scanned the number of states to plot the relative KL-divergence, the false k-nearest neighbor probability, and the rescaled likelihood. We first kept the ratio of emission states and transition states constant, as 1 or 2 separately. We further independently varied the number of hidden states and emission states. The number of scanned states is chosen in log-scale.

In all the scans, the optimal false k-nearest neighbor probability and the relative KL-divergence are reached when using around 32 emission states and $[32, 64]$ hidden states for most stimulus conditions. The rescaled likelihood of test dataset tends to increase as the number of states increases. However, as shown in Sect. II B 3 below, when the number of hidden states further increases, it leads to pseudo high maximum mutual information with reaching the overfitting regime. Based on these results, we chose 64 hidden states and 32 emission states as an ideal number of states for model training. The variation on the number of states in certain range may also be acceptable.

For certain conditions having less dynamical patterns, such as the control condition without stimulus, the number of states may be abundant. The relative KL-divergence may even start to increases with the number of states. This suggests to use less numbers of states for the control condition. However, as demonstrated in Rescaled likelihood of Sect. II B 2, distinct number of states would lead to the issue of inconsistent scaling on the trajectory probabilities across the various conditions. Considering that the training performance of control condition does not become too worse under 64 hidden states and 32 emission states, we chose to use the same number of states for all the conditions.

5. Discussion on the conditions with lower performance of model training.

The trained hidden Markov model is not always perfect to reproduce the trajectories for certain conditions. We have checked the conditions with large relative KL-divergence. Those conditions typically have less trajectories, which may be insufficient to train an accurate hidden Markov model. When the number of trajectories is larger than 500, the model training has higher accuracy. We thus suggest to apply the hidden Markov model to the datasets with sufficient number of trajectories. Alternatively, one may exclude some conditions by using the above measures on the training performance, such as setting a lower threshold for the false k-nearest neighbor probability to exclude certain conditions.

For the conditions with oscillations, the sampled trajectories may not accurately generate trajectories with exactly the same oscillation frequencies. It is due to that the oscillation frequencies have a continuous spectrum, and there is a lack of trajectories with the less abundant frequencies to train a good hidden Markov model.

3. *Estimation on the dynamical mutual information*

In this subsection, we provide the procedure to numerically calculate the trajectory probability, the conditional and unconditional trajectory entropy, the dMI and its maximization. We then use the Jackknife method to evaluate the effect of the subsample size of trajectories on the maximum mutual information. We next carefully investigate how the overfitting of model affects the result, by calculating the maximum mutual information for equal-partitioned data within each condition. We further discuss the dependence of mutual information on the number of hidden states,

emission states, and time points, and sampled trajectories. In the end, the dMI between various pairwise conditions show distinct information transmission rates for discriminating the stimuli.

1. The trajectory probability.

   Quantifying the probability in the trajectory space is challenging, because there are infinity configurations in the path space [19]. Even after discretizing the path space into finite states, we need sufficient amount of the data to learn the exponentially increasing transitions between the states. For the hidden Markov model, we extracted the most likely transition and emission matrices for each condition.

   When calculating the trajectory probability by Eq. (S2.3), we have used the initial uniform distribution for the hidden states. The trajectory probability is then obtained by multiplying the distribution for the hidden states to the transition matrices and emission probability based on the measured time series, as in Eq. (S2.3). We have checked that the resultant maximum mutual information is not sensitive to the chosen initial distribution, where we have tested another choice based on the first time-point measurements.

   The summation over all the possible trajectories for the hidden Markov model is 1, if including all the emission states. This normalization property also holds for the Markovian model discussed in Sect. III below. On the other hand, in Eq. (S2.3) the trajectory probability $p(y_{1:n})$ is estimated for each observed time series $y_{1:n}$. Thus, we need to get the trajectory probability in a trajectory-wise manner, which does not include the procedure of summing over the trajectory configurations. The trajectory probability $p(y_{1:n})$ can be interpreted as the posterior probability of each trajectory $y_{1:n}$ based on the trained model. Calculating the trajectory probability is equivalent to a sampling procedure, and under the trained model each trajectory has a certain probability, which is typically smaller than 1.

2. Unconditional trajectory probability.

   In the above, we have calculated the probability for observing each trajectory under the model of each stimulus. Thus, we already have all the conditional trajectory probability. The unconditional probability requires more care. It was obtained by summing over the conditional trajectory probability of each stimulus. The conditional trajectory probability is based on various stimuli, and each condition has a weight. We applied a weight distribution on all the conditions, and then maximized the mutual information with respect to this weight distribution, as in Eq. (S1.9). With the conditional and unconditional trajectory entropy, we now have the mutual information Eq. (S1.8).

3. The maximum dynamical mutual information.

   After getting the dMI, we maximized it through optimizing the stimuli's distribution to get Eq. (S1.9). The maximum dMI was obtained for each time point, accounting for the information from full dynamics of the signaling responses up to that time point. With sufficiently number of stimuli, the dMI becomes the channel capacity, corresponding to the maximum capacity of information transmission for this signaling channel. We conducted the optimization at each time point separately, and finally got the time course of the maximum dMI. The optimization was done by the interior-point method, with using the MATLAB function fmincon.

   There can be trajectories with $p(R^i_{1:n} = y^{i,j}_{1:n}|S = k) \leq p(R^i_{1:n} = y^{i,j}_{1:n}|S = i)]$ breaking down, which may lead to a negative maximum mutual information exceeding the lower bound in Eq. (S1.13). To avoid this pseudo negativity, we changed those trajectory probabilities conditioned on the other conditions, which is larger than that conditioned on its original condition, to the latter with multiplying a penalty factor. We have chosen a penalty factor to be 10, such that it leads to a positive maximum mutual information along the time course. It means that once the trajectory probability conditioned on the other conditions is not 10-times larger than that conditioned on its original condition, the former is kept in Eq. (S1.8). Otherwise, we use the latter to avoid the negative maximum mutual information. Since the number of the trajectories without $p(R^i_{1:n} = y^{i,j}_{1:n}|S = k) \leq p(R^i_{1:n} = y^{i,j}_{1:n}|S = i)]$ only takes a small ratio of the total trajectories, around 10 percent, this penalization does not qualitatively affect the calculation on the maximum mutual information.

4. Quantifying overfitting by the equal-partition within each condition.

   The dMI is dependent on the number of states. There is a possibility of overfitting when the states are abundant, which will give unreasonably higher dMI. To quantify the degree of overfitting, we conducted an equal-partition procedure to calculate the maximum mutual information. The goal is to clarify the ability of distinguishing the difference between various stimuli and that from the noise within each stimulus. Thus, we calculated the maximum mutual information between the two subsets of trajectories within each condition, to see whether they are recognized as different conditions by the model under a certain number of states. The maximum mutual

information between the two subsets would be nonzero if the model is overfitted. The procedure is thus able to uncover whether the number of states is abundant.

Specifically, we separated trajectories of each condition into two subsets with equal number of trajectories. Then, we trained hidden Markov models for the two subsets separately, and calculated the maximum mutual information when only using these two subsets. If they are indistinguishable, the maximum mutual information should be 0 bit; If they are totally distinguishable, the maximum mutual information should be 1 bit. Since the two subsets are from the same stimulus' condition, they should lead to 0 bit of maximum mutual information in the ideal limit, unless overfitting happens. We then repeatedly calculated the maximum mutual information under different number of states. The critical number of states leading to nonzero maximum mutual information indicates a threshold of reaching the overfitting regime.

We further noticed that the equal-partition of each condition's data gives less trajectories for each model training. The trained model thus becomes less accurate due to a lack of trajectories, and the overfitting problem may be worse. To account for the effect of this issue, we used half the number of trajectories to calculate the maximum mutual information with employing all the stimuli. The maximum mutual information does become larger compared with using the total number of trajectories, as shown in Fig. S13. The equal-partition within each condition has the same issue and generates an over-estimated maximum mutual information, which may make the critical number of states smaller. We thus rescaled the maximum mutual information of the equal-partition procedure, through multiplying the ratio between the maximum mutual information when using all stimuli's total trajectories and half of them.

We plotted the rescaled maximum mutual information versus the number of hidden states, with showing the mean and standard deviation of the results from all conditions in Fig. S5. We have considered the number of states in log-scale: $[2, 4, 8, 16, 32, 64]$, and found the critical number should be in the range of $[32, 64]$. We then scanned linearly the number of states in that range: $[30, 40, 50, 60]$. Overall, overfitting starts to happen with using more than 40 hidden states, and causes around 0.1 bit pseudo information around 60 hidden states. Considering the training performance versus the number of hidden states, we thus have a trade-off issue: less hidden states generate relatively lower training performance but avoid overfitting; more hidden states cause the overfitting problem to give an unreasonable maximum mutual information. However, there is a regime with around $[32, 64]$ hidden states that can balance the two goals simultaneously, as illustrated in Fig. S5. For the purpose of making the heatmaps better visualized, we chose 64 hidden states and 32 emission states as the optimal for the NFκB dataset.

5. The dynamical mutual information with randomly permutating timepoints.

For the present method, we first conducted permutation on the ordering of full time series. We chose random ordering by using the MATLAB function randperm for one trajectory. We then applied the same order of permutation to all the other trajectories. The permutation on the ordering of time points change the dynamical pattern in the trajectory configuration. Thus, it affects the maximum mutual information obtained through the present method, as shown in Fig. 3.

The maximum dMI after the random permutation of timepoints increases over time beyond 2 hours, because the trajectory ensembles under various stimulus conditions can still be discriminated. For example, as shown in Fig. S11, the trajectory ensembles after random permutation have differences after 2 hours. There are trajectory ensembles with many high (over 5), middle (around 4) and low (below 3) response amplitudes. The differences are revealed by the maximum dMI of the permuted data that continues to rise over 1 bit (Fig. 3).

For the vector method discussed below, we first chose a certain number of points as the multi-dimensional vector. For example, at the time point n, the first n points of each trajectory are taken as a vector for further analysis. We then permuted the data points with random order and all the vectors were permuted with the same order. As shown in Fig. 3, the random ordering does not alter the estimated maximum mutual information through the vector method. The reason is that the method employs the k-nearest neighbor density estimator to get the trajectory probability, where the distance is taken as the sum of mean square between the data points of each two vectors. Then, the permutation on the ordering of time points does not change the distance between any two vectors.

We remark that in Fig. 3 we have permuted the time points uniformly for all the conditions. Alternatively, we permuted the time points non-uniformly for each trajectory, that is, every trajectory has the time points permuted with a random order separately, and thus the vector-method was also altered. This permutation totally disrupts the dynamical patterns of the trajectory ensembles under each condition. We then applied the various methods to this permuted dataset, and the mutual information is around 1 or 2 bits by the methods (Fig. S12). The information comes from that the trajectory ensembles have different peak amplitudes and

integrals on average. For example, the control condition has low peak amplitudes and low integrals, the TNF condition has middle peak amplitudes and middle integrals, and the LPS condition has high peak amplitudes and high integrals, etc. Thus, there are still 1 or 2 bits of information embedded in the permuted trajectory ensembles, as revealed by the various methods to certain extent.

6. The dynamical mutual information for pairwise conditions.

   We also studied the dMI with using pairwise conditions, labeled by stimulus identity and concentration. The dMI for pairwise stimuli uncovers the maximum capacity to distinguish these two stimuli by the signaling channel in a temporal manner. From the result of the dMI between each pairwise conditions, we found different modes of temporal dynamics for the information transmission, as shown in Fig. 4.

   The dMI here may be connected to downstream gene expression which are mainly responsive to the NFκB signaling channel. For example, for the two conditions with zero maximum mutual information in the first 6 hours and nonzero after that, we expect that the NFκB-responsive genes express more differently in the late time window, because they can not decode the information to distinguish the two stimuli in the early time window.

7. The dynamical mutual information for the pairwise stimulus conditions with using replicates.

   To perform an out-of-sample cross-validation on the dMI, we calculated the dMI for the pairwise stimulus conditions (Fig. 4), and used the replicate data to replace one of the two in each pairwise conditions. The replicates were generated at different dates (Table S3). As shown in Fig. S17, the dMI with using the replicates are similar to that in Fig. 4, validating the robustness of dMI calculations.

   There are differences in the dMI values between the replicates. The deviations are small for the first two pairwise conditions. For the third pairwise stimulus conditions (third row in left panel, red line) in Fig. S17, though both dMI of the replicates gradually increase in the relatively late phase, the onset time differs. This can be attributed to the fact that the measured data under 100 nM CpG varies between the replicates, as displayed by the upper heatmap in the red box of Fig. 4 and Fig. S17. In any case, the dMI robustly reveals such difference. Overall, the out-of-sample measurements may have certain variations, which are captured by the dMI method.

8. The dependence of dynamical mutual information on the stimuli distribution.

   In Eq. (S1.9), we have maximized the dynamical mutual information with respect to the probability distribution of the stimulus conditions $\mathbf{q} = \{q_1, q_2, \ldots, q_M\}$. The maximum dMI is used to quantify the maximum ability of the cells for stimuli-discrimination. The maximization procedure gives the optimal distribution that can bias to certain stimulus conditions. The bias indicate that certain stimulus conditions are more distinguishable by cells than others, as cells are more likely to differentiate the conditions with high distributed values.

   As a comparison, we also use the uniform distribution of stimulus conditions to calculate the dMI for both Fig. 3 and Fig. 4. The optimal distribution leads to higher maximum dMI than the uniform distribution, as shown in Fig. S18. The maximum dMI in Fig. 3 is much higher than that from uniform stimuli distribution, demonstrating that the maximization leads to better information transmission when discriminating many stimulus conditions. For the pairwise condition, the maximum dMI is only slightly higher than that from the uniform stimuli distribution, because the room to optimize the distribution of two stimuli is limited.

4. The dependence on the number of parameters, measured cells and time points

1. Jackknife method on the mutual information estimation.

   In order to estimate the maximum dMI for infinite size of the data, we applied the Jackknife method [5]. We took the trajectory probabilities from the trained hidden Markov model, i.e., sampled trajectory probabilities without replacement, and then used the sampled subset to calculate the maximum mutual information. We chose a series of ratios of the total data as subsets. The ratios are from 0.5 to 1 with step length 0.05. To avoid the bias by having insufficient number of trajectories, the ratio terminates at 0.5 with having half the number of the trajectories. We calculated the maximum mutual information for these subsets of data, given the trained model with 64 hidden states 32 and emission states.

   We then plotted the averaged maximum dMI relative to the inverse of the subsample size in Fig. S13**a**. The curve between the maximum dMI and sample sizes is basically flat. The y-axis intersection serves as an estimation on the maximum mutual information with an infinite number of the data. As the y-axis intersection is nearly the same as that with using all the data, the rescaled maximum dMI by the Jackknife method almost does not change. This Jackknife procedure was included in the simulation for other mutual information estimations.

We also repeated the random sample on the trajectory probabilities and the Jackknife procedure for 10 times. We plotted the standard deviation of the Jackknife fitted curve between the averaged maximum dMI and the inverse of the subsample size (Fig. S13**a**). The range of the y-intersection of the Jackknife fitted curve provides an estimated range of averaged maximum dMI when the data is infinitely measured. The tiny standard deviation of the replication indicates that the estimation on the dMI depends insensitively on the sampled trajectory probabilities after the model was trained by all the data.

Here, we did not use the subset of the data to re-train the hidden Markov model, as less data causes more serious overfitting issue on the model training and leads to unreasonable higher maximum mutual information, cf. Sect. II B 3. We thus directly used the trajectory probabilities from the same trained model to avoid the alternation of the overfitting issue on the Jackknife method. The treatment assumed that a model has been well trained to represent each condition, and further investigated how the sample size of trajectories affects the calculation on the trajectory probability and mutual information.

2. The dynamical mutual information versus the number of emission states.

   The dMI depends on the number of emission states, as shown in Fig. S13. The trajectory probability varies with the number of emission states according to Eq. (S2.2), and the variation occurs similarly across all the conditions. The trajectory configurations of the transitions between the hidden states to generate the trajectories do not increase. Thus, the dMI does not dramatically change with the number of emission states once it is sufficient for model training. Here, 32 emission state serves as an ideal number to generate well-trained hidden Markov models, as shown by the measures on the training performance in Fig. 2.

3. The dynamical mutual information versus the number of hidden states.

   As plotted in Fig. S13, the dMI varies with the number of hidden states. The dMI keeps increasing with number of hidden states, which will eventually reach the maximum theoretical limit, i.e., $\log_2$ of the number of conditions.

   When the number of hidden states becomes larger, more hidden states can emit the same value of the measured data. The trajectories become more distinguishable, as there are more possible transitions between hidden states to generate those trajectories. An increasing number of hidden states enables more configurations on the transitions between hidden states. Then, the models of generating the trajectory ensemble for all conditions become more distinguishable, which leads to higher mutual information. An analog of this issue is the entropy of a distribution: the entropy increases with the extent of coarse-graining.

   On the other hand, an increasing number of hidden states leads to the overfitting issue. The overfitting regime can not be uncovered from the measures of training performance, because the measures only show the performance of the model to reproduce data rather than overfitting. For example, the rescaled likelihood and the false k-nearest neighbor probability increase towards saturation versus the number of hidden states. They just tell the number of hidden states sufficient to train a good hidden Markov model. Therefore, we have developed another criterion to get the upper cutoff on the number of hidden states, i.e., the equal-partition procedure.

4. The proper number of states depends on the dataset.

   The proper number of states may differ across datasets. Specifically, the optimal number of states depends on the number of timepoints, the number of cells, the complexity of the dynamic trajectory, and the cell-to-cell heterogeneity. Therefore, we anticipate that the optimal number of states can be different for p53, ERK datasets, especially when the number of timepoints and cells differ. The best way to determine the optimal number of states is to follow the computational protocol in Fig. S9, which is applicable to various datasets with distinct dynamical patterns.

   We have used the same number of states for the various stimulus conditions in the NFκB dataset. The primary reason is for the convenience of computation on the dMI. Specifically, to calculate conditional trajectory probabilities in Eq. S1.8 requires to use the model trained by condition 1's data to calculate the probabilities of condition 2's trajectory. Then, the same number of states allows a direct insertion of condition 2's trajectory to condition 1's hidden Markov model for the calculation. If different state numbers are employed for each condition, more sophisticated procedures need to be designed to match the states. Besides, we found that, though the conditions have different number of trajectories that are differentially prone to overfitting, the variation of overestimated mutual information is in the scale of 0.1 bit, and does not dramatically affect the whole calculation. The errorbar in the grey points of Fig. S5 exactly show the standard deviation of the overestimated dMI across conditions. Therefore, we choose to use the same state number for all the conditions. If one needs to strictly rule out such difference across conditions, another solution would be to use the same number of trajectories for each condition.

5. The dynamical mutual information versus the number of time points.

   To further investigate the required number of timepoints, we performed dMI calculations with using a different sampling frequency, where every two time points of the NFκB dataset were used. As shown in Fig. S13, the dMI by using every two time points has a similar temporal profile as the original sampling frequency (Fig. 3), and is slightly higher. It indicates that the present sampling frequency, with $\sim$150 number of timepoints in 12 hours, may be reduced, but reducing half the number of measured cells starts to cause overfitting. We further used half the number of states for the subsampled data, which gives a lower dMI (Fig. S13). Thus, the optimal number of states for the subsampled data are between the two settings.

   Overall, $\sim$150 time points enable the existence of proper number of parameters for the NFκB dataset, serving as a reference for other datasets. For new datasets, one needs to search for the optimal parameter by the protocol (Fig. S9) and may in turn increase the number of measured timepoints.

6. The dynamical mutual information versus the number of measured cells.

   For the hidden Markov model, $\sim$500 cells are required for the NFκB dataset with $\sim$150 time points, in order to achieve high model performance and avoid overfitting for the dMI calculation. Overfitting happens if using half the number of the measured cells (Fig. S9).

   As for the time-inhomogeneous Markov model, it can be trained to reproduce the signaling dynamics even with $\sim$100 cells, such as shown in Figs. S2, S6, S7. However, to avoid overfitting such that the dMI will not be overestimated, the time-inhomogeneous Markov model requires many more cells. The NFκB, p53, and ERK datasets do not have sufficient cell numbers, such that the dMI is overestimated if the time-inhomogeneous Markov is used (Fig. S8). We note that here the dMI for p53 and MAPK datasets does not quantify the stimulus discrimination and is only used to check whether the overfitting happens. To avoid this overestimation demands at least several fold of 500 cells, because for each two consecutive time points one transition matrix needs to be fitted, whereas the hidden Markov model only requires two matrices in total. Therefore, in practice it is experimentally and computationally expensive to use the time-inhomogeneous Markov model for accurate dMI calculations, and the hidden Markov model is generally more suitable.

   The required number of cells also depends on the heterogeneity of the dataset. In general, more measured cells are required for more heterogeneous signaling dynamics, such that the heterogeneous dynamics can be captured by the model. For a given number of cells, one needs to search for the optimal number of parameters that allow high model performance and avoid overfitting, as shown in Fig. S5. When the dataset is more heterogeneous, to achieve high model performance will be harder. Then, the range for "number of states is sufficient to train a model" in Fig. S5 becomes narrower. When there is a gap on the proper number of parameters to have high model performance and avoid overfitting simultaneously, more cells need to be measured, which constitutes a criterion for the required number of cells.

7. The dynamical mutual information versus the time window of data.

   The number of time points used to train the hidden Markov model also affects the model training and the overfitting issue. The usage of less time points leads to lower accuracy on the model training and higher maximum mutual information due to the potential overfitting. To demonstrate the effect, we used time course of $2, 4, 6, 8, 10, 12$ hours to train hidden Markov models and calculate the dMI. As plotted in Fig. S13, the dMI is higher when using shorter time course within that time window. For the range out of the chosen time window, as shown by transparent lines, the dMI starts to decrease with time. In that time regime, the trajectories are less distinguishable by the model, because the model was not trained by the data of that time regime.

   The higher dMI by using shorter time course can be attributed to the overfitting issue. The data in the shorter time course is insufficient for the model training, which causes an abundance on the number of states. The result with using shorter time course of the data points provides as an upper bound on the dMI under the chosen number of states. To accurately quantify the contribution of model overfitting to the dMI when using certain time course of the data, one needs to repeat the procedure of equal-partition.

   Besides, we calculated the dMI for a time window from 2 hours to 12 hours, by which the overfitting does not dramatically increase as most data is kept. As shown in Fig. S13, the dMI has a nonzero value at the beginning of 2 hours, which is lower than the result at 2 hours from the full time window. The reason is that the earlier time points provide information cumulatively for the full time window case. Note that the dMI from the late time window may be an overestimate due to the overfitting. Overall, the dMI from late time window quantifies the extent of discriminating stimuli in that time window, but does not reflect how cells cumulatively discriminate stimuli in the whole process after sensing the stimuli.

8. The dynamical mutual information for randomly selected trajectories from data.

   We conducted the bootstrap procedure with randomly sampling from the dataset with replacement. We used the sampled data as the new input for the whole procedure of model training and mutual information calculation. The dMI for the bootstrap replicates gives similar result as the original dataset (Fig. S13), with the largest difference around 20%. It indicates that the finitely measured trajectory configurations under each stimulus contain the most representative configurations (80%) in the trajectory space to be recognized by the model. The inaccuracy of model training also leads to certain extent of bias on the mutual information estimation.

C. The other relevant statistical quantities from the hidden Markov model

In this subsection, we provide other trajectory-wise formulations, including the conventional entropy rate for the hidden Markov model. These formulations were mostly established in the literatures [14–16], whereas the above formulation on quantifying the dMI has not been reached before. We found that the trajectory entropy Eq. (S1.2) is the proper one to quantify the maximum mutual information for distinguishing stimuli, as shown in Sect. II B 3. The other formulations below may find use in different applications.

1. The entropy rate and normalized trajectory probability

For completeness, we give the other formulations on the entropy rate.

1. Given the trajectory probability $p(y_{1:n})$, one can take the logarithm function of the trajectory probability and multiply a prefactor $1/n$

   $$\tilde{H}(y_{1:n}) = -\frac{1}{n}\log_2 p(y_{1:n}), \tag{S2.8}$$

   where $\tilde{H}$ denotes the entropy rate formula with the prefactor $1/n$. This entropy rate formula was widely used in literature, because it is compatible to the first Lyapunov exponent [13–16].

   For the hidden Markov model, this entropy rate is:

   $$\tilde{H}(y_{1:n}) = -\frac{1}{n}\log_2\Big[\mathbf{p}_0 TD(y_1)\cdots TD(y_n)\mathbf{I}\Big]. \tag{S2.9}$$

   According to this formula, one can have an approximation: $\log_2 \mathbf{p}_0 TD(y_1)\cdots TD(y_n)\mathbf{I} \sim n$. Then, with prefactor $1/n$, we get a finite number. This is one of the reasons for the entropy rate being popular, especially when the stationary trajectory is known.

   On the other hand, if given a trajectory for the stationary process, such as a sufficiently long stationary trajectory for an ergodic process, the entropy rate can also be calculated from the Lyapunov exponent [14, 15]. The first Lyapunov exponent is defined as:

   $$\lambda_1 \doteq \lim_{n\to+\infty}\frac{1}{n}\log_2 ||TD(y_1)\cdots TD(y_n)||, \tag{S2.10}$$

   where $||\cdot||$ is a matrix norm.

   To get the Lyapunov exponents, one can use the QR or LQ decomposition on the matrix product $TD(y_1)\cdots TD(y_n)$. For a given stationary trajectory, all the Lyapunov exponents can be similarly calculated as:

   $$\lambda_\beta \doteq \lim_{n\to+\infty}\frac{1}{n}\log_2 R_{\beta,\beta}(y_n), \tag{S2.11}$$

   where $R_{\beta,\beta}(y_n)$ is the (β,β) entry as the β-th largest eigenvalues for the QR or LQ decomposition of the matrix product.

   For an ensemble of trajectories, we can use the ensemble average to replace the long-time trajectory average, if the process is ergodic. Then, the Lyapunov exponent is calculated at each time point. From example,

   $$\lambda_\beta(n) \doteq \frac{1}{n}\langle\log_2 R_{\beta,\beta}(y_n)\rangle_{path}, \tag{S2.12}$$

   where $\langle\dots\rangle_{path}$ denotes the average over the trajectory ensemble. When the number of paths is sufficient, the first largest Lyapunov exponent at each time point n gives a time-dependent entropy rate.

2. As another method, one can get an entropy for the time series of the hidden states at each time point. It requires to calculate the probability of the hidden states conditioned on every observed trajectory: $\hat{p}(x_n|y_{1:n}) = p(y_{1:n}, x_n)/p(y_{1:n})$, where $p(y_{1:n}) = \sum_{x_n} p(y_{1:n}, x_n)$. The probability $\hat{p}(x_n|y_{1:n})$ is termed as the filtered probability [13]. The method does not directly give the probability or entropy for the observed time series in the trajectory ensemble. Still, it has a neat forward algorithm:

$$\begin{aligned}\hat{p}(x_n|y_{1:n}) &= \sum_{x_{n-1}} \frac{1}{p(y_{1:n})} E(y_n|x_n) T(x_n|x_{n-1}) p(x_{n-1}, y_{1:n-1}) \\ &= \sum_{x_{n-1}} \frac{p(y_{1:n-1})}{p(y_{1:n})} E(y_n|x_n) T(x_n|x_{n-1}) \hat{p}(x_{n-1}|y_{1:n-1}) \\ &\doteq \sum_{x_{n-1}} \hat{T}(x_n|x_{n-1}, y_{1:n-1}) \hat{p}(x_{n-1}|y_{1:n-1}). \end{aligned} \tag{S2.13}$$

We have introduced a transition kernel:

$$\hat{T}(x_n|x_{n-1}, y_{1:n-1}) \doteq \frac{p(y_{1:n-1})}{p(y_{1:n})} E(y_n|x_n) T(x_n|x_{n-1}). \tag{S2.14}$$

This method leads to a process given by a time-dependent transition kernel for the filtered probability, similar to the time-inhomogeneous Markovian model. Each observed trajectory generates a time-dependent trajectory entropy for the trajectory on the hidden states. The full trajectory ensemble is recovered by a series of time-dependent transition matrix $\{\hat{T}(x_n|x_{n-1}, y_{1:n-1})\}_{n=1}^{N}$. The probability of the trajectory with the observed time series $y_{1:N}$ is:

$$\hat{p}(x_{0:N}|y_{1:N}) = \Pi_{n=1}^{N} \hat{T}(x_n|x_{n-1}, y_{1:n-1}) \hat{p}(x_0|y_0). \tag{S2.15}$$

However, a crucial disadvantage of this method is that the transition kernel does not obey the normalization condition:

$$\sum_{x_n} \hat{T}(x_n|x_{n-1}, y_{1:n-1}) \neq 1, \tag{S2.16}$$

as the following equality does not hold in general:

$$\frac{p(y_{1:n})}{p(y_{1:n-1})} \neq \sum_{x_n} E(y_n|x_n) T(x_n|x_{n-1}). \tag{S2.17}$$

For any given initial state x_{n-1}, this equality is generally not satisfied. Consequently, we can not simplify its model-wise trajectory entropy as that of the time-inhomogeneous Markov model, i.e., Eq. (S3.5) in Sect. III B.

2. The model-wise statistics from the hidden Markov model

In this subsection, we provide the formulas of calculating the model-wise trajectory entropy for the hidden Markov model. They are based on the previous theoretical investigations on the hidden Markov model, and are distinct from the trajectory-wise statistics introduced above. We listed the terminology on the trajectory-wise and model-wise quantities in Table S2.

For a given stochastic dynamics, the model-wise trajectory entropy is defined as [20]:

$$\hat{H}[y(t)] = -\sum_{path} p[y(t)] \log_2 p[y(t)], \tag{S2.18}$$

where $\hat{H}$ represents the model-wise entropy and $p[y(t)]$ denotes the probability of a trajectory $y(t)$. The summation adds up all the possible trajectory configurations generated by the model, and is conducted with respect to the whole trajectory ensemble. If we sum up all the transition probabilities in the trajectory ensemble, we get the model-wise trajectory entropy for this trajectory ensemble.

For the hidden Markov model, summing over all the trajectory configurations can be inaccurate in practice, because the number of observed trajectories' configurations increases exponentially with time points, and each trajectory is generated by various configurations of the paths on the hidden states. Therefore, the trajectory probability is typically obtained for each observed trajectory [14, 15] and the summation of the entropy is conducted on the finitely measured trajectories, i.e., the trajectory-wise in Sect. I B rather than the model-wise.

D. A minimal model of the hidden Markov process

To exemplify the quantification on the trajectory entropy, the dMI and its maximization for the hidden Markov model, we study a minimal model [14]. Through the example, we found that the conventional entropy rate Eq. (S2.8) [13–16] with the prefactor $1/n$ leads to 0 bit of mutual information for two fully distinguishable trajectory ensembles. Instead, the trajectory entropy Eq. (S1.2) correctly gives a saturated 1 bit of maximum mutual information. Therefore, the trajectory entropy Eq. (S1.2), rather than the entropy rate Eq. (S2.8), is suitable for the mutual information calculation in this study.

The mutual information for trajectory ensembles generated by the minimum model has not been systematically investigated. It is also generally difficult to get analytical results on the trajectory entropy from the hidden Markov process, even for the minimal model considered here. In [14], they got an asymptotic approximation for the entropy rate, by using the generating functional. The approximation is valid only when the difference between the observable and hidden states are infinitely small. They also required the transition matrix of hidden states to have certain constrains, which may not be satisfied for general cases. Below, we analytically derive the long-time limit of the mutual information and numerically estimated the time course of the mutual information.

The system consists of a binary symmetric channel whose input is a first-order binary Markov model. The transition matrix and emission matrix are chosen as:

$$T = \begin{pmatrix} 1-p_{01} & p_{01} \\ p_{10} & 1-p_{10} \end{pmatrix}, \tag{S2.19}$$

$$E = \begin{pmatrix} 1-\epsilon_{00} & \epsilon_{00} \\ \epsilon_{11} & 1-\epsilon_{11} \end{pmatrix}, \tag{S2.20}$$

where the parameters are constants in the range of $[0, 1]$. For the purpose of demonstration, we further considered a simplified symmetric binary channel with the transition matrix:

$$T = \begin{pmatrix} 1-p & p \\ p & 1-p \end{pmatrix}, \tag{S2.21}$$

and assumed that the emission rate is independent of the hidden states:

$$E = \begin{pmatrix} 1-\epsilon & \epsilon \\ \epsilon & 1-\epsilon \end{pmatrix}. \tag{S2.22}$$

The process is then completely characterized by the parameters p, ϵ.

The forward probability for the trajectory $y_{1:n}$ is given by the recursion relation:

$$\begin{aligned} P(y_{1:n}) &= P(x_n, y_{1:n}) + P(\bar{x}_n, y_{1:n}) \\ &= P(y_n|x_n)P(x_n|x_{n-1})P(x_{n-1}, y_{1:n-1}) + P(y_n|x_n)P(x_n|\bar{x}_{n-1})P(\bar{x}_{n-1}, y_{1:n-1}) \\ &\quad + P(y_n|\bar{x}_n)P(\bar{x}_n|x_{n-1})P(x_{n-1}, y_{1:n-1}) + P(y_n|\bar{x}_n)P(\bar{x}_n|\bar{x}_{n-1})P(\bar{x}_{n-1}, y_{1:n-1}), \end{aligned} \tag{S2.23}$$

where the symbol $\bar{x}$ denotes the other state complementary to x. The above formula can be written as a matrix form:

$$\begin{aligned} &[P(x_n, y_{1:n}), P(\bar{x}_n, y_{1:n})] \\ &= [P(x_{n-1}, y_{1:n-1}), P(\bar{x}_{n-1}, y_{1:n-1})] \begin{pmatrix} P(y_n|x_n)P(x_n|x_{n-1}) & P(y_n|\bar{x}_n)P(\bar{x}_n|x_{n-1}) \\ P(y_n|x_n)P(x_n|\bar{x}_{n-1}) & P(y_n|\bar{x}_n)P(\bar{x}_n|\bar{x}_{n-1}) \end{pmatrix} \\ &= [P(x_{n-1}, y_{1:n-1}), P(\bar{x}_{n-1}, y_{1:n-1})] \begin{pmatrix} P(x_n|x_{n-1}) & P(\bar{x}_n|x_{n-1}) \\ P(x_n|\bar{x}_{n-1}) & P(\bar{x}_n|\bar{x}_{n-1}) \end{pmatrix} \begin{pmatrix} P(y_n|x_n) & 0 \\ 0 & P(y_n|\bar{x}_n) \end{pmatrix}. \end{aligned} \tag{S2.24}$$

Then, it can be seen that this formula is just a special case of the forward probability given by Eq. (S2.2). The last matrix is a constructed random matrix, depending on the observed time series. If $y_n = x_n$, then

$$\begin{aligned} &[P(x_n, y_{1:n}), P(\bar{x}_n, y_{1:n})] \\ &= [P(x_{n-1}, y_{1:n-1}), P(\bar{x}_{n-1}, y_{1:n-1})] \begin{pmatrix} (1-\epsilon)P(x_n|x_{n-1}) & \epsilon P(\bar{x}_n|x_{n-1}) \\ (1-\epsilon)P(x_n|\bar{x}_{n-1}) & \epsilon P(\bar{x}_n|\bar{x}_{n-1}) \end{pmatrix} \\ &= [P(x_{n-1}, y_{1:n-1}), P(\bar{x}_{n-1}, y_{1:n-1})] \begin{pmatrix} P(x_n|x_{n-1}) & P(\bar{x}_n|x_{n-1}) \\ P(x_n|\bar{x}_{n-1}) & P(\bar{x}_n|\bar{x}_{n-1}) \end{pmatrix} \begin{pmatrix} 1-\epsilon & 0 \\ 0 & \epsilon \end{pmatrix}. \end{aligned} \tag{S2.25}$$

The random matrix will be different when $y_n = \bar{x}_n$.

For the symmetric binary Markovian process, we further have the simplification:

$$\begin{aligned}
&[P(x_n, y_{1:n}), P(\bar{x}_n, y_{1:n})] \\
&= [P(x_{n-1}, y_{1:n-1}), P(\bar{x}_{n-1}, y_{1:n-1})] \begin{pmatrix} 1-p & p \\ p & 1-p \end{pmatrix} \begin{pmatrix} 1-\epsilon & 0 \\ 0 & \epsilon \end{pmatrix} \\
&= [P(x_{n-1}, y_{1:n-1}), P(\bar{x}_{n-1}, y_{1:n-1})] \begin{pmatrix} (1-\epsilon)(1-p) & \epsilon p \\ (1-\epsilon)p & \epsilon(1-p) \end{pmatrix}. \qquad \text{(S2.26)}
\end{aligned}$$

The iterative formula of the trajectory probability distribution on the hidden states is thus given by the matrix in the last line. We will use this rule to calculate the forward trajectory probability below.

1. *The trajectory entropy*

Previously, the entropy rate for the trajectories in the hidden Markov model is given by Eq. (S2.9) [13–16]. One property for this entropy rate is that Eq. (S2.9) in the long-time limit have a direct connection to the first Lyapunov exponent of the system. Another technical aspect is that the trajectory probability Eq. (S2.3) inside the logarithm function converges to zero exponentially with the number of time points, cf. Rescaled likelihood of Sect. II B 2. Then, the long-time entropy rate may not converges without using the prefactor $1/n$. The prefactor $1/n$ counterbalance the probability decay, leading to a finite converged value for the entropy rate. These two aspects may be the underlying reason for the common usage of Eq. (S2.9) previously.

This entropy rate for the trajectory is different from the trajectory entropy Eq. (S1.2) mainly used in this paper. Below, we will provide the analytical details towards calculating the former, and then demonstrate the difference to the present trajectory entropy, as illustrated in Fig. S10. Indeed, it was when investigating the entropy rate in the minimal model, we realized that the mutual information estimation should not include the prefactor $1/n$, but need to directly employ the trajectory entropy Eq. (S1.2).

For the example of the above minimal model with the given transition and emission matrix, we checked the iteration relation in the trajectory probability:

$$\begin{aligned}
TD(y_1)TD(y_2) &= \begin{pmatrix} (1-\epsilon)(1-p) & \epsilon p \\ (1-\epsilon)p & \epsilon(1-p) \end{pmatrix} \begin{pmatrix} (1-\epsilon)(1-p) & \epsilon p \\ (1-\epsilon)p & \epsilon(1-p) \end{pmatrix} \\
&= \begin{pmatrix} (1-\epsilon)^2(1-p)^2 + (1-\epsilon)\epsilon p^2 & (1-\epsilon)\epsilon(1-p)p + \epsilon^2(1-p)p \\ (1-\epsilon)^2(1-p)p + (1-\epsilon)\epsilon(1-p)p & (1-\epsilon)\epsilon p^2 + \epsilon^2(1-p)^2 \end{pmatrix}. \qquad \text{(S2.27)}
\end{aligned}$$

According to this derivation, it is not straightforward to find an analytical recursion relation. Therefore, we further simplified the model by specifying the value of ϵ or p, and considered the two cases below.

1. As the first example, let us further simplify the model by assuming $\epsilon = 0.5$. Then, the matrices T and D are commutable. We thus only need to find recursion relation for:

$$\begin{aligned}
TD(y_1)TD(y_2) &= 0.5^2 \begin{pmatrix} (1-p) & p \\ p & (1-p) \end{pmatrix} \begin{pmatrix} (1-p) & p \\ p & (1-p) \end{pmatrix} \\
&= 0.5^2 \begin{pmatrix} (1-p)^2 + p^2 & (1-p)p + (1-p)p \\ (1-p)p + (1-p)p & p^2 + (1-p)^2 \end{pmatrix} \\
&= 0.5^2 \begin{pmatrix} 1-2p+2p^2 & 2(1-p)p \\ 2(1-p)p & 1-2p+2p^2 \end{pmatrix}. \qquad \text{(S2.28)}
\end{aligned}$$

   Since it is still not obvious to have a recursion relation, we further simplify the example to have $p = 0.5$. Then,

$$P(y^1_{1:n}|M1) = 0.5^n \mathbf{p}_0 \begin{pmatrix} 1/2 & 1/2 \\ 1/2 & 1/2 \end{pmatrix} \mathbf{I} = 0.5^n, \qquad \text{(S2.29)}$$

   with assuming initial equal distribution. The superscript on the trajectory $y_{1:n}$ denotes the index of the hidden Markov model used to generate the trajectory. The symbol "M1" represents the model 1. For this example, any observed time series has the same trajectory probability.

   In order to conduct analytical derivation, we have made several simplifications. As different p is required to generate different hidden Markov models, this example is too special to help understand the maximum mutual information. To have a case that can generate distinct time series, we now move to the second example.

2. For the second example, we choose $\epsilon = 0$:

$$TD(y_1) = \begin{pmatrix} (1-\epsilon)(1-p) & \epsilon p \\ (1-\epsilon)p & \epsilon(1-p) \end{pmatrix} = \begin{pmatrix} (1-p) & 0 \\ p & 0 \end{pmatrix}, \tag{S2.30}$$

and for the other emission value:

$$TD(\bar{y}_1) = \begin{pmatrix} \epsilon(1-p) & (1-\epsilon)p \\ \epsilon p & (1-\epsilon)(1-p) \end{pmatrix} = \begin{pmatrix} 0 & p \\ 0 & 1-p \end{pmatrix}. \tag{S2.31}$$

Thus, the product of matrices for two time steps is:

$$TD(y_1)TD(y_2) = \begin{pmatrix} (1-p)^2 & 0 \\ (1-p)p & 0 \end{pmatrix} = (1-p)\begin{pmatrix} 1-p & 0 \\ p & 0 \end{pmatrix}, \tag{S2.32}$$

$$TD(\bar{y}_1)TD(\bar{y}_2) = \begin{pmatrix} 0 & p(1-p) \\ 0 & (1-p)^2 \end{pmatrix} = (1-p)\begin{pmatrix} 0 & p \\ 0 & 1-p \end{pmatrix}, \tag{S2.33}$$

$$TD(\bar{y}_1)TD(y_2) = \begin{pmatrix} 0 & p \\ 0 & 1-p \end{pmatrix}\begin{pmatrix} 1-p & 0 \\ p & 0 \end{pmatrix} = \begin{pmatrix} p^2 & 0 \\ (1-p)p & 0 \end{pmatrix} = p\begin{pmatrix} p & 0 \\ 1-p & 0 \end{pmatrix}, \tag{S2.34}$$

$$TD(y_1)TD(\bar{y}_2) = \begin{pmatrix} 1-p & 0 \\ p & 0 \end{pmatrix}\begin{pmatrix} 0 & p \\ 0 & 1-p \end{pmatrix} = \begin{pmatrix} 0 & (1-p)p \\ 0 & p^2 \end{pmatrix} = p\begin{pmatrix} 0 & 1-p \\ 0 & p \end{pmatrix}. \tag{S2.35}$$

To get a different behavior of the trajectory entropy than the first example, we will consider different values of p. There are recursion rules for the different combinations of the observed time series:

$$yy \to (1-p)y, \tag{S2.36}$$

$$\bar{y}\bar{y} \to (1-p)\bar{y}, \tag{S2.37}$$

$$\bar{y}yy \to (1-p)\bar{y}y = (1-p)p\begin{pmatrix} p & 0 \\ 1-p & 0 \end{pmatrix}, \tag{S2.38}$$

$$\bar{y}y\bar{y} \to p^2\bar{y}, \tag{S2.39}$$

$$y\bar{y}\bar{y} \to (1-p)y\bar{y} = (1-p)p\begin{pmatrix} 0 & 1-p \\ 0 & p \end{pmatrix}, \tag{S2.40}$$

$$y\bar{y}y \to p^2 y. \tag{S2.41}$$

We noticed that the probability depends on how many the hidden state's change (flip) happens. If the hidden state flips, we gain a probability p, and otherwise we gain a probability $1-p$.

Assuming the initial state is equally distributed, and then we only need to count the state changes. For a time series of length n, and with m-times state changes, its trajectory probability is:

$$P(y^2_{1:n}|M2) = 0.5p^m(1-p)^{n-1-m}, \quad m \leq n-1. \tag{S2.42}$$

If the state's changes happen under a fixed ratio, then the long-time limit of trajectory probability will converge.

2. *The dynamical mutual information*

To validate the formula Eq. (S1.8) on calculating the mutual information for the hidden Markov model, we applied it to the above two cases of the minimal model. Below, we first demonstrate that Eq. (S2.9) with the prefactor $1/n$ does not give the correct value of the maximum mutual information. On the other hand, Eq. (S1.8) is able to give the correct dMI for two distinguishable time series, where the maximum mutual information should be 1 bit. Depending on the degree of difference between the two examples above, the temporal profiles of the maximum dMI are distinct. Specifically, more distinguishable time series has the maximum mutual information reaching to 1 bit faster than the less distinguishable ones. The result supports to use Eq. (S1.8), which does not have the prefactor $1/n$ in the trajectory entropy formula.

We also noticed that both the conditional and unconditional entropy increase in the example, but their difference gives correct 1 bit of mutual information. For trajectories under one condition, its conditional trajectory probability

decays much slower than the cross-conditioned on the other model. For the NFκB dataset, this is also true between the conditions which are distinguishable from each other.

In detail, we considered two kinds of time series, which were generated from the two examples above. The first example gives a set of time series with roughly half of the states being state 1 and half in state 2, and half of states flips the state in the next time step. The second example also gives a time series with roughly half of them being state 1 and half in state 2, but only has probability p of state change at each time step. An illustration is given in Fig. S10. The emission probability $\epsilon = 0$ exactly transmits the hidden states' transitions to the observed time series, whereas $\epsilon = 0.5$ smooth out a bit the transitions of hidden states.

To calculate the conditional trajectory entropy, we rewrote the conditional trajectory probability,

$$P(y^1_{1:n}|M1) = 0.5^n, \tag{S2.43}$$

$$P(y^2_{1:n}|M2) = 0.5p^{n_1}(1-p)^{n-1-n_1}, \quad n_1/n \to p, \tag{S2.44}$$

where n_1 is the number of the states' flips in the time series.

We next evaluated the conditional entropy rate by taking the trajectory ensemble average. For the first model,

$$\tilde{H}(R^1_{1:n}|M1) = \mathbf{E}\Big\{-\frac{1}{n}\log_2[0.5^n]\Big\} = 1, \tag{S2.45}$$

where $R^1_{1:n}$ denotes the trajectory ensemble from the first model up to time point n and $\mathbf{E}$ represents the trajectory ensemble average. For the second model,

$$\begin{aligned}\tilde{H}(R^2_{1:n}|M2) &= \mathbf{E}\Big\{-\frac{1}{n}\log_2[0.5p^{n_1}(1-p)^{n-1-n_1}]\Big\}\\ &= \mathbf{E}\Big\{\frac{1}{n} - \frac{n_1}{n}\log_2 p - \frac{n-1-n_1}{n}\log_2(1-p)\Big\}\\ &= \mathbf{E}\Big\{\frac{1+\log_2(1-p)}{n} - \log_2(1-p) - \frac{n_1}{n}\log_2[p/(1-p)]\Big\}.\end{aligned} \tag{S2.46}$$

If we chose $p = 0.1$,

$$\begin{aligned}\tilde{H}(R^2_{1:n}|M2) &\approx \mathbf{E}\Big(\frac{1}{n} + 3.3219\frac{n_1}{n} + 0.1520\frac{n-1-n_1}{n}\Big)\\ &\to 3.3219 * 0.1 + 0.1520 * 0.9\\ &= 0.4690.\end{aligned} \tag{S2.47}$$

Here, we got a converged value of the entropy rate $\tilde{H}$, as the frequency of switching converges under the ensemble average. For the short-time regime, the non-normalized initial probability will affect the calculation. Thus, we assumed to have a uniform and normalized initial distribution in the examples here.

With the probability distribution of the two model, $q_i (i = 1, 2)$, the total conditional entropy is:

$$\begin{aligned}\tilde{H}(R_{1:n}|M) &\doteq q_1\tilde{H}(R^1_{1:n}|M1) + q_2\tilde{H}(R^2_{1:n}|M2)\\ &= q_1 + q_2\Big\{\frac{1+\log_2(1-p)}{n} - \log_2(1-p) - \frac{n_1}{n}\log_2[p/(1-p)]\Big\}.\end{aligned} \tag{S2.48}$$

For each given p, we can further simplify the formula. For example, with $p = 0.1$,

$$\begin{aligned}\tilde{H}(R_{1:n}|M) &\approx q_1 + q_2\Big(\frac{0.848}{n} + 0.4690\Big)\\ &\doteq q_1 + q_2 c_2\end{aligned} \tag{S2.49}$$

where we have taken the ensemble average in the whole time regime with $n_1/n = 0.1$, and introduced the auxiliary parameter c.

To calculate the unconditional trajectory entropy, we need to derive the cross-conditional trajectory probability:

$$P(y^2_{1:n}|M1) = 0.5^n, \tag{S2.50}$$

$$P(y^1_{1:n}|M2) = 0.5p^{n_2}(1-p)^{n-1-n_2}, \quad n_2/n \to 0.5. \tag{S2.51}$$

We then estimate the unconditional trajectory entropy as:

$$\tilde{H}(R_{1:n}) = \sum_{i=1}^{2} q_i \mathbf{E}\Big\{ -\frac{1}{n}\log_2\Big[q_1 P(y^i_{1:n}|M1) + q_2 P(y^i_{1:n}|M2)\Big]\Big\}, \tag{S2.52}$$

where $i = 1, 2$ denotes the index of the hidden Markov model employed to generate the trajectory. Then, for the above examples,

$$\begin{aligned}
\tilde{H}(R_{1:n}) &= q_1\mathbf{E}\Big\{ -\frac{1}{n}\log_2\Big[q_1 0.5^n + q_2 0.5 p^{n_2}(1-p)^{n-1-n_2}\Big]\Big\} \\
&\quad + q_2\mathbf{E}\Big\{ -\frac{1}{n}\log_2\Big[q_1 0.5^n + q_2 0.5 p^{n_1}(1-p)^{n-1-n_1}\Big]\Big\} \\
&\approx -q_1\frac{1}{n}\log_2\Big[q_1 0.5^n + q_2 0.5 p^{n/2}(1-p)^{n/2-1}\Big] \\
&\quad - q_2\frac{1}{n}\log_2\Big[q_1 0.5^n + q_2 0.5 p^{n/10}(1-p)^{9n/10-1}\Big] \\
&\doteq -q_1 d\log_2(q_1 a + q_2 b_1) - q_2 d\log_2(q_1 a + q_2 b_2),
\end{aligned} \tag{S2.53}$$

where we have made the approximation on the frequency of state change by using the ensemble average. The auxiliary functions a, b, c, d depend on n.

The mutual information from this type of the trajectory entropy $\tilde{H}$ is:

$$\tilde{I}(R_{1:N}; M) = \tilde{H}(R_{1:n}) - \tilde{H}(R_{1:n}|M). \tag{S2.54}$$

We can use the method of Lagrange multipliers to find the local maxima of the mutual information subject to the normalization condition $q_1 + q_2 = 1$. The Lagrange multiplier is:

$$LM \doteq -q_1 d\log_2(q_1 a + q_2 b_1) - q_2 d\log_2(q_1 a + q_2 b_2) - q_1 - q_2 c_2 - \Lambda_1(q_1 + q_2 - 1). \tag{S2.55}$$

By taking the derivative, we get:

$$0 = -d\log_2(q_1 a + q_2 b_1) - q_1 d\frac{a}{(q_1 a + q_2 b_1)} - q_2 d\frac{a}{(q_1 a + q_2 b_2)} - 1 - \Lambda_1, \tag{S2.56}$$

$$0 = -q_1 d\frac{b_1}{(q_1 a + q_2 b_1)} - d\log_2(q_1 a + q_2 b_2) - q_2 d\frac{b_2}{(q_1 a + q_2 b_2)} - c_2 - \Lambda_1, \tag{S2.57}$$

$$0 = q_1 + q_2 - 1. \tag{S2.58}$$

However, even for these two simple examples, the optimal probability is not analytically solvable under the given parameters a, b, c, d.

In the long-time limit, we can get an analytical approximation on the mutual information when $0 < p \ll 0.5$.

$$\begin{aligned}
\tilde{H}(R_{1:N}) &\approx -q_1\frac{1}{n}\log_2\Big[q_1 0.5^n + q_2 0.5 p^{n/2}(1-p)^{n/2-1}\Big] \\
&\quad - q_2\frac{1}{n}\log_2\Big[q_1 0.5^n + q_2 0.5 p^{pn}(1-p)^{(1-p)n-1}\Big] \\
&\approx -q_1\frac{1}{n}\log_2\Big[q_1 0.5^n\Big] - q_2\frac{1}{n}\log_2\Big[q_2 0.5 p^{pn}(1-p)^{(1-p)n-1}\Big] \\
&\approx -q_1\Big[\frac{1}{n}\log_2 q_1 - 1\Big] - q_2\Big[\frac{1}{n}\log_2[q_2(1-p)^{-1}] - \frac{1}{n} + \log_2 p^p(1-p)^{(1-p)}\Big]
\end{aligned} \tag{S2.59}$$

Then, in the long-time limit,

$$\begin{aligned}
\tilde{I}(R_{1:N}; M) &= \tilde{H}(R_{1:N}) - \tilde{H}(R_{1:n}|M) \\
&= -q_1\Big[\frac{1}{n}\log_2 q_1 - 1\Big] - q_2\Big[\frac{1}{n}\log_2[q_2(1-p)^{-1}] - \frac{1}{n} + \log_2 p^p(1-p)^{(1-p)}\Big] \\
&\quad - q_1 - q_2\Big\{\frac{1 + \log_2(1-p)}{n} - \log_2(1-p) - \frac{n_1}{n}\log_2[p/(1-p)]\Big\} \\
&\to q_1 - q_2\log_2 p^p(1-p)^{(1-p)} - q_1 - q_2\Big\{ -\log_2(1-p) - p\log_2[p/(1-p)]\Big\} \\
&= -q_2\log_2(1-p)^{(1-p)} - q_2(p-1)\log_2[(1-p)] = 0.
\end{aligned} \tag{S2.60}$$

It demonstrates that the long-time mutual information when using the trajectory entropy $\tilde{H}$ with the prefactor $1/n$ is zero, which is not proper as the two sets of time series are distinct. This analytical limit is confirmed by the numerical simulation shown in Fig. S10.

On the other hand, we used the trajectory entropy Eq. (S1.2) without the prefactor $1/n$. It is still not straightforward in the short-time regime for the example. In the long-time regime of the above example, the maximum mutual information converges to

$$I(R_{1:N}; M) \to -q_1 \log_2 q_1 - q_2 \log_2 q_2. \tag{S2.61}$$

The maximum is 1 bit, under the equal distribution of the two models $q_1 = q_2 = 1/2$. The result is the same if we considered two examples of the model with different p. The converged value 1 bit agrees with the maximum distinguishable information for the two examples. The temporal dynamics with various convergence rates correspond to how much distinguishable the two examples are. The analysis thus suggests that Eq. (S1.2) is the proper one to quantify the dMI.

3. *Numerical estimation on the dynamical mutual information*

According to the above analysis, the long-time mutual information from the entropy rate formula Eq. (S2.9) is zero, which can not give a reasonable converged value of the maximum mutual information. For two distinguishable ensembles of trajectories, the maximum mutual information should reach 1 bit in the long-time limit.

For the short-time regime, we numerically conduct the optimization on the mutual information. We can simulate multiple trajectories and conduct the ensemble average. It gives the full temporal dynamics before the long-time limit. We found that the peak mutual information with using $\tilde{H}$ is close to zero even for the two distinguishable models, as plotted in Fig. S10. Therefore, the trajectory entropy by $\tilde{H}$ with the prefactor $1/n$, i.e., Eq. (S2.9), is not suitable for estimating the maximum mutual information.

In order to quantify how much stimulus can be distinguished by the maximum mutual information, we thus need a different way to calculate the trajectory entropy and the mutual information. When we did not use the prefactor $1/n$ for the trajectory entropy, i.e. Eq. (S1.2) instead of Eq. (S2.9), the mutual information reaches 1 bit after certain amount of time, and the convergence rate depends on the difference between the models, as shown in Fig. S10. We have also checked that the convergence rate not only depends on the transition matrix of hidden states but also on the emission matrix.

Besides two different sets of time series, we also considered a scenario of four different sets of times series. They include the above two sets and another two sets generated by a hidden Markov model with $p = 0.1$, $\epsilon = 1$ and $p = 0.9$, $\epsilon = 1$ separately. Then, the four sets of trajectories are largely distinguishable, which can lead to maximum 2 bits of dMI over time. The calculation in Fig. S10 exactly shows that the trajectory entropy Eq. (S1.2) gives 2 bits of dMI in saturation, but Eq. (S2.9) generates 0 bit of information in the long time.

Overall, the dMI through trajectory entropy Eq. (S1.2) keeps increasing in the short-time regime, and converges to a finite value in the long-time limit. It agrees with the value of information to distinguish the different examples of the model. Instead, Eq. (S2.9) gives a decaying entropy rate and converges to 0 in the long-time limit.

III. SUPPLEMENTARY NOTE 3: QUANTIFYING THE DYNAMICAL MUTUAL INFORMATION BY THE TIME-INHOMOGENEOUS MARKOV MODEL

In this section, we provide the details to train the time-inhomogeneous Markov and calculate the dMI from the model. We further give the conventional model-wise statistics to show its difference to the trajectory-wise quantities.

A. Numerical implementations on the dynamical mutual information

1. *Training the time-inhomogeneous Markov model by experimental data*

We consider a discrete-time and discrete-state Markov model. The state transitions at next time point are only determined by the states' distribution at the current time point. When the transition probabilities are time-dependent, it is the time-inhomogeneous Markov model. The model has a set of time-dependent transition matrices $\{p(y_n|y_{n-1})\}_{n=1}^{N}$.

Given an ensemble of the trajectories, if we apply the Markov model, we can extract the time-dependent transition matrices by the following procedures. We first binned the observed data points into K discrete states. Second, for

each two consecutive time points, we counted the number of the data points with $y_n = j, y_{n-1} = i$. Then, we put the counts as the i-th row and j-th column element in the joint count matrix $c(y_n, y_{n-1})|_{t=n-1}$. The transition matrix was obtained by normalizing the joint count matrix by each column:

$$p(y_n|y_{n-1})|_{t=n-1} = \frac{c(y_n, y_{n-1})|_{t=n-1}}{c(y_{n-1})|_{t=n-1}}, \tag{S3.1}$$

where $c(y_{n-1})|_{t=n-1} = \sum_{y_n} c(y_n, y_{n-1})|_{t=n-1}$. The subscript specifies the time point of the transition probability. Then, we only need to repeatedly estimate the joint count matrix from the data at every time point.

2. *Quantification on the model performance*

When the number of trajectories is sufficient, the transition matrices can accurately represent all the possible trajectory configurations. We used the transition matrices to sample the same number of trajectories, and the heatmap of the trajectories has a similar dynamical pattern as the data in Fig. S2. We have also quantified the performance of training by the false k-nearest neighbor probabilities, the relative KL-divergence and the rescaled log-likelihood in Fig. 2. When the number of states increases, the measures show better model training and reaches saturation around 32 states. They demonstrate that the number of trajectories from the NFκB dataset is sufficient to train a time-inhomogeneous Markov model and that the model can reproduce the data well.

We remark that the rescaled log-likelihood tends to increase more versus the number of the states. This is caused by the rescaling procedure. Here, the trajectory probability also decays versus the number of time points, however, the decay is less significant than the hidden Markov model. The Markov model only has transition matrices, without the hidden states and the emission process. Thus, the major trajectory configurations are more likely to be captured, causing less decay of the average trajectory probability. Then, the rescaled log-likelihood mainly follows the trend of the rescaling factor, as given in Rescaled likelihood of Sect. II B 2. We kept using the rescaling procedure to be consistent with that of the hidden Markov model. Consequently, the rescaled log-likelihood is less informative than the false k-nearest neighbor probabilities and the KL-divergence for the Markov model.

Though the time-inhomogeneous Markov model can learn the dynamical patterns, it has assumed the Markov property of the trajectories, i.e., the data of each time point only depends on the earlier one time point. This may not be fully accurate, for example, the oscillations with various frequencies under the TNF stimulus indicate that there should be history-dependence for the trajectories. This history-dependence can not be captured by the time-inhomogeneous Markov model, but can be learned by the hidden Markov model.

3. *The suitability of the two models for representing various signaling dynamics*

In this manuscript, we have investigated the signaling molecules, NFκB, p53 and ERK (including ERK, p38, JNK). We used both the time-inhomogeneous Markov model and the hidden Markov model to learn the data, and studied how well these two models can reproduce the trajectories, as summarized below.

The performance of model training depends on the dynamical patterns of the trajectories. Specifically, for the NFκB or p53 (Fig. S6) dataset with unsynchronized dynamical patterns, the hidden Markov model provides more similar trajectories as the data than the Markov model does. For the ERK dataset (Fig. S7), the time-inhomogeneous Markov model outperforms the hidden Markov model to reproduce the trajectories, because the former can better recapture the synchronized patterns of the trajectory ensemble.

Indeed, the ERK dataset (Fig. S7) contains two time-windows, before and after the stimulus, which have dramatically different signaling activities. Thus, if using the hidden Markov model, two hidden Markov models may be required separately for the two time-windows. To validate it, we perform the model training for the two time-windows and found that indeed the models can generate similar trajectories to the data, as shown in Fig. S7. Therefore, the hidden Markov model should be able to learn the signaling dynamics under fixed environmental conditions, and multiple hidden Markov models are required when the environment is changed and causes a perturbation on the signaling system.

Besides, the proper choice on the model depends on the number of trajectories. In general, for the conditions with trajectories less than 300 and synchronized dynamical patterns, the Markov model may work better to represent the trajectories. When the number of trajectories is sufficient, e.g., more than 500, the hidden Markov model may be better. Thus, these two models have their own advantages to learn the trajectories with certain patterns, and should be chosen for specific purpose.

The measures on training performance developed above can help select the model. The choice of the model also depends on the further analysis. For example, if one needs to evaluate the maximum mutual information, which is

the major task in this manuscript, the time-inhomogeneous Markov model is sensitive to overfitting, because it has abundant parameters in the set of transition matrices. Consequently, there is a gap between the proper number of states that can train a high-performance model and avoid overfitting (Fig. S5). The hidden Markov model is more proper to avoid the overfitting problem on calculating the maximum mutual information (cf. Sect. II B 3). Overall, we provided two candidate models to learn stochastic trajectories and the criteria for the model selection.

4. Estimation on the dynamical mutual information

Once we have inferred the transition matrices $p(y_n|y_{n-1})|_{t=n-1}$ from the data, we can use Eq. (S1.2) to estimate the trajectory entropy for each trajectory, i.e., $H(y_{1:n}) \doteq -\log_2 p(y_{1:n})$. The trajectory probability $p(y_{1:n})$ is obtained by taking the transition probabilities from the transition matrices for observing the trajectory and multiplying the probabilities at all the time points. This algorithm does not sum up the probabilities for all the trajectory configurations by multiplying the matrices. Instead, the trajectory probability is calculated for each trajectory. The algorithm belongs to the trajectory-wise, different from the model-wise. Similar as the hidden Markov model, the trajectory probability $p(y_{1:n})$ also decays with the number of time points, because only one element of the transition matrix is taken at each time step.

The trajectory probability can be recognized as a posterior probability of the trajectory, based on the inferred transition matrices. When the data is sufficient to train a time-inhomogeneous Markov model, the underlying dynamics for each condition can be well represented by the set of transition matrices. Then, each trajectory has its probability under the trained model. The trajectory entropy is obtained by Eq. (S1.2), and the dMI can be calculated similarly as that of the hidden Markov model.

Though the time-inhomogeneous Markov model can be well trained and reproduce the trajectories for many conditions when the number of states is sufficient, its overfitting is more sensitively dependent on the number of states. The model has a set of transition matrices, where each needs to be trained by two consecutive time points of the data. The number of parameters is approximately $N/2$-times more than the hidden Markov model, where N is the number of time points. Therefore, the time-inhomogeneous Markov model is more sensitive to the overfitting issue.

To quantify the effect of the overfitting on the dMI, we conducted the same procedure of equally partitioning trajectories as that of the hidden Markov model, cf. Quantifying overfitting by the equal-partition within each condition of Sect. II B 3. The maximum mutual information with using the two subsets for each condition was calculated. In Fig. S5 and Fig. S8, the maximum mutual information becomes nonzero even when the number of states is 4. Thus, the time-inhomogeneous Markov model reaches the overfitting regime under much less number of states than the hidden Markov model does. On the other hand, to train a good Markov model requires at least 16 states, as indicated in Fig. 2. Therefore, there is a gap in the number of states required to train a good time-inhomogeneous Markov model and avoid the overfitting issue simultaneously.

Considering above, we chose the hidden Markov model to quantify the maximum mutual information for the signaling responses. The existence of the regime with high-performance model training and no serious overfitting issue gives the unique advantage of the hidden Markov model for the problem under study. The time-inhomogeneous Markov model can be adopted for the purpose of representing certain type of trajectories, such as the ERK dataset. However, one needs to keep in mind that it may have serious overfitting issue.

B. The model-wise mutual information from the time-inhomogeneous Markov model

In this subsection, we provide the formulation on calculating the model-wise maximum mutual information. For the Markov model, we can sum over all the possible transition probability for the trajectories to get a model-wise trajectory entropy. The total model-wise trajectory entropy of the time-homogeneous Markov model is rigorously given in [20]. Though the procedure is similar, for the purpose of completeness, we repeated the derivation for the time-inhomogeneous Markov model.

We started from the general formula of the model-wise trajectory given by Eq. (S2.18). With the trained time-inhomogeneous model, the trajectory ensemble can be recovered by a series of time-dependent transition matrices $\{p(y_n|y_{n-1})\}_{n=1}^N$ inferred from the data under each stimulus. Then, the probability of trajectory ensemble is given by the product of the transition matrices:

$$p[y_{1:N}] = \Pi_{n=1}^N p(y_n|y_{n-1})|_{t=n-1} p(y_0)|_{t=0}. \tag{S3.2}$$

Since these transition matrices obey: for each $n \in [1, N]$,

$$\sum_{y_n} p(y_n|y_{n-1})|_{t=n-1} = 1, \tag{S3.3}$$

$$\sum_{y_{n-1}} p(y_n|y_{n-1})|_{t=n-1} p(y_{n-1})|_{t=n-1} = p(y_n)|_{t=n}, \tag{S3.4}$$

the model-wise trajectory entropy can be simplified as:

$$\begin{aligned}
\hat{H}[y_{1:N}] &= -\sum_{\mathbf{y}} \Pi_{n=1}^{N} p(y_n|y_{n-1})|_{t=n-1} p(y_0)|_{t=0} \log_2 \Pi_{n=1}^{N} p(y_n|y_{n-1})|_{t=n-1} p(y_0)|_{t=0} \\
&= -\sum_{y_0} p(y_0)|_{t=0} \log_2 p(y_0)|_{t=0} - \sum_{y_1, y_0} p(y_1|y_0)|_{t=0} p(y_0)|_{t=0} \log_2 p(y_1|y_0)|_{t=0} \\
&\quad \cdots - \sum_{y_N, y_{N-1}} p(y_N|y_{N-1})|_{t=N-1} p(y_{N-1})|_{t=N-1} \log_2 p(y_N|y_{N-1})|_{t=N-1}, \\
&= -\sum_{y_0} p(y_0)|_{t=0} \log_2 p(y_0)|_{t=0} - \sum_{n=1}^{N} \sum_{y_n, y_{n-1}} p(y_n|y_{n-1})|_{t=n-1} p(y_{n-1})|_{t=n-1} \log_2 p(y_n|y_{n-1})|_{t=n-1}.
\end{aligned} \tag{S3.5}$$

Here, the prefactor is given by the transition probabilities. It is different from the trajectory entropy Eq. (S1.2) for each trajectory. From the trajectory-wise view, we need to consider each single trajectory, and the entropy is the trajectory entropy [3]. On the other hand, the model-wise trajectory entropy counts all the possible trajectory configurations in the probability sense, without focusing on every trajectory. It sums up all the transition probabilities of the trajectory ensemble as a whole, and thus uses the transition probability as the prefactor of the logarithm function.

Given the model-wise trajectory entropy under one stimulus, we can get the unconditional and conditional model-wise trajectory entropy similarly as in Sect. I B. The mutual information is given by Eq. (S1.8), i.e., $\hat{I}(R_{1:n}; S) = \hat{H}(R_{1:n}) - \hat{H}(R_{1:n}|S)$, where now the entropy $\hat{H}(R_{1:n})$ and $\hat{H}(R_{1:n}|S)$ are the model-wise.

The initial distribution does not generate mutual information, since all the conditions are the same. Then, the model-wise mutual information is:

$$\hat{I}(R_{1:n}; S) = \sum_{n=1}^{N} \sum_{i} q_i \sum_{y_n} \sum_{y_{n-1}} p(y_n, y_{n-1}|x) \Big[\log_2 \frac{p(y_n, y_{n-1}|S=i)}{p(y_{n-1}|S=i)} - \log_2 \frac{\sum_k q_k p(y_n, y_{n-1}|S=k)}{\sum_k q_k p(y_{n-1}|S=k)} \Big], \tag{S3.6}$$

where we have neglected the subscript of specifying the time point for the probability. With the inferred joint probability matrices, every term in this formula can be evaluated. The maximum mutual information $\hat{I}_{max}(R_{1:n}; S)$ follows by maximizing the mutual information $\hat{I}(R_{1:n}; S)$ with respect to the stimuli's distribution $\{q_i\}$. Note that the model-wise maximum mutual information from the time-inhomogeneous Markov model also suffers from the overfitting problem.

IV. SUPPLEMENTARY NOTE 4: COMPARISON WITH THE PREVIOUS METHODS OF CALCULATING THE MUTUAL INFORMATION

In this section, we briefly compare the methods of calculating the mutual information, including the previous classical methods in the field [5, 7, 8].

A. The time-point method

In the pioneering work of applying information theory to single-cell biochemical signaling process [7], they estimated the mutual information by using one time-point data. When having multiple time-point measurements, one can repeat the method for each time point and get the point-by-point maximum mutual information.

We have applied the method to our dataset, and estimated the maximum mutual information from each time-point measurement. In Fig. 3, the maximum mutual information by the time-point method peaks around 0.5 hour, and starts to decrease after that time point, revealing that the stimuli are less distinguishable at other time points. In Fig. S16, for the case of the TNF-Poly(I:C) pair (green), the mutual information from the time-point method becomes zero after the first few time points, which loses the information from those early time points. Thus, the

method mainly captures the instantaneous information transmission from one time-point response. However, in the biochemical signaling process, the time course of response affects the signaling transduction and the downstream gene expression [21, 22].

B. The vector method

The time-point method has been generalized to include the time series [5]. The method, which is termed as the vector method here, treats the time course of each single-cell response as a multi-dimensional vector, and estimates the probability of each vector by the k-nearest neighbor density estimator [23].

We applied the method to our dataset, and plotted the maximum mutual information in Fig. 3. At each time point, the maximum mutual information was obtained by using the vector of time series up to that time point. The maximum mutual information first keeps increasing with the number of time point, as more information is included with more measurements. When the number of time points reaches around 10, the maximum mutual information starts to decrease, which also happens for the mutual information under the pairwise stimuli in Fig. S16. The reason is that when the dimension of the time-series vector increases, the data vectors become sparse in the high dimensional space. Then, without having prohibitive numbers of data vectors, one could not get an accurate estimation on the probability of the high-dimensional data vector. Given the current measurements on single-cell NFκB signaling responses, this method becomes inaccurate with more than around 10 time points.

To avoid this technical constrain, we have also applied the vector method under another scheme, i.e. scheme 2 with using 5 neighbor data points at each time point in Figs. S16, S20. Then, the result is similar to that from the time-point method, except that the mutual information from the vector method is bit higher, because more data points were employed at each time point. Besides, the grid of sampling points affects the mutual information estimation from the vector method, e.g., the grid in Fig. S19 of [5] can give higher mutual information. Here, we have chosen a specific way, i.e., using consecutive points, to estimate the information accumulation in real time.

The vector method is not altered by the ordering of time points. We demonstrated it by randomly permutating the data points first (cf. **Implementation on permutating the data** of Sect. II B 3 for the detailed procedures of permutation). We then used the vector method to estimate the maximum mutual information from the permutated time series. In Fig. 3, the permutation does not alter the result calculated from this method. Thus, the method does not reflect the information from the dynamical patterns with the data aligned in certain order.

C. The decoding-based method

The decoding-based method was recently proposed [8, 24]. In brief, they used a around 70% data of various stimulus conditions to train a classifier, and used the classifier to compute the confusion matrix among stimuli for the remaining 30% data. The confusion matrix was then used to infer a lower bound on the mutual information.

We have applied the decoding-based method (https://github.com/swainlab/mi-by-decoding) to the NFκB dataset, and noticed the following properties.

1. The maximum mutual information with using all the stimuli has a sudden information change at certain time point depending on the number of principle components (PCs) (Fig. S14), and saturates to relatively constant afterwards. The result is similar when using the first few PCs, once the number of PCs is sufficient (Fig. S14), e.g., more than 5.

2. The result, especially the saturated mutual information value, does not sensitively depend on the ordering of the time points (Figs. 3, S14). After random permutation, the data under various stimulus conditions can still have distinct features. For example, if considering the conditions of control (no stimulus) and LPS, their responses can still be distinguished, because the former has all responses low and the later has many high response values. The conditions of LPS and TNF also have distinct number of data points with values over 4. Therefore, even after random permutation, the maximum mutual information should still accumulate above 1 bit, as shown by both dMI and the decoding-based method (Fig. 3). The zero mutual information for the random permutated trajectories of their yeast dataset [8] may be caused by that their dataset has less diverse dynamical features as the current one.

3. For the pairwise stimuli in Fig. S16, the mutual information for TNF-LPS pair is overall lower than that for TNF-Poly(I:C) pair. It is different from the extent of similarity between the trajectory ensembles in Fig. 4**b**, where the TNF-LPS pair has more distinct dynamical pattern. It may be due to that the oscillatory features are not fully decoded by the classifier.

These results from the decoding-based method might be caused by the following reasons.

- The method used the linear principle components of the time series for the classifier, which can perform less well to classify the oscillatory pattern.
- The classifier may have lower performance when the number of stimuli increases, and previously it was applied to around 5 stimulus conditions [8].
- When training the classifier, only the time series was used, without explicitly specifying the various dynamical features of the time series. Although it was reported that time series is enough for the classifier without using more features [8], adding the dynamical feature one-by-one was considered in their Fig. S16. Adding all the dynamical features coherently may help better estimate the information encoded in dynamics. On the other hand, the present method by the hidden Markov model automatically include all the dynamical features, because the trajectory probability was generated by the model with potentially representing the full dynamics.
- The decoding-based method used a uniform distribution of stimuli without the optimization on the distribution. However, the effect should be minor, because Fig. S18 in [8] shown that the result with using uniform distribution is similar to that with optimizing the distribution.

Overall, more detailed investigations are needed to apply the decoding-method to datasets with more stimuli and rich dynamical patterns.

D. The present method

The present method can learn the information encoded in the dynamical patterns of response trajectories cumulatively up to each time point. This type of information is particularly relevant as the downstream gene expression is affected by the time course of the signaling molecule [21, 22]. In Fig. 3, we have evaluated the maximum mutual information through the trajectory probability from the hidden Markov model. The dMI increases in the early time phase, around 2 hours, as the distinguishable dynamical patterns exist during the time window. Since the dynamical patterns of various stimuli still have certain degree of difference in the late time phase, the maximum mutual information does not drop and continues to accumulate, with a smaller rate than the early 2-hours.

The present method is dependent on the ordering of the time points. In Fig. 3, we have calculated the maximum mutual information with uniformly random permutation on the time points. It has lower maximum mutual information during most of the time phase compared with that of the original dataset, indicating that the genuine ordering of the data points leads to more distinguishable signaling responses. The random permutation also leads to higher maximum mutual information for the first few time points. It is caused by that the more diverse responses happen to be permuted in the early time phase, whereas in the original dataset the responses need a few time points to acquire the mutual information.

In addition, we have also calculated the mutual information between the two genotypes, WT and IκB-mutant, under the same stimulus (Fig. S21). The purpose for this calculation is to show that distinct signaling dynamical patterns of the two genotypes give information on their degree of responses' difference, when treated by the same stimuli. The present method reveals a gradual change on the information accumulation, corresponding to the cumulative dissimilarity of the trajectory ensembles. Specifically, the dynamical mutual information increases faster under the TNF or Poly(I:C) condition, and less under the LPS condition. They match with the extent of dissimilarity between the trajectory ensembles, which have the quite distinct dynamical patterns under the TNF or Poly(I:C) condition. Differently, the values of information from the three previous methods are underestimated, and are less than 0.3 bit for the two trajectory ensembles with largely distinguishable dynamical patterns, such as under TNF.

We remark that the values of the maximum mutual information between the methods are altered by the noise. Each method counts different degrees of noise when calculating the mutual information. Though the present method is able to reveal the information embedded in the full dynamics, it is possible to learn the information from noise as well. We have discussed in detail on how to avoid the effect of noise within each stimulus in Sect. II B 2. Still, there is not a clear-cut on the mutual information from the real difference between various stimuli and that from noise.

Another method, statistical learning estimation of mutual information (SLEMI), was recently developed [25]. It overcomes the inaccurate density estimation of using the k-nearest neighbor method for the high-dimensional signaling-response data. However, the method relies on the assumption that the ratio of the probability of observing each trajectory between two stimuli is linear with respect to the trajectory responses. The linear approximation can be less accurate to distinguish the oscillatory trajectory, and thus the information encoded in the complex dynamical patterns may be underestimated. In their experimental dataset [25], there was also a lack of complex dynamical patterns in the trajectory ensembles, such as a lack of the oscillatory trajectories. The discrimination on various stimuli was not

investigated, nor was the information dynamics in the long time scale beyond 3 hours. As for the computational time, the present method requires the procedure of model training that takes additional computational time. However, the computational time is in the scale of 10 hours for the NFκB dataset on personal desktop with intel(R) core(tm) i7-8700 CPU @ 3.7GHz, which is affordable and can be accelerated with faster computational resources.

Besides, the Kalman filter holds a similarity to the hidden Markov model. It can be used to find the most likely hidden-state dynamics given the observed time series, which is termed as filtering. Considering that it does not directly give a dynamical model to further quantify the trajectory probability and the trajectory entropy, we chose the hidden Markov model in this manuscript.

In addition, the information-theoretic framework [26] obtained an approximated analytical expression for the channel capacity of cell populations by regressing to the single-cell level. It used a theoretical model of signaling network. Differently, here we analyze the experimental data and provide a general framework to quantify the information transmission via the measured single-cell signaling responses.

1. *Biological insights from the present method*

- The information for stimulus distinction may be decoded by responsive gene expression

  The present method shows various temporal modes of information accumulation for pairwise stimuli (Figs. 4, S15), indicating a temporal order of distinguishing each stimulus pair.

  To investigate how cells potentially use the information of NFκB for downstream gene expression, we analyzed the data of NFκB responsive genes [27], and compared the difference of gene expression with the dMI for each stimulus pair. We used the measured NFκB-responsive gene expression data (cluster B,C in Fig. 5 of [27]) at three time points (1, 3, 8 hours) in the same cell type and experimental condition, under the treatment of TNF, Poly(I:C), or CpG separately. We first calculated the correlation between the dMI values and the absolute difference of gene expression fold change between the pairwise stimuli, at the time points 0, 1, 3, 8 hours. From the histogram of this correlation in Fig. S19, a large proportion of NFκB-responsive genes track the signaling information of NFκB for the stimulus discrimination. More time points of expression measurements may improve the robustness of the correlation.

  We further chose representative genes whose expression patterns track the information accumulation, as shown in Fig. S19. For example, the protease inhibitor Serpina3f [28] is induced when macrophages sense the presence of bacteria (LPS) locking up infected tissues to prevent bacterial spread, but not when they sense the presence of TNF which derives from neighboring cells. The distinction is sensed early and is sustained. Further, Lad1, which allows innate immune cells to be recruited to sites of infections [29], quickly responds to sources of TNF, but will not respond quickly when these cells sense poly(I:C) as a result of being infected themselves. In contrast, Faah, which dampens excessive inflammation by removing inflammatory fatty acids [30], is induced by all pro-inflammatory stimuli. However, if the infectious threat persists, Faah is downregulated in response to the bacterial stimulus (CpG), to potentiate inflammation-mediated cellular immunity, while an effective anti-viral response to poly(I:C) does not rely on an inflammatory response but on the interferon program [31].

  While these observations are promising, we would like to point out the following limitations and challenges when relating gene expression to signaling responses. First, the mutual information quantifies the discrimination among stimuli based on the single-cell data, whereas the gene expression data is based on a population level bulk measurement. Future studies may employ knockin fluorescent reporters to report single cell gene expression dynamics following stimulation. Second, while these genes were selected to be NFκB target genes we cannot exclude the involvement of other factors that may be activated by the stimuli and may play a role in gene expression control, thus confounding the analysis. Future studies may address this limitation by using experimental approaches that avoid co-activating other pathways, such as optogenetic control of transcription factors [22]. Nevertheless, the data presented here may be a motivation for further exploration of this topic.

- Validating the temporal phases of dMI by using a mathematical model of NFκB signaling network

  The dMI enables investigation of the trajectory of information accumulation. As different components of the molecular network that generates the signaling dynamics, such as molecular mechanisms, circuit motifs, feedback or feedforward loops, operate at different timescales, they may be responsible for different phases of information accumulation. In Fig. 5, we have estimated the maximum mutual information for IκB-mutant in Fig. 5, where the negative feedback to NFκB was reduced. Without the feedback, the signaling responses lose dynamical patterns such as oscillations for certain stimulus, which decreases the accumulation of mutual information within the $1-2$hr phase.

To further validate the hypothesis of the temporal phases of dMI in Fig. 5, we used an experimentally validated mathematical model of the NFκB signaling network [32] to simulate NFκB trajectories. To account for the cell-to-cell heterogeneity, parameters were distributed as described in [33, 34]. We generated 1000 trajectories of virtual cell-to-cell variable NFκB responses. We made three perturbations: 1) reduced stimulus availability to diminish the first phase of information accumulation, 2) reduced the negative feedback parameter to affect the second phase (akin to the experimental work), and 3) enhanced the ligand-receptor degradation rate to diminish the third phase. The trajectories (with normalized maximum response to 10) under each stimulus condition is plotted as a heatmap in Fig. S22. We then trained a hidden Markov model for each condition, and calculated the dMI by considering the following pairwise conditions. The dMI shows distinct temporal profiles corresponding to the difference of specific dynamical features, as discussed below.

First, for the early activation phase, we considered 10ng/ml LPS stimulation with normal or 500-fold reduced ligand availability. This comparison models the different ligand-receptor interactions of the ligand LPS and receptors, which gives distinct early activation of responses. Correspondingly, the dMI has an increase in the first few minutes.

Second, for the intermediate oscillatory phase, we considered 10ng/ml TNF to the WT model or a model in which NFκB-responsive transcription of IκBα is 5-fold reduced. The latter case has less oscillatory responses. The dMI increases at the time scale of the first oscillation, around 1 hour, indicating that distinct oscillatory patterns may provide information in the intermediate temporal phase.

Third, for the late sustained phase, we considered 10ng/ml LPS in a WT model or one in which the degradation rate of the activated TLR4-LPS is increased by 100-fold. Accordingly, the dMI increases relatively late, around 2 hours, which mainly comes from the difference of the responses' integral and duration. The late decrease of dMI is caused by that the two trajectory ensembles gradually become similar due to the response decay.

We note that there may be no clear-cut separation on the source of information from different dynamical features. Specifically, the first pairwise conditions have differences in the sustained features, such that the dMI increases again in the late time window. The dMI for the third pairwise conditions can be attributed to both oscillation and duration.

Overall, the analysis reveals that the temporal phases of information accumulation can come from stimulus-specific dynamic features that are deployed at specific time scales, because they are generated by circuit motifs or molecular mechanisms that operate at specific time scales. These initial findings with the mathematical model warrant future experimental confirmation.

V. SUPPLEMENTARY NOTE 5: EXPERIMENTAL INFORMATION

A. Experimental information for the NFκB dataset

- Mouse models.

The mVenus-RelA endogenously-tagged mouse line (C57BL/6J background) was generated by Ingenious Targeting Laboratory. A donor sequence encoding the monomeric variant of the Venus fluorescent protein [35] joined by a short flexible linker sequence directly upstream of the start codon of the murine RelA locus was implanted to yield heterozygous mice, via homologous recombination. These mice were then bred with a mouse line constitutively expressing the Flp recombinase, to remove the Neo resistance marker included in the homologous donor sequence. We then back-crossed the resultant mice with wild-type C57BL/6J mice to remove the Flp background and generate homozygous mVenus-tagged mice. For the IκB-mutant, we crossed mVenus-RelA mice with IκB$\alpha^{M/M}$ mouse line (also C57BL/6J background; kind gift from Paul Chiao) that harbors mutated κB sites in the enhancers of IκBα promoter [36].

All mice strains have either sex, and are 8-24 weeks old. Mice were housed in an accredited UCLA Vivarium at ambient temperature and humidity with a 7am-7pm light/dark cycle.

- Macrophage cell culture.

Bone Marrow-Derived Macrophages (BMDMs) were prepared by culturing bone marrow monocytes from femurs of 8-24 weeks old WT mice in L929-conditioned medium using standard methods [33, 37]. BMDMs were re-plated in imaging dishes on day 4, then were stimulated on either day 7 or day 8 following treatment.

- Macrophage stimulation conditions.

BMDMs were stimulated with the toll-like receptor 4 (TLR4) agonist lipopolysaccharide (LPS) (Sigma Aldrich), TLR3 agonist polyinosine-polycytidylic acid (Poly(I:C)) (Invivogen), TLR9 agonist CpG B ODN (invivogen), TLR2 agonist Pam3CSK4 (invivogen), and TNF (Bio-techne).

- Live-cell imaging.

  BMDMs were re-plated on day 4 at 20,000 or 15,000/cm^2 in an 8-well ibidi SlideTek chamber, for imaging at an appropriate density (approximately 60,000/cm^2) on day 7 or day 8. 2 hours prior to stimulation, a solution of 5 ng/mL Hoechst 33342 is added to the BMDM culture media. After 5 minutes of the start of imaging, conditioned culture media containing stimulus (LPS, Poly(I:C), TNF, CpG, or Pam3CSK4) was injected into the chamber in situ. Cells were imaged at 5-minute intervals on a Zeiss AxioObserver platform with live-cell incubation, using epifluorescent excitation from a Sutter Lambda XL light source. Images were recorded on a Hamamatsu Orca Flash 2.0 CCD camera for 12 hours.

- Image analysis and processing.

  Microscopy time-lapse images were exported for single-cell tracking and measurement in MATLAB R2018a. The tracking routines followed those used in the earlier work [5]. Briefly, cells were identified using DIC images, then segmented, guided by markers from the Hoechst image. Segmented cells were linked into trajectories across successive images, then nuclear and cytoplasmic boundaries were saved and used to define measurement regions in other fluorescent channels, including mVenus-NFκB. Nuclear NFκB levels were quantified on a per-cell basis, normalized to image background levels, then were baseline-subtracted. Mitotic cells, as well as cells that drifted out of the field of view, were excluded from analysis. The toolboxes used for this analysis are available at GitHub (https://github.com/Adewunmi91/MACKtrack).

B. Tables of the datasets

The statistics of the NFκB dataset were plotted in Fig. S1. There are 13 different stimulus conditions. Four replicates for WT were included for the convenience of comparing the identical condition, and they does not affect the mutual information estimation as they have the identical stimulus and concentration. The concentrations of stimuli in Table S3 were used as they induce representative dynamical patterns of NFκB responses.

Besides the NFκB dataset, we have used the dataset of p53 [38] and the dataset of p38, JNK, ERK [39] from the previously published work. They were mainly used to demonstrate the feasibility of the time-inhomogeneous Markov model and the hidden Markov model to learn the signaling dynamics, and were not employed for mutual information calculation from the hidden Markov model. We listed the experimental information about these datasets in Tables S3 S4 S5.

[1] Cover, T. M. & Thomas, J. A. *Elements of Information Theory* (Wiley, New York, 1991).

[2] Gardiner, C. W. *Handbook of Stochastic Methods* (Springer-Verlag, Berlin, 2004), 3rd edn.

[3] Seifert, U. Entropy production along a stochastic trajectory and an integral fluctuation theorem. *Phys. Rev. Lett.* **95**, 040602 (2005).

[4] Seifert, U. Stochastic thermodynamics, fluctuation theorems and molecular machines. *Rep. Prog. Phys.* **75**, 126001 (2012).

[5] Selimkhanov, J. *et al.* Accurate information transmission through dynamic biochemical signaling networks. *Science* **346**, 1370–1373 (2014).

[6] Baum, L. E. & Petrie, T. Statistical inference for probabilistic functions of finite state markov chains. *Ann. Math. Stat.* **37**, 1554–1563 (1966).

[7] Cheong, R., Rhee, A., Wang, C. J., Nemenman, I. & Levchenko, A. Information transduction capacity of noisy biochemical signaling networks. *Science* **334**, 354–358 (2011).

[8] Granados, A. A. *et al.* Distributed and dynamic intracellular organization of extracellular information. *Proc. Natl. Acad. Sci. USA* **115**, 6088–6093 (2018).

[9] Griliches, Z. *et al.* *Handbook of Econometrics* (Elsevier, 1983).

[10] Jelinek, F., Bahl, L. & Mercer, R. Design of a linguistic statistical decoder for the recognition of continuous speech. *IEEE Trans. Inform. Theory* **21**, 250–256 (1975).

[11] Bahl, L., Cocke, J., Jelinek, F. & Raviv, J. Optimal decoding of linear codes for minimizing symbol error rate (corresp.). *IEEE Trans. Inform. Theory* **20**, 284–287 (1974).

[12] Ernst, J. *et al.* Mapping and analysis of chromatin state dynamics in nine human cell types. *Nature* **473**, 43 (2011).

[13] Ye, F. X.-F., Ma, Y.-a. & Qian, H. Estimate exponential memory decay in hidden markov model and its applications. *arXiv:1710.06078* (2017).

[14] Jacquet, P., Seroussi, G. & Szpankowski, W. On the entropy of a hidden markov process. *Theor. Comput. Sci.* **395**, 203–219 (2008).
[15] Roldán, E. & Parrondo, J. M. R. Entropy production and kullback-leibler divergence between stationary trajectories of discrete systems. *Phys. Rev. E* **85**, 031129 (2012).
[16] Collet, P., Leonardi, F. *et al.* Loss of memory of hidden markov models and lyapunov exponents. *Ann. Appl. Probab.* **24**, 422–446 (2014).
[17] Viterbi, A. Error bounds for convolutional codes and an asymptotically optimum decoding algorithm. *IEEE Trans. Inf. Theory* **13**, 260–269 (1967).
[18] Kullback, S. & Leibler, R. A. On information and sufficiency. *Ann. Math. Stat.* **22**, 79–86 (1951).
[19] Schulman, L. *Techniques and Applications of Path Integration*, vol. 140 (Wiley, New York, 1981).
[20] Pressé, S., Ghosh, K., Lee, J. & Dill, K. A. Principles of maximum entropy and maximum caliber in statistical physics. *Rev. Mod. Phys.* **85**, 1115–1141 (2013).
[21] Sen, S., Cheng, Z., Sheu, K. M., Chen, Y. H. & Hoffmann, A. Gene regulatory strategies that decode the duration of nfκb dynamics contribute to lps-versus tnf-specific gene expression. *Cell Syst.* **10**, 169–182 (2020).
[22] Chen, S. Y. *et al.* Optogenetic control reveals differential promoter interpretation of transcription factor nuclear translocation dynamics. *Cell Syst.* (2020).
[23] Voliotis, M., Perrett, R. M., McWilliams, C., McArdle, C. A. & Bowsher, C. G. Information transfer by leaky, heterogeneous, protein kinase signaling systems. *Proc. Natl. Acad. Sci. USA* **111**, E326–E333 (2014).
[24] Cepeda-Humerez, S. A., Ruess, J. & Tkačik, G. Estimating information in time-varying signals. *PLoS Comput. Biol.* **15**, e1007290 (2019).
[25] Jetka, T., Nienałtowski, K., Winarski, T., Błoński, S. & Komorowski, M. Information-theoretic analysis of multivariate single-cell signaling responses. *PLoS Comp. Biol.* **15**, e1007132 (2019).
[26] Jetka, T., Nienałtowski, K., Filippi, S., Stumpf, M. P. & Komorowski, M. An information-theoretic framework for deciphering pleiotropic and noisy biochemical signaling. *Nat. Commun.* **9**, 1–9 (2018).
[27] Cheng, C. S. *et al.* Iterative modeling reveals evidence of sequential transcriptional control mechanisms. *Cell systems* **4**, 330–343 (2017).
[28] Heit, C. *et al.* Update of the human and mouse serpingene superfamily. *Hum. Genomics* **7**, 22 (2013).
[29] Ochs, H. D., Smith, C. E. & Puck, J. M. *Primary immunodeficiency diseases: a molecular & cellular approach* (Oxford University Press, 2006).
[30] Sałaga, M., Sobczak, M. & Fichna, J. Inhibition of fatty acid amide hydrolase (faah) as a novel therapeutic strategy in the treatment of pain and inflammatory diseases in the gastrointestinal tract. *Eur. J. Pharm. Sci.* **52**, 173–179 (2014).
[31] Sheu, K. M., Luecke, S. & Hoffmann, A. Stimulus-specificity in the responses of immune sentinel cells. *Curr. Opin. Syst. Biol.* **18**, 53–61 (2019).
[32] Taylor, B., Adelaja, A., Liu, Y., Luecke, S. & Hoffmann, A. Identification and physiological significance of temporal nfκb signaling codewords deployed by macrophages to classify immune threats. *bioRxiv* (2020).
[33] Cheng, Z., Taylor, B., Ourthiague, D. R. & Hoffmann, A. Distinct single-cell signaling characteristics are conferred by the myd88 and trif pathways during tlr4 activation. *Sci. Signal.* **8**, ra69–ra69 (2015).
[34] Maity, A. & Wollman, R. Information transmission from nfkb signaling dynamics to gene expression. *PLoS Comput. Biol.* **16**, e1008011 (2020).
[35] Koushik, S. V., Chen, H., Thaler, C., Puhl III, H. L. & Vogel, S. S. Cerulean, venus, and venusy67c fret reference standards. *Biophys. J.* **91**, L99–L101 (2006).
[36] Peng, B. *et al.* Defective feedback regulation of nf-κb underlies sjögren's syndrome in mice with mutated κb enhancers of the iκbα promoter. *Proc. Natl. Acad. Sci. USA* **107**, 15193–15198 (2010).
[37] Takeshita, S., Kaji, K. & Kudo, A. Identification and characterization of the new osteoclast progenitor with macrophage phenotypes being able to differentiate into mature osteoclasts. *J. Bone Miner. Res.* **15**, 1477–1488 (2000).
[38] Chen, S.-h., Forrester, W. & Lahav, G. Schedule-dependent interaction between anticancer treatments. *Science* **351**, 1204–1208 (2016).
[39] Regot, S., Hughey, J. J., Bajar, B. T., Carrasco, S. & Covert, M. W. High-sensitivity measurements of multiple kinase activities in live single cells. *Cell* **157**, 1724–1734 (2014).

VI. SUPPLEMENTARY FIGURES

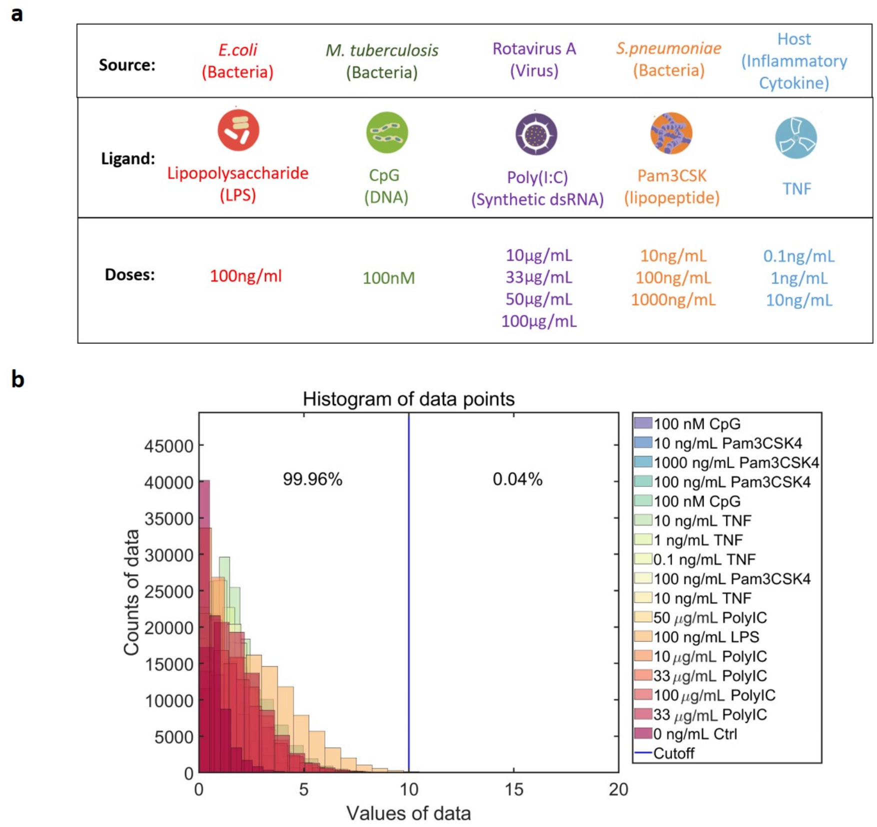

FIG. S1. (Color online) The experimental information and the statistics of NFκB dataset, as supplemental to Fig. 2. (**a**) All the stimulus conditions used for the NFκB signaling responses. The sources and concentrations are listed. There are in total 13 different stimulus conditions. (**b**) The statistical distribution of all the measured data points, where the colors specify each condition. Four replicates were included for the convenience of comparing the identical stimulus. As the replicates have the identical stimulus and concentration, they do not affect the mutual information calculation. For the purpose of model training, we set the value over 10 to be 10 uniformly for all the stimuli. Since less than 0.1% of data points have over 10 values, this cutoff does not dramatically alter the subsequent modeling procedures. Source data are provided as a Source Data file.

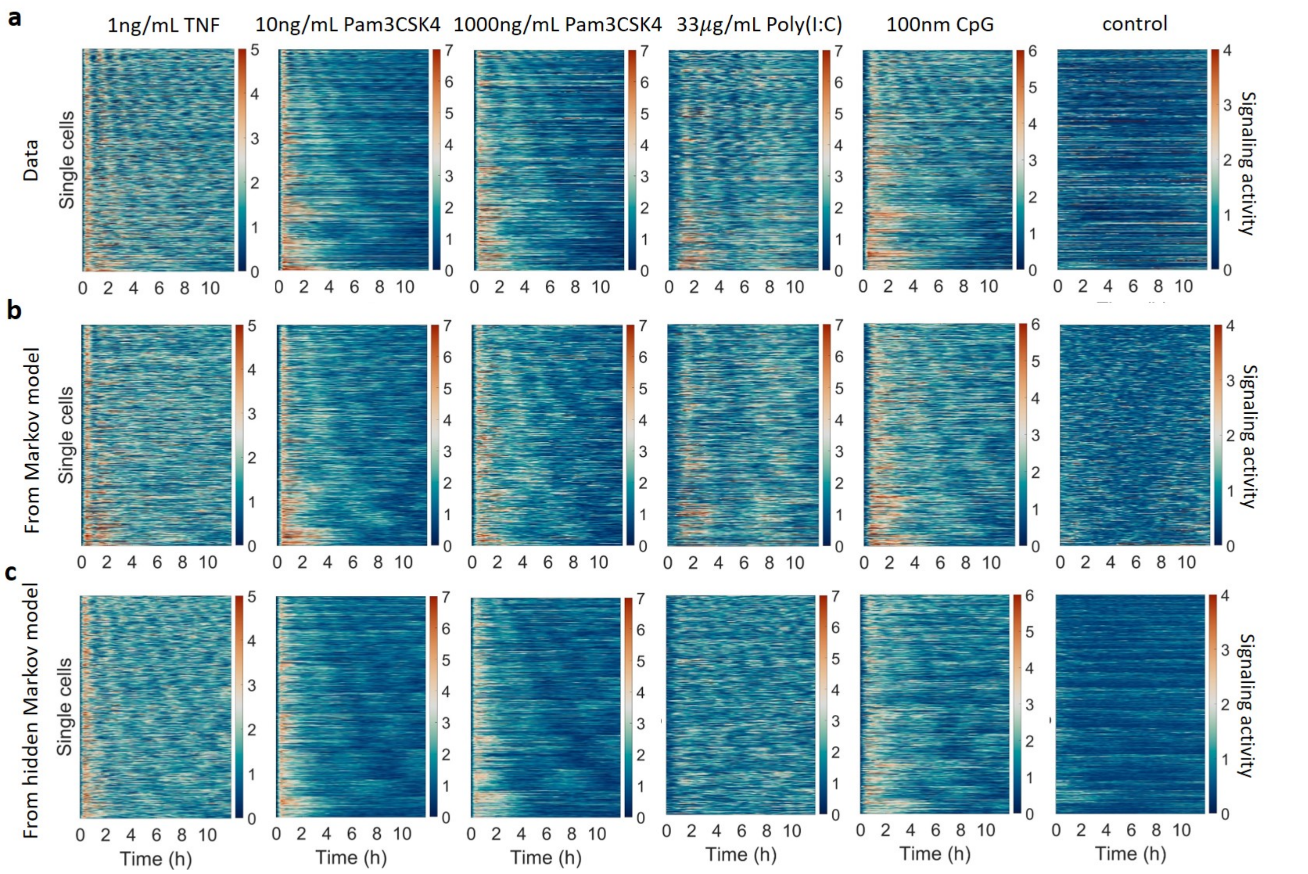

FIG. S2. (Color online) Single-cell signaling dynamics can be learned by a time-homogeneous Markov model or a hidden Markov model (HMM), as supplemental to Fig. 2. (**a**) Examples of the experimental data. In the heatmaps, each row represents a single-cell response, and the column denote various time points, which were recorded every 5 minutes and 12 hours in total. The trajectories are ordered by their oscillatory frequencies from high to low. The colors illustrate the NFκB responses activity, and the range of the color is specified for each stimulus. The concentration and type of stimuli are listed above. (**b**,**c**) The heatmaps of the sampled trajectories from the time-inhomogeneous Markov model (**b**), and the hidden Markov model (**c**). Source data are provided as a Source Data file.

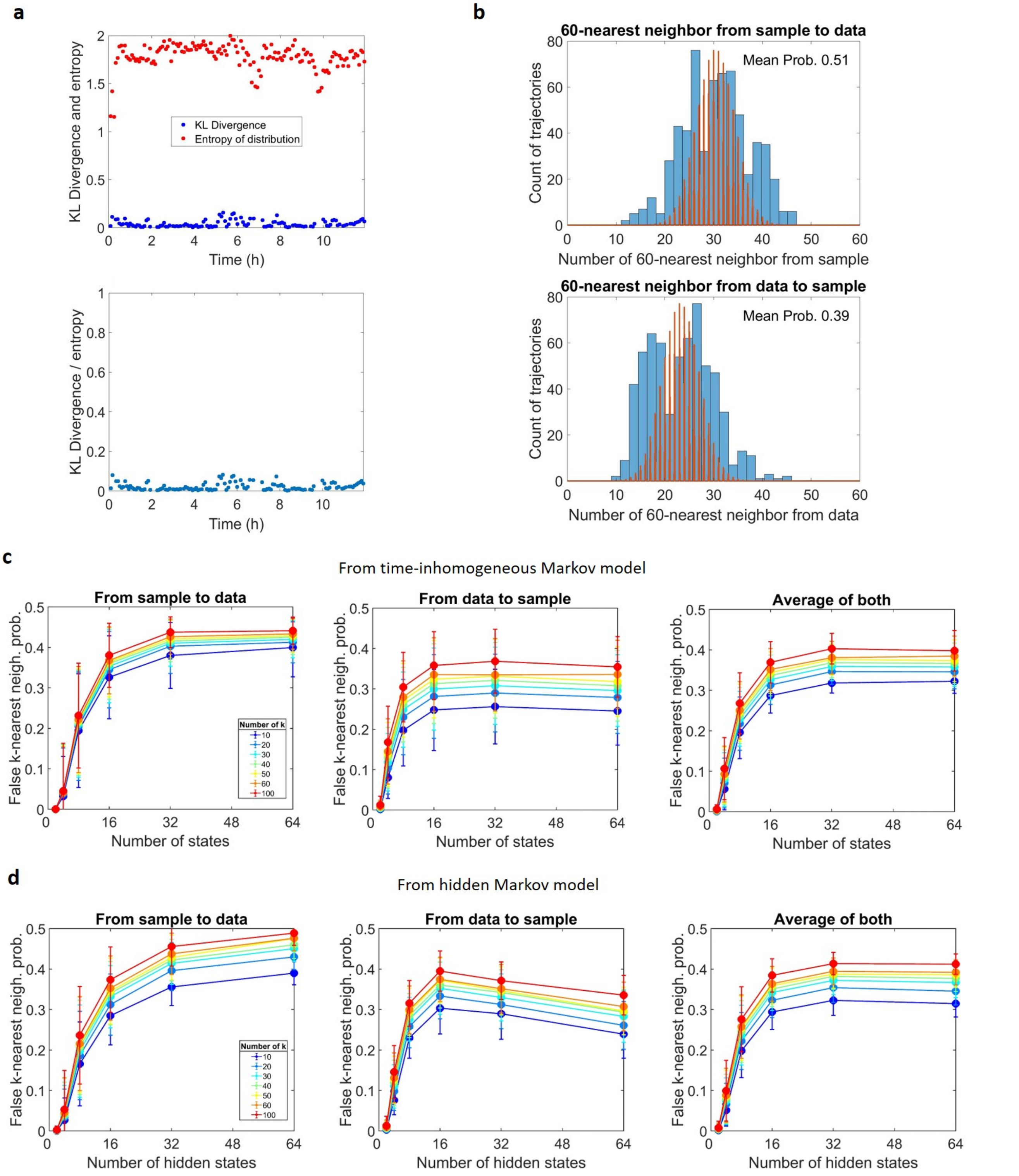

FIG. S3. (Color online) Details on the quantification of model performance for Fig. 2. (**a**) The KL-divergence between sampled trajectories and data, entropy of data, and the ratio of the two at every time point. (**b**) The histogram of the first 60-nearest neighbor from trajectories of data to a sampled trajectory (upper panel) and from sampled trajectories to a trajectory of data (lower panel). We fitted the histogram to a binomial distribution (red), and showed p of the binomial distribution as the mean probability of false k-nearest neighbor. The result for (**a**,**b**) is from the hidden Markov model under 100nM CpG. (**c**) The false k-nearest probability when using different number of states for the time-inhomogeneous Markov model. At each state's number, we plotted the mean and standard deviation as error bar for all the 13 stimulus conditions. For each stimulus, the probability is the average on all the trajectories. We also scanned the number of k in the k-nearest neighbor search as denoted by color, with $k = 10, 20, 30, 40, 50, 60, 100$. We chose $k = 60$, which is in a proper range of the number of neighbors. The three panels are from sample to data, from data to sample, and their average. We used the latter for Fig. 2. (**d**) The same as (**c**) for the hidden Markov model. Source data are provided as a Source Data file.

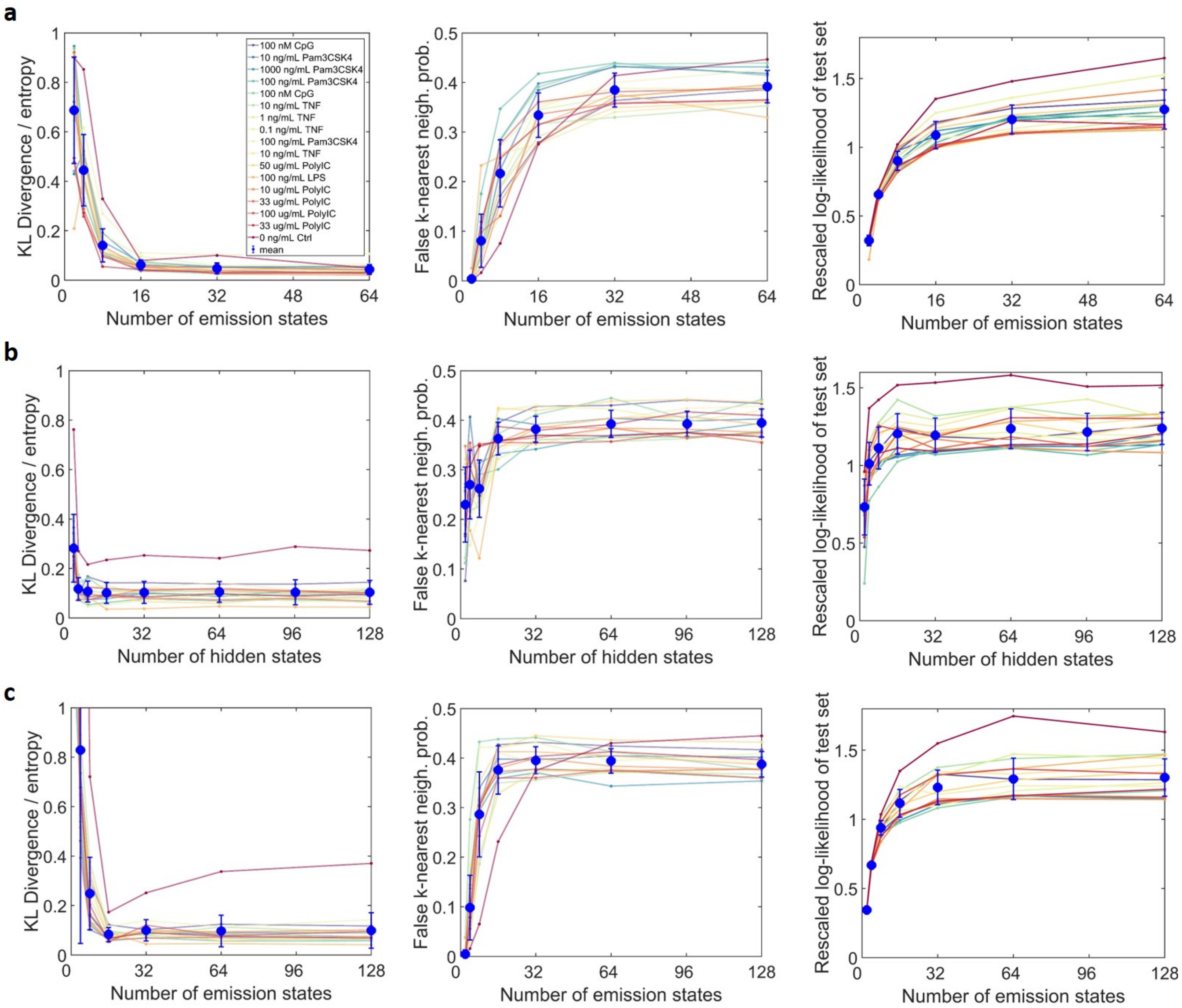

FIG. S4. (Color online) Detailed quantification on the training performance of the hidden Markov model, as supplemental to Fig. 2. (**a**) The three measures as in Fig. 2, with fixing the ratio between number of hidden states and emission states as 1, whereas the Fig. 2 has the ratio 2. (**b**) The three measures with independently scanning the number hidden states and fixing the number of emission states as 32. (**c**) The three measures with independently scanning the number emission states and fixing the number of hidden states as 64. In each panel, the error bar denotes the standard deviation on all the 13 stimulus conditions. The colors denote the same conditions as panel (**a**). Source data are provided as a Source Data file.

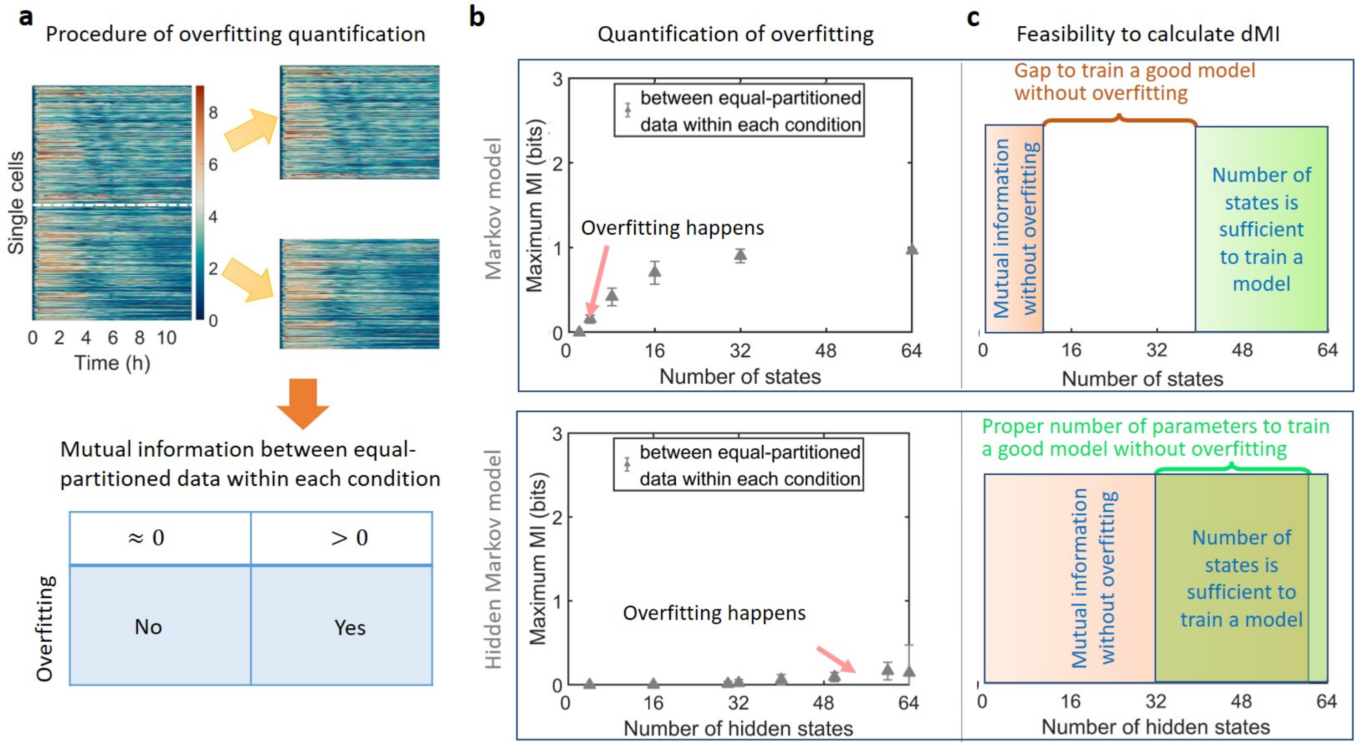

FIG. S5. (Color online) Quantification on overfitting and the feasibility to calculate dMI for the two models, as supplemental to Fig.2. (**a**) The schematic diagram for the procedure of quantifying overfitting by equally-partitioning data under each stimulus condition. Each trajectory ensemble is split equally into two subsets, and the mutual information between them is calculated, which gives a quantification on the overfitting of the model. (**b**) Averaged mutual information between equally-partitioned data within each stimulus versus the number of states. At each number of states, the average is taken on all the stimulus conditions, and the error bar denotes the standard deviation on all the 13 stimulus conditions. The time-inhomogeneous Markov model gives a pseudo nonzero mutual information between the two subsets of trajectory ensembles when the state number is just around 4 (pink arrow), whereas the hidden Markov model's maximal state number is around 60. (**c**) Schematic depicting the feasibility of the models. The number of states for good training performance comes from Fig.2. For the available data, the hidden Markov model has a valid range of hidden states around 30 to 60. Source data are provided as a Source Data file.

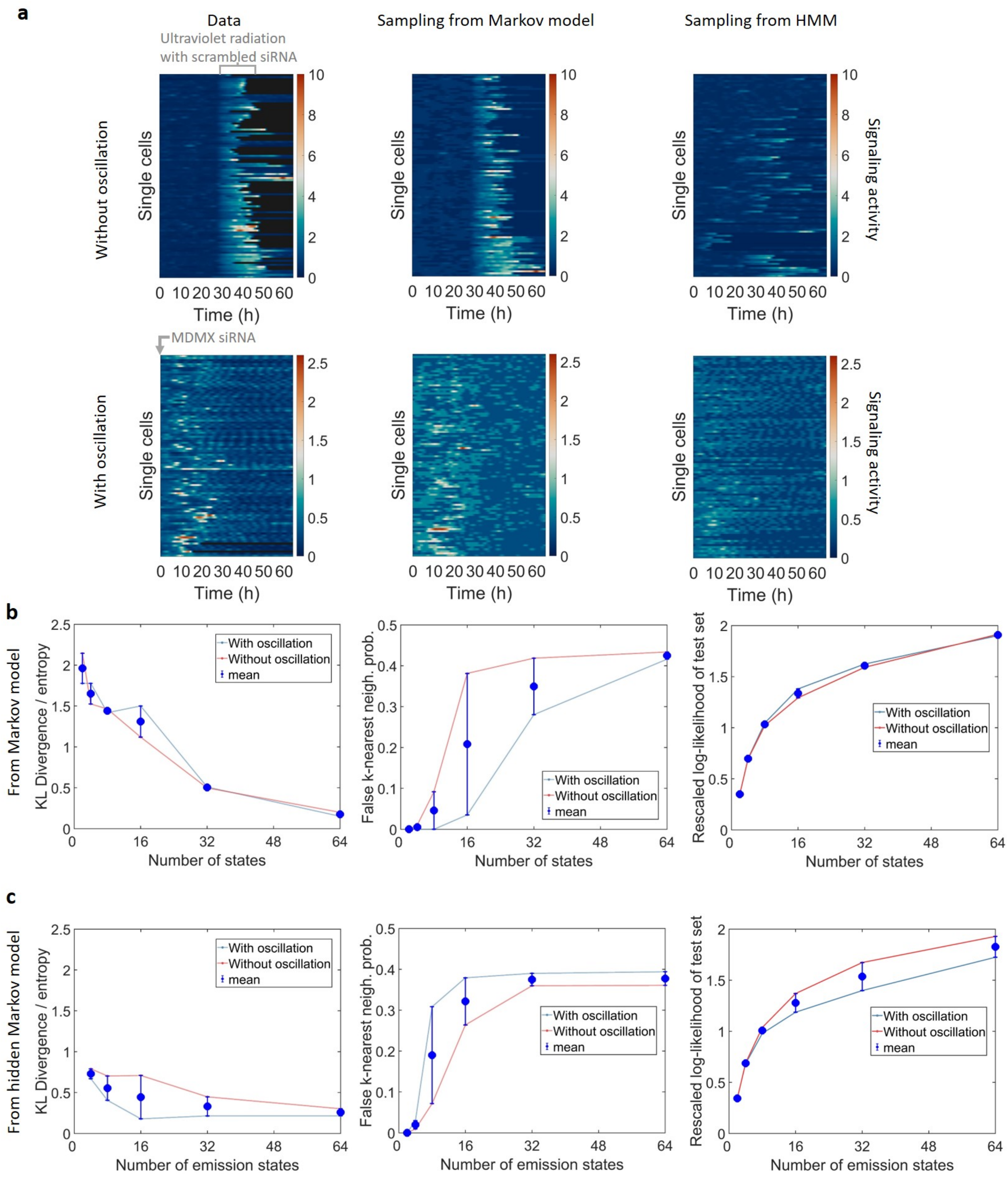

FIG. S6. (Color online) Learning the single-cell signaling dynamics of p53 by either a time-homogeneous Markov model or a hidden Markov model, as supplemental to Fig. 2. (**a**) Heatmaps of data and reproduced trajectories by the two models, as specified on the top. There are two conditions: the first row is dataset without oscillation, and the second is with oscillation. In the heatmap, there are around 100 rows, with each representing a single-cell response. There are 129 time points as the column. The data is uniformly normalized with maximum value 10, and the range of color is specified separately for each condition. (**b**) Quantification on the training performance for the time-homogeneous Markov model. We used the same three measures as Fig. 2. (**c**) Quantification on the training performance for the hidden Markov model. The numbers of hidden and emission states have a fixed ratio 2. In (**b**,**c**), the error bar denotes the standard deviation on the 2 conditions. From (**b**,**c**), the oscillatory condition is better modeled by hidden Markovian model, and the non-oscillatory condition is on the contrary. The data was shared by the authors of [38].

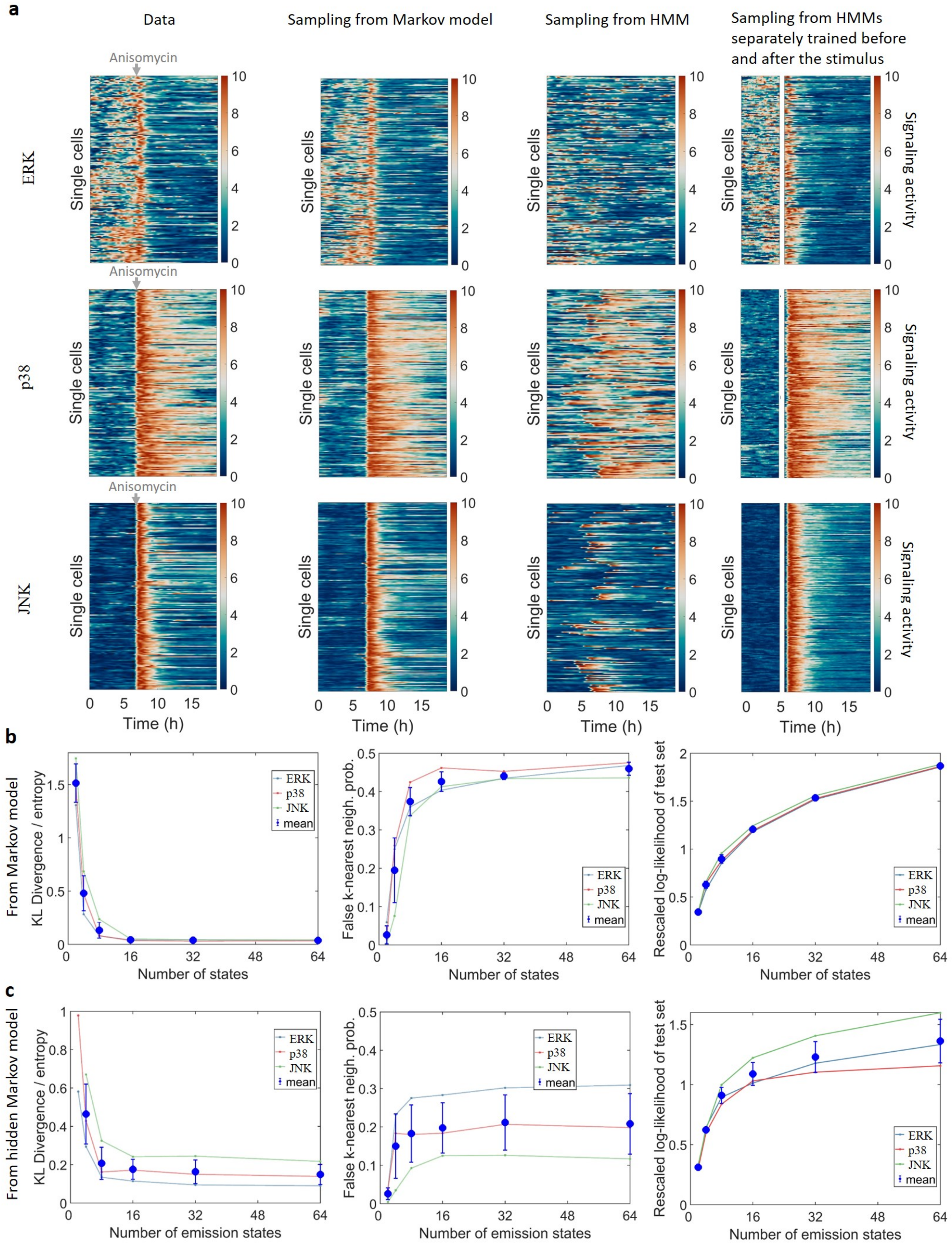

FIG. S7. (Color online) Learning the single-cell signaling dynamics of p38, JNK, ERK by either a time-homogeneous Markov model or a hidden Markov model, as supplemental to Fig. 2. (**a**) Heatmaps of data and reproduced trajectories by the two models, as specified on the top. The last column is reproduced by hidden Markov models separately trained for the two time windows before and after the stimulus, which generate more similar trajectories than that from the hidden Markov model trained by the full time window. There are 3 signaling molecules as denoted at the left. In the heatmaps, there are 200 single-cell responses as the row, and 150 time points as the column. The data are uniformly normalized with maximum value 10. (**b**,**c**) Quantification on the training performance for the two models trained by the full time window. The error bar denotes the standard deviation on the 3 stimulus conditions. This dataset is better modeled by the time-homogeneous Markov model, because the dynamical patterns are synchronized and do not have oscillation. The data was shared by the authors of [39].

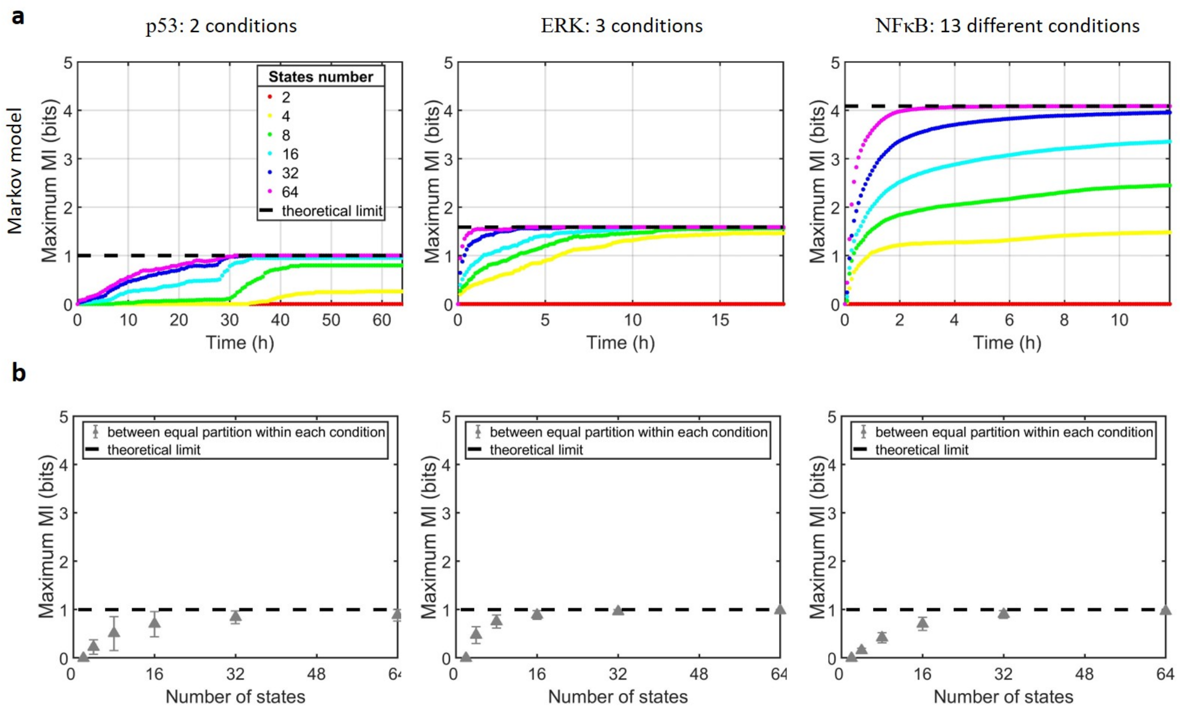

FIG. S8. (Color online)The maximum dMI estimated from the time-inhomogeneous Markov model shows overfitting of the model, as supplemental to Fig. 2. (**a**) The time course of maximum dMI for the three datasets: p53, ERK (p38, JNK), and NFκB. For each dataset, the number of conditions is listed at the top. The theoretical limit of maximum mutual information is $\log_2$ on the number of conditions. The number of states includes 2, 4, 8, 16, 32, 64, where the maximum mutual information keeps increasing with the number of states. (**b**) The maximum mutual information calculated between equal-partitioned data under each condition. It was repeated with using various number of states. Each dot is the average first on the whole time course of the dMI and second on all the conditions. The error bar denotes the standard deviation for all the conditions in each dataset: 2, 3, 13 conditions separately. The theoretical upper limit is $\log_2 2 = 1$, as the data is equally partitioned into two subsets. Since the two subsets are from the same condition, the ideal maximum mutual information should be 0 if there is no overfitting. However, they become nonzero when the number of state increases over 4, indicating a severe overfitting of the model. This figure shows that the mutual information from the Markov model tends to suffer from overfitting for available datasets. Note that the mutual information does not correspond to the stimulus discrimination for the p53 and ERK datasets, because they do not include multiple stimuli.

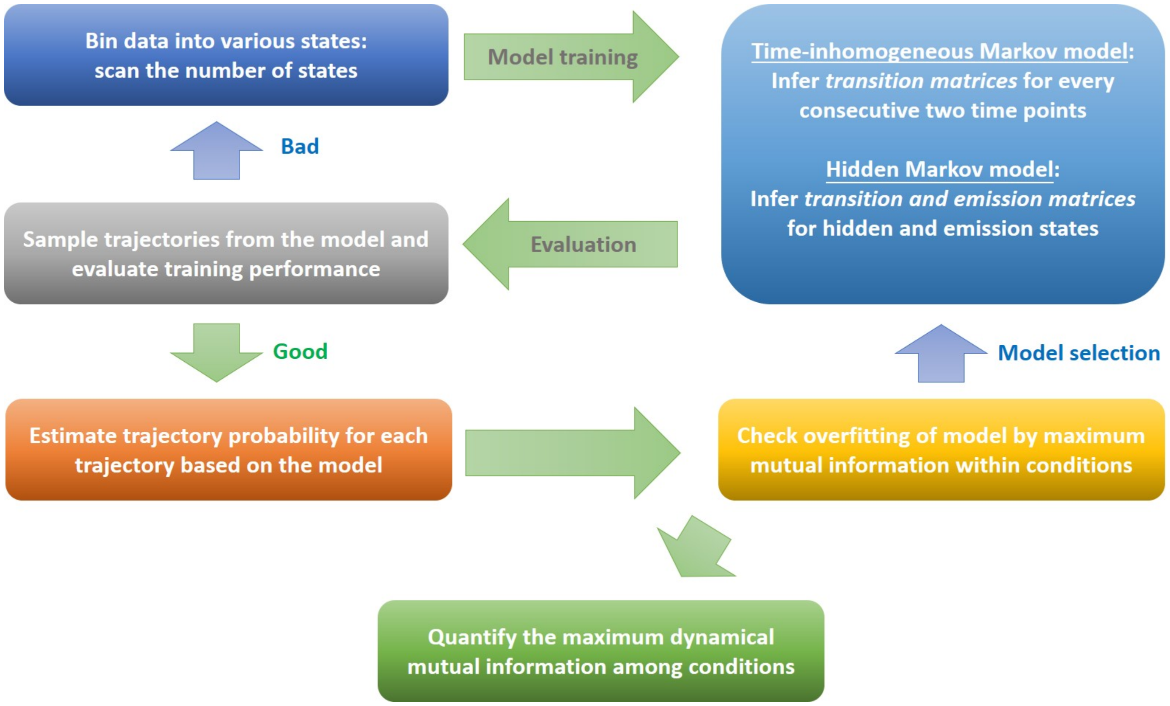

FIG. S9. (Color online) The workflow of calculating the maximum dMI, as supplemental to Fig. 3. It starts from binning the data with using a certain number of states. We then infer out the most probable transition matrices for the time-inhomogeneous Markov model, or transition matrix and emission matrix for the hidden Markov model. By using the performance measures, we either repeat the first step with increasing the number of states, or accept the number of states as a proper choice. Then, we estimate the maximum dMI of the two equal-partition sub-dataset within each condition, to quantify the overfitting of each model. This helps to choose the hidden Markov model with less overfitting. We then use the hidden Markov model to calculate trajectory probability, the dMI, and its maximization.

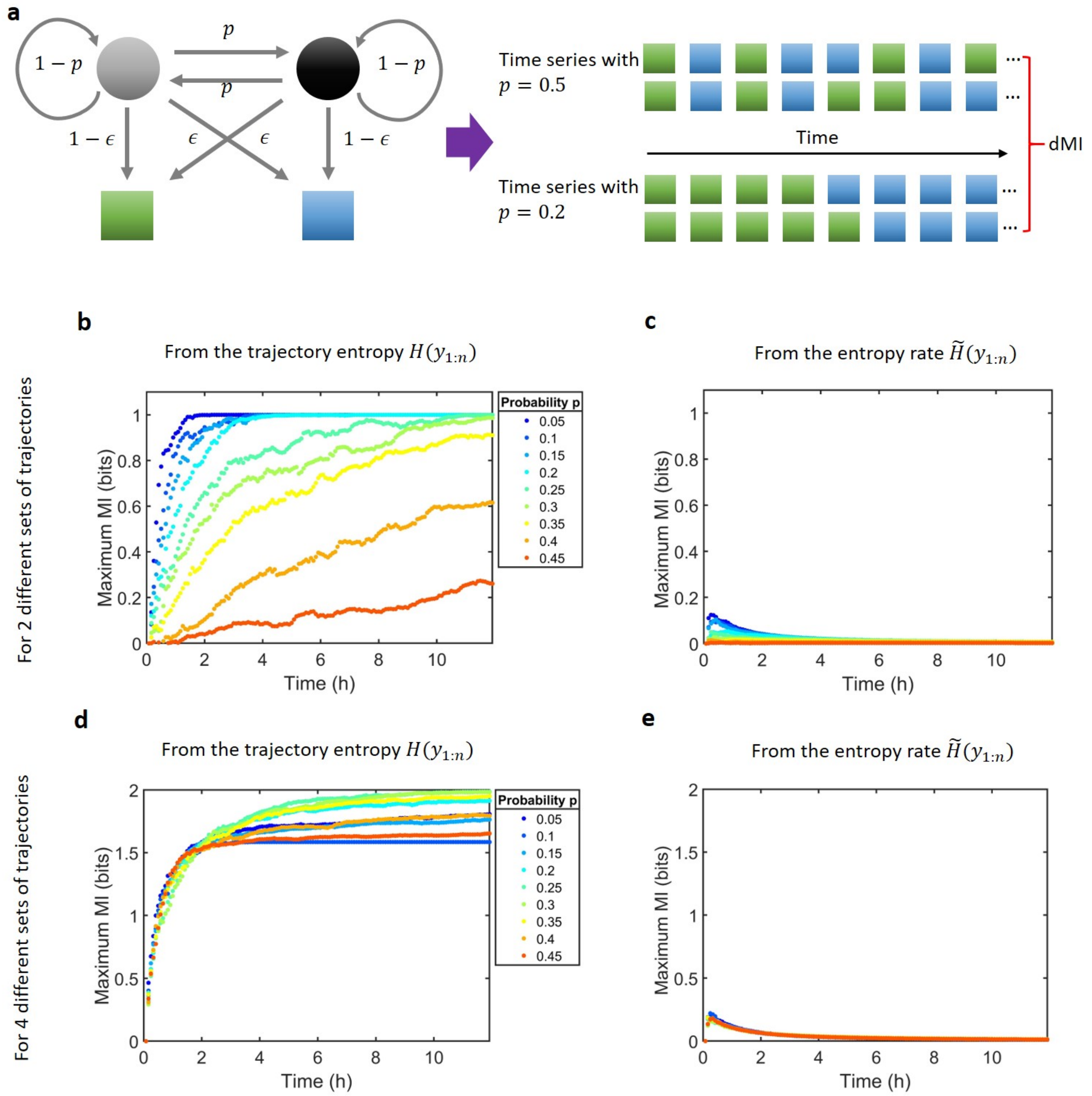

FIG. S10. (Color online) Validation on the formulation of the dMI in a toy model of the hidden Markov process, as supplemental to Fig. 3. (**a**) We considered a minimal model of the hidden Markov process, with two hidden states (circle) and emission states (square) shown in the model schematic. The hidden states can transit to each other with probability p, and emit to one state with probability ϵ. We used this model to generate two sets of time series, with each having a fixed p and ϵ. Two sets of the time series of the emission states were plotted, where we plotted two time series for each set. (**b**) The maximum dMI by Eqs. (1-3) with the trajectory entropy $H(y_{1:n})$ for the two sets of time series. The calculation was then repeated with one set of the time series having different p, denoted by the colors. They have distinct temporal profiles of the maximum dMI, depending on how distinguishable the two sets of time series are. (**c**) The dMI through using the entropy rate formula $\widetilde{H}(y_{1:n}) = -(1/n)\log_2 p(y_{1:n})$ with prefactor $1/n$ [14]. It gives the dMI converging to zero for the two distinguishable sets of time series, which is not proper. (**d**, **e**) The same figures as (**b**, **c**) for 4 different sets of trajectories, where the maximum mutual information is 2 bits under ideal transmission.

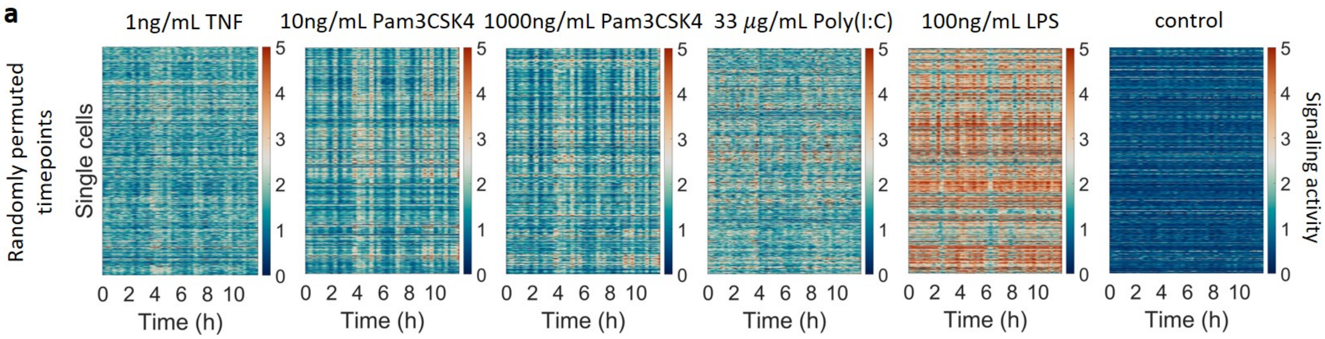

FIG. S11. (Color online) Examples of heatmaps for the data with randomly permuted timepoints, as supplemental to Fig. 3. (**a**) The concentration and type of stimuli are listed above. The colors illustrate the NFκB responses activity, and here the scale of color bar is consistent across heatmaps to better visualize the difference of response amplitudes. The trajectory ensembles have differences even after random permutation, leading to non-zero dMI estimates for the permuted data in Fig. 3. Source data are provided as a Source Data file.

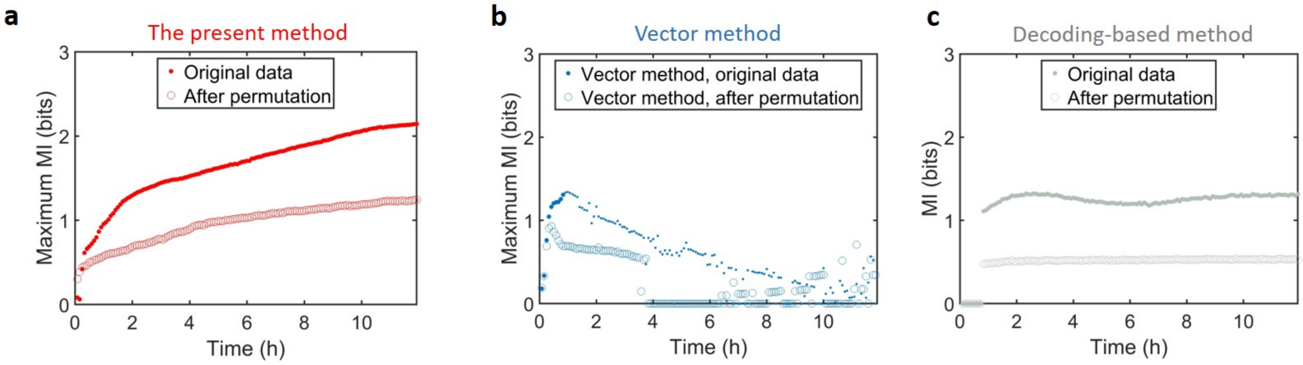

FIG. S12. (Color online) The mutual information for the data with randomly permuted time points. Here, every trajectory has the time points permuted with a random order separately, whereas the time points are permuted uniformly for all the trajectories in Fig. 3. The results for the non-permuted data are included for comparison. (**a**) The present method. (**b**) The vector method. It becomes inaccurate after around 10 time points. (**c**) The decoding-based method (with using the first 10 principal components). The information comes from that the trajectory ensembles have different peak amplitudes and integrals on average, which are preserved after the permutation. Source data are provided as a Source Data file.

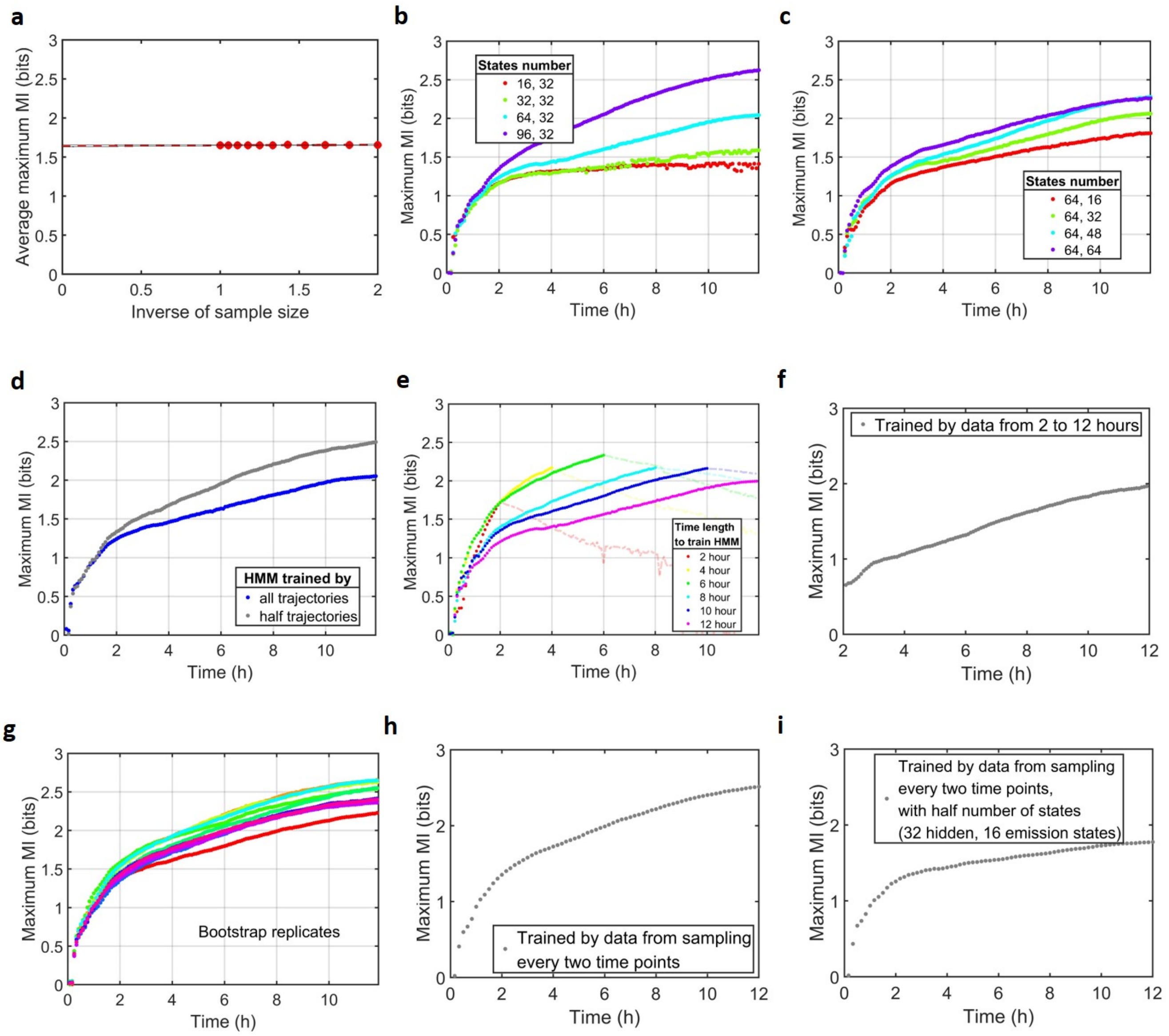

FIG. S13. (Color online) The effect of the parameters and the finite data sample on the dynamical mutual information from hidden Markov model, as supplemental to Fig. 3. (**a**) Jackknife method to estimate the effect of the subsample (without replacement) size of trajectories on the maximum dMI. Each value here is the averaged dMI along the whole time course. It is almost constant with respect to the inverse of the subsample size for estimating the trajectory probabilities. The dMI in other figures were normalized by the y-axis intersection here. (**b**) The maximum dynamical mutual information varies with the number of hidden states, when the number of emission states is fixed. In the legend, the first number is for hidden states and the second for emission states. (**c**) The dynamical mutual information depends on the number of emission states, when the number of hidden states is fixed. (**d**) The dynamical mutual information when the model was trained with using different number of trajectories in each condition. If only using half trajectories to train the model, the maximum mutual information becomes higher, which may be caused by the overfitting issue. (**e**) The dynamical mutual information when the model was trained with different time course of trajectories in each condition: 2, 4, 6, 8, 10, 12 hours. For each time length, the maximum mutual information beyond the training time window decays. (**f**) The dMI for the time window from 2 hours to 12 hours, where the model is trained by the data in this window. This dMI in the late time window is slightly overestimated due to the overfitting when less timepoints are used. (**g**) The dMI for the bootstrap replicates of data. Each replicate has the same amount of data by randomly sampling from the original dataset with replacement. We repeated the whole process of model training and dMI estimation for the replicates. (**h**) The dMI for the data with subsampled timepoints, where every two time points were used. The dMI has qualitatively similar behaviors as Fig. 3, but is slightly overestimated due to the overfitting when fewer timepoints are used. (**i**) The dMI for the data with subsampled timepoints, by using half the number of states (32 hidden and 16 emission states). Source data are provided as a Source Data file.

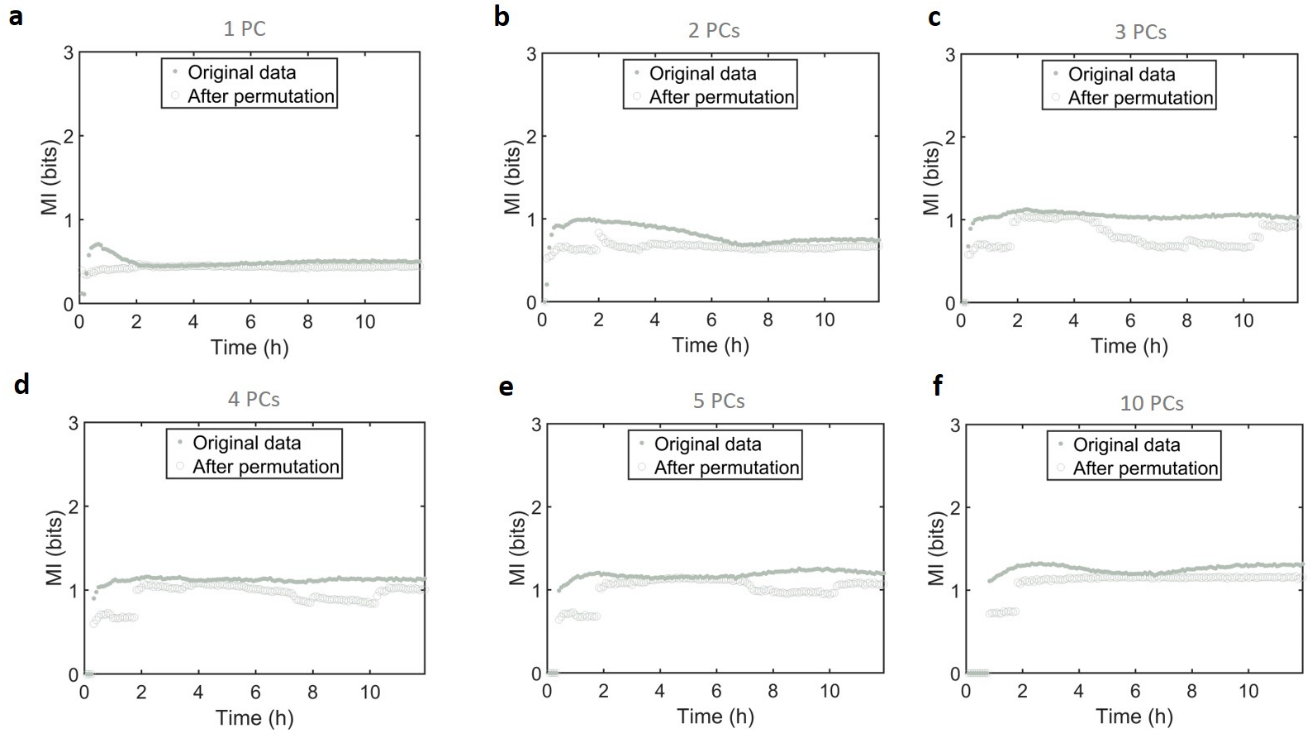

FIG. S14. (Color online) The lower bound of mutual information from the decoding-based method with using different number of principle components (PCs). It is conducted for the same stimulus conditions in Fig. 3. (**a**-**f**) Results with using the first 1, 2, 3, 4, 5, 10 PCs. The mutual information reaches maximum with around the first 5 PCs. Under sufficient number of PCs, the mutual information has nearly a jump from zero to the saturated value at certain time point depending on the number of PCs, and keeps almost constant afterwards. The permutation of time points leads to similar saturated values. Source data are provided as a Source Data file.

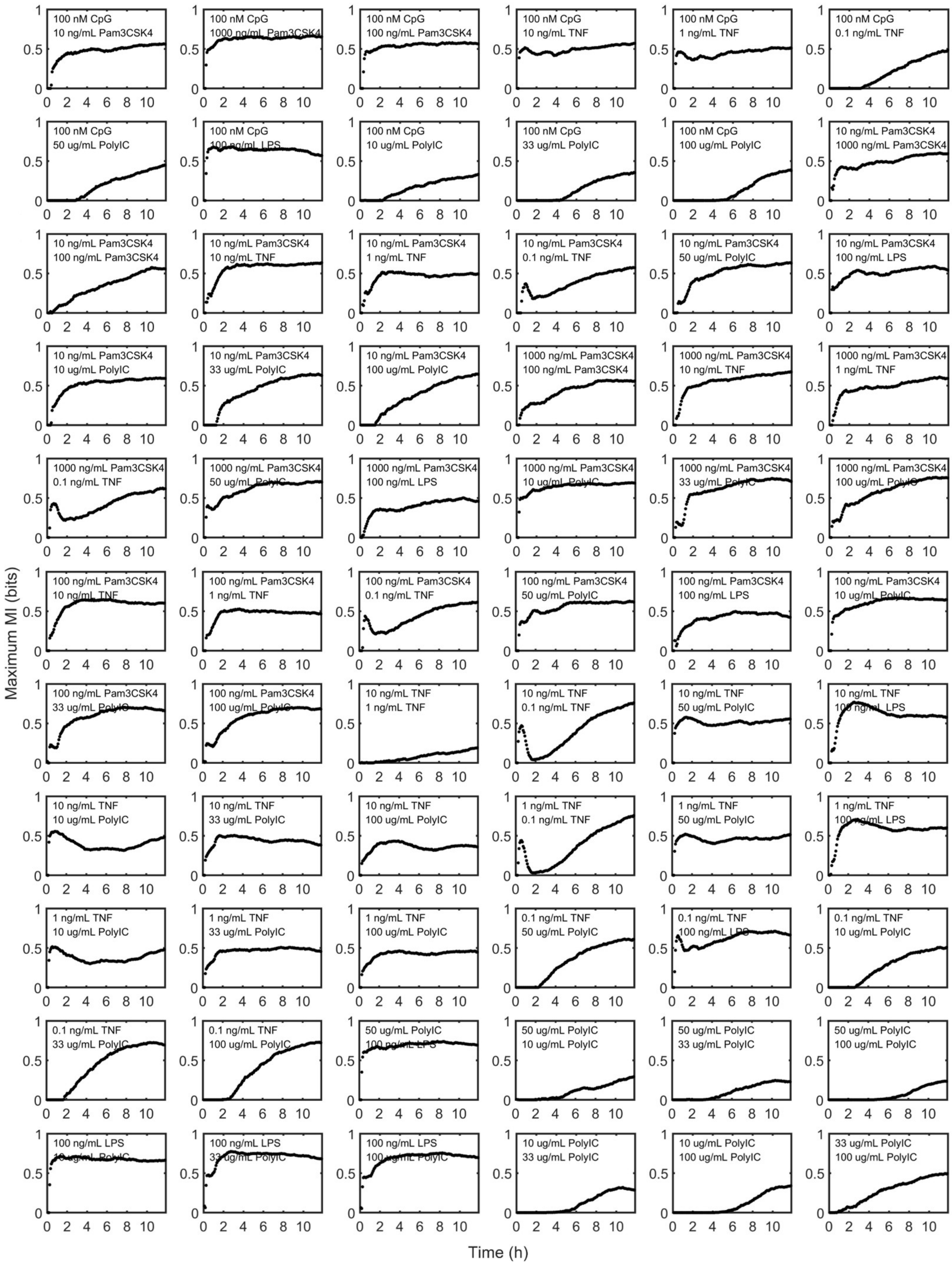

FIG. S15. (Color online) The dMI for examples of pairwise stimuli in the NFκB dataset, as supplemental to Fig. 4. They have distinct temporal profiles of the information accumulation. Source data are provided as a Source Data file.

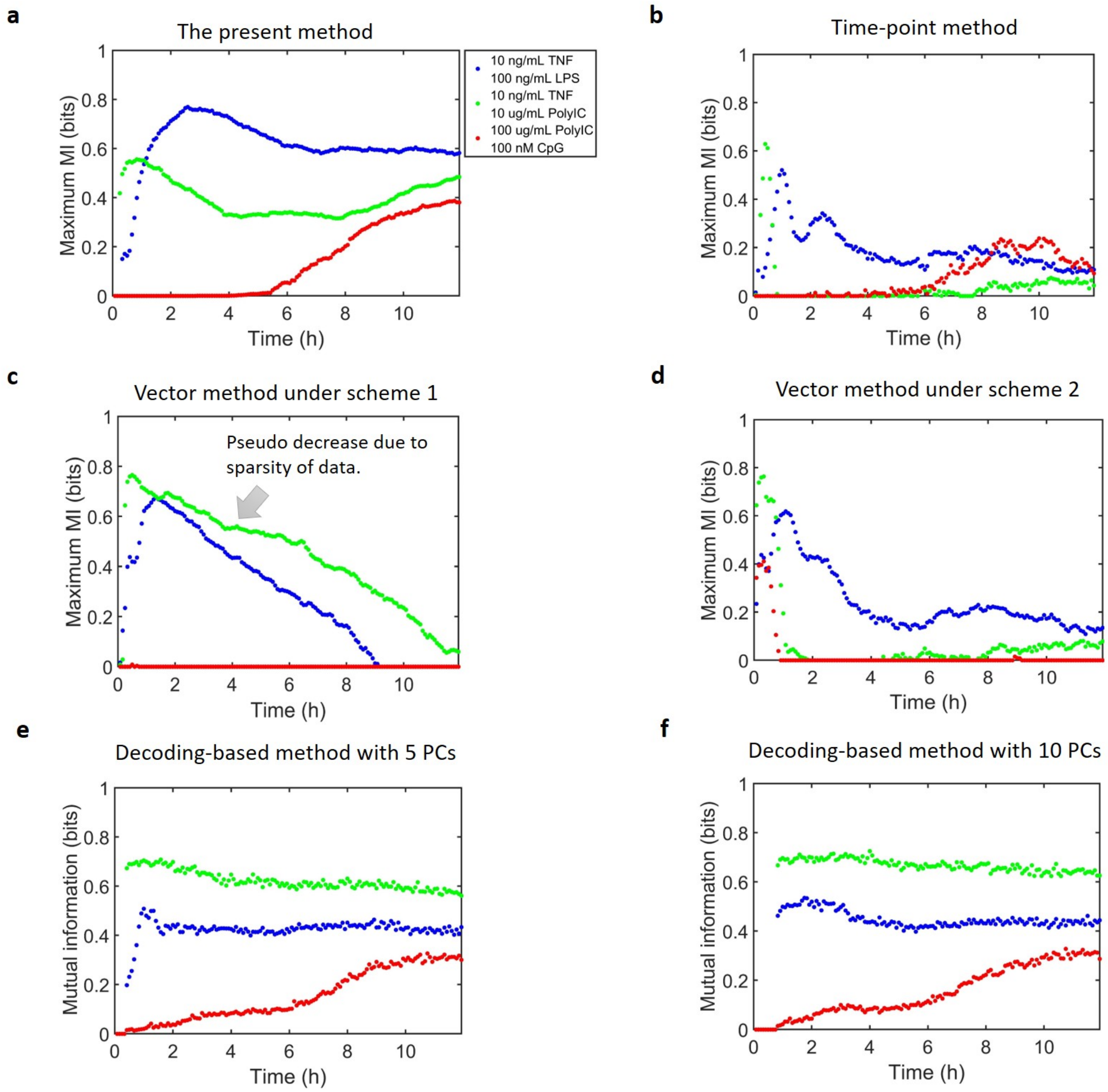

FIG. S16. (Color online) The mutual information for the pairwise stimuli of Fig. 4 by using various methods. The colors specify the same pairwise stimuli for all the panels, and are the same as Fig. 4. (**a**) The same result of Fig. 4 from the present method, included for comparison. (**b**) The time-point method, which does not fully reveal the information accumulation. For example, the pairwise stimuli TNF and LPS have decaying mutual information to less than 0.2 bit after 3 hours, but the dynamical patterns are still largely distinct in Fig. 4**b**. (**c**) The vector method under scheme 1 (with using data of successive time points), with a pseudo decaying mutual information over around 10 time points. The temporal order of distinguishing various pairwise stimuli is not shown by the vector method. (**d**) The vector method under scheme 2 (with using 5 neighbor data points at each time point). Since the vector method becomes inaccurate over 10 time points, we have employed the scheme 2, which still generates mainly decreasing mutual information after 1 hour. (**e**,**f**) The decoding-based method with using the first 5 PCs and 10 PCs. The mutual information for TNF-LPS pair is overall lower than that for TNF-Poly(I:C) pair, which does not match with the temporal similarity of trajectory ensembles with TNF-LPS pair more distinguishable (Fig. 4**b**). Since the method used the linear principle components for the classifier without employing the dynamical features coherently, it is less accurate to extract the information from the oscillatory pattern. Source data are provided as a Source Data file.

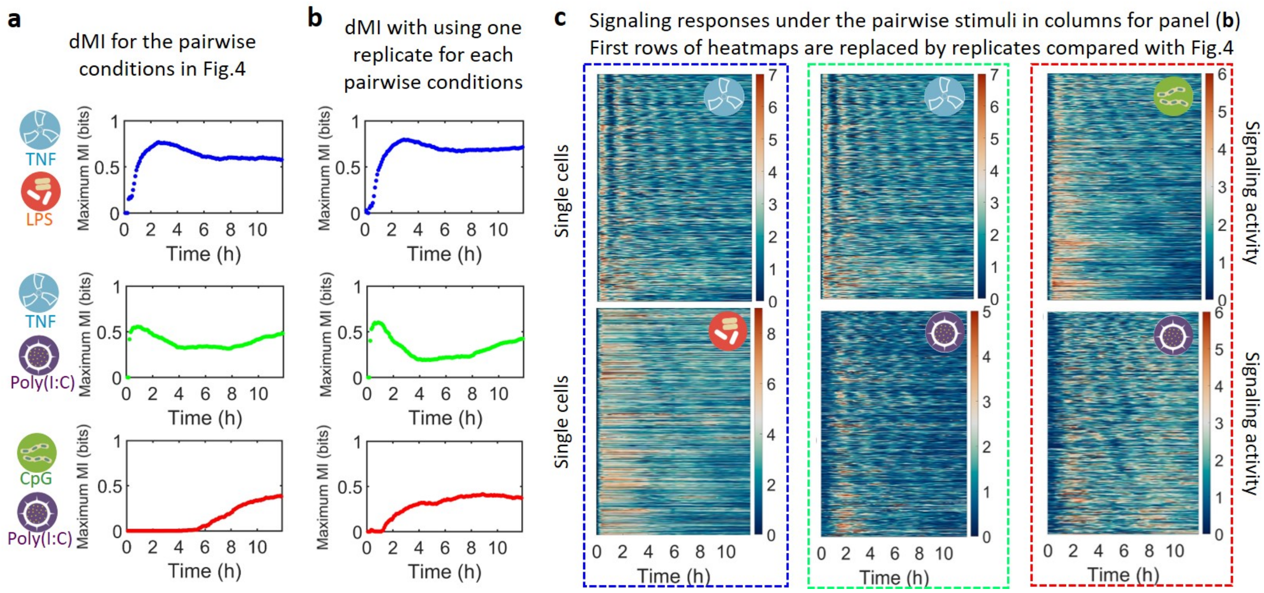

FIG. S17. (Color online) The dMI for the pairwise stimulus conditions using replicates, as supplemental to Fig. 4. (**a**) The maximum dMI for the 3 pairs of stimuli as in Fig. 4: 10 ng/ml TNF and 100 ng/ml LPS (blue); 10 ng/ml TNF and 10 μg/ml Poly(I:C) (green); 100 nM CpG and 100 ng/ml Poly(I:C) (red). (**b**) The maximum dMI for the 3 pairs of stimuli using replicates. The dMI are similar to (**a**), validating the robustness of dMI calculations. For each pairwise comparison, one stimulus condition is replaced by the replicate data: 10 ng/ml TNF for the blue; 10 ng/ml TNF for the green; 100 nM CpG for the red. The replicates were generated at different dates (Table S3), serving as the out-of-sample validation. (**c**) The heatmaps of the NFκB response for the corresponding stimuli pairs of (**b**) grouped in columns. The time regime with relatively distinct dynamical patterns leads to the high maximum dMI in (**b**). Note that the two dMI in red differ in the onset time, which can be attributed to technical variations in the measured data between the replicates (the upper heatmap in the red box of Fig. 4 and this figure). The dMI robustly reveals such difference. Source data are provided as a Source Data file.

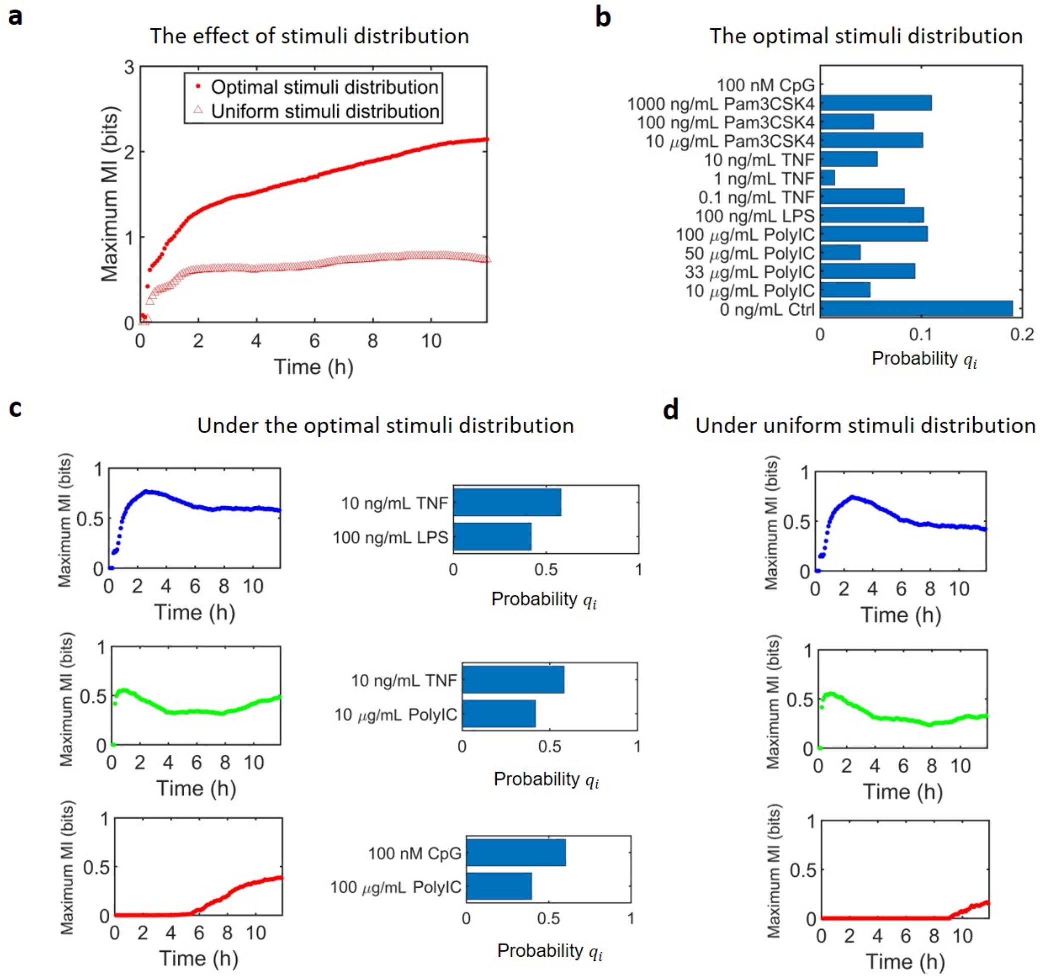

FIG. S18. (Color online) The dMI using different stimuli distributions as supplemental to Fig. 3 and Fig. 4. (**a**) The dMI under the optimized distribution of the 13 stimulus conditions as in Fig. 3 and under the uniform distribution. (**b**) The optimal stimuli distribution obtained by maximizing dMI. (**c**) The dMI under the optimized distribution of the pairwise stimulus conditions as in Fig. 4 and the corresponding optimal stimuli distribution. (**d**) The dMI under the uniform distribution of the pairwise stimulus conditions. Source data are provided as a Source Data file.

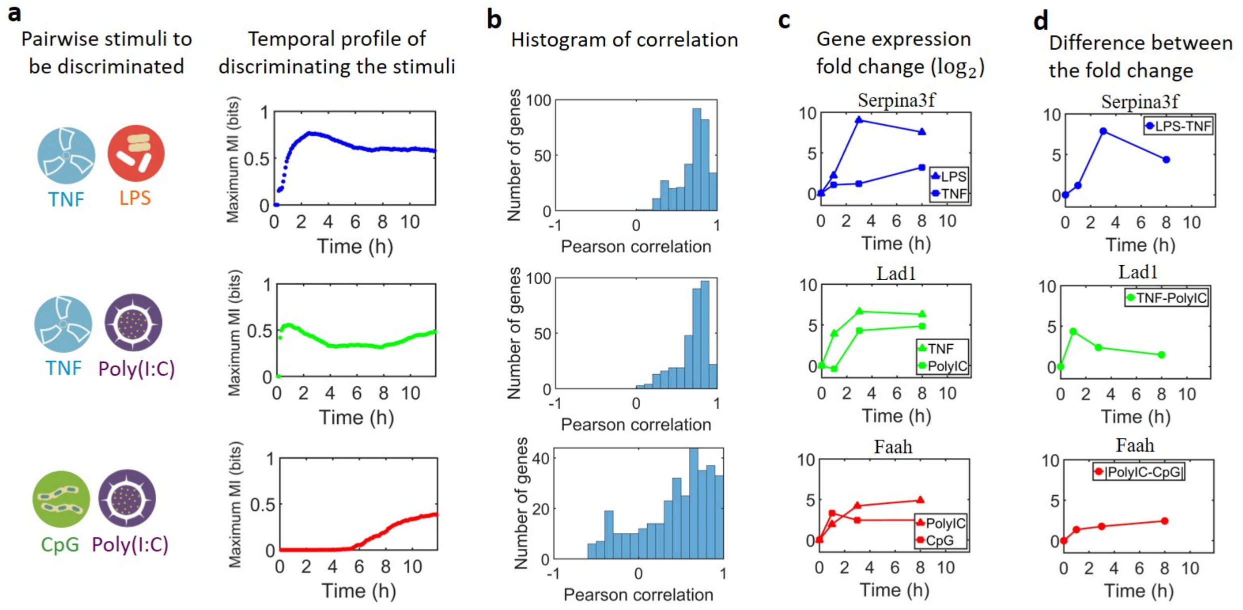

FIG. S19. (Color online) The information for stimulus discrimination may be decoded by responsive genes, as supplemental to Fig. 4. (**a**) The same figures of maximum dMI for 3 stimuli pairs as in Fig. 4: 10 ng/ml TNF and 100 ng/ml LPS (blue); 10 ng/ml TNF and 10 μg/ml Poly(I:C) (green); 100 nM CpG and 100 ng/ml Poly(I:C) (red). (**b**) The correlation between the dMI values and the absolute difference of gene expression fold change between the pairwise stimuli. Gene expression were measured by Illumina bead array at three time points (1, 3, 8 hours) under the same stimulus conditions in the same cell type [27]. Expression data from NFκB-response genes (cluster B,C in Fig. 5 of [27]) were used. (**c**) Three representative NFκB-responsive genes under the indicated stimulus, which potentially use the information of NFκB signaling dynamics to produce stimulus-specific gene expression. The gene expression fold change is in log scale. (**d**) The absolute difference in gene expression, corresponding to the information accumulation in (**a**).

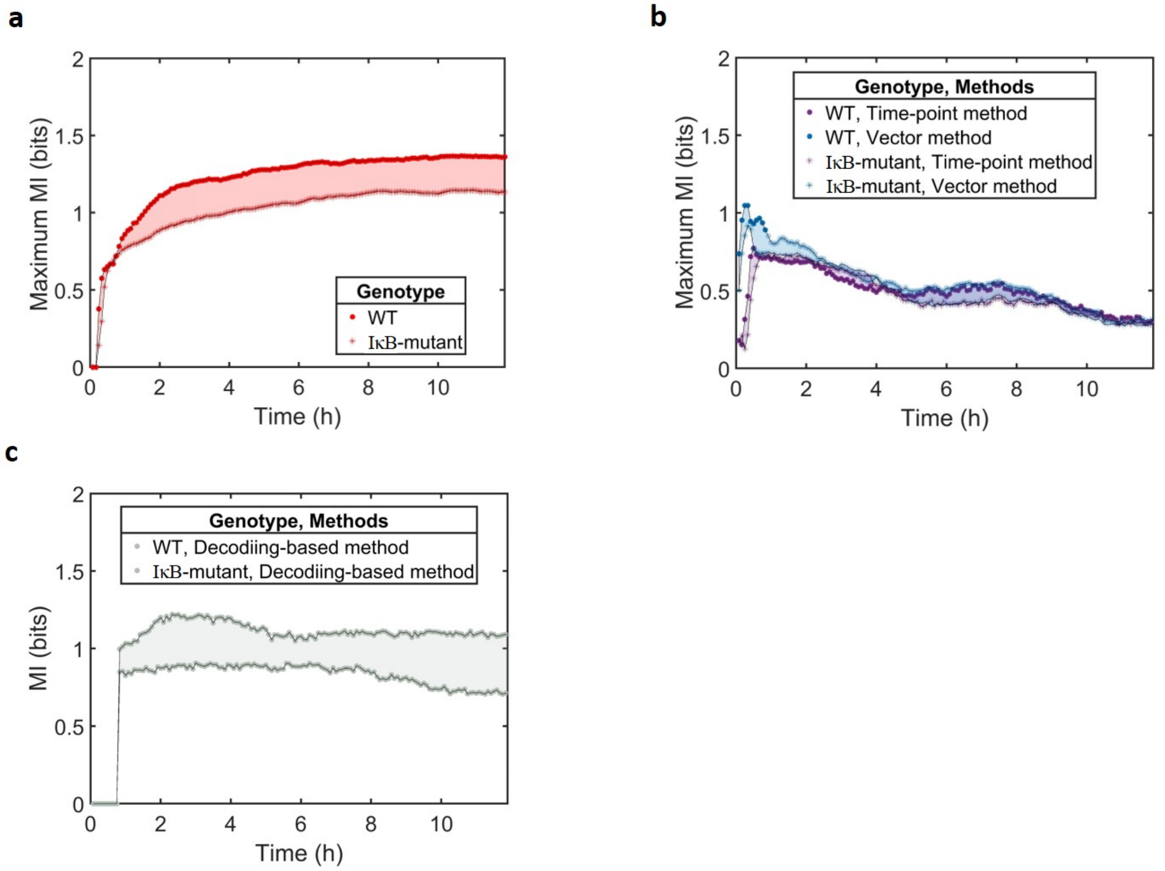

FIG. S20. (Color online) The mutual information from various methods for the two genotypes, as supplemental to Fig. 5. (**a**) Fig. 5's panel (**b**), included for comparison. (**b**) The maximum mutual information among the same stimulus conditions for WT and IκB-mutant, calculated from the time-point and vector methods. For the vector method, we used scheme 2 (5 neighbor data points at each time point), as using data of successive time points leads to pseudo decaying mutual information when the dimension increases over around 10. (**c**) The mutual information from the decoding-based method with 10 PCs. The mutual information has a sudden change at the 10 time point for both genotypes. Source data are provided as a Source Data file.

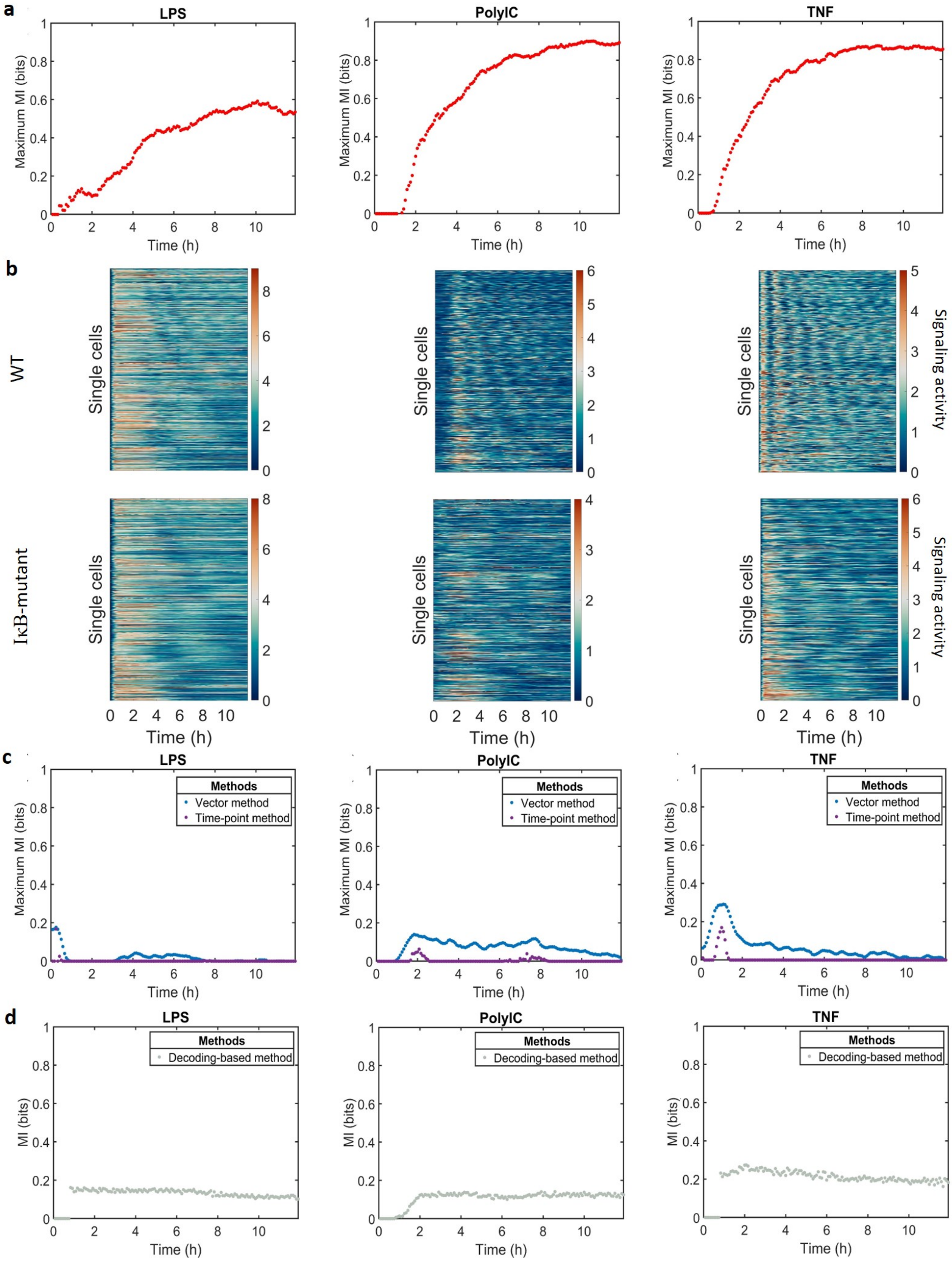

FIG. S21. (Color online) The maximum mutual information between the two genotypes, WT and IκB-mutant, under the same condition separately. It is supplemental to Fig. 5. The conditions are 100 ng/mL LPS, 50 μg/mL Poly(I:C), 10 ng/mL TNF. (**a**) The maximum dMI between the two genotypes from the present method. (**b**) The heatmaps of the single-cell NFκB signaling trajectories for the two genotypes. The pairwise trajectory ensembles between the genotypes are visually distinguishable, which matches with the dMI in (**a**). (**c**) The maximum mutual information between the two genotypes, from the time-point method and the vector method under scheme 2 (with using 5 neighbor data points at each time point). (**d**) The decoding-based method with using 10 PCs. The value of information from the previous three methods may be underestimated, and is ~ 0.2 bit for the largely distinguishable two trajectory ensembles, such as the case of TNF. Source data are provided as a Source Data file.

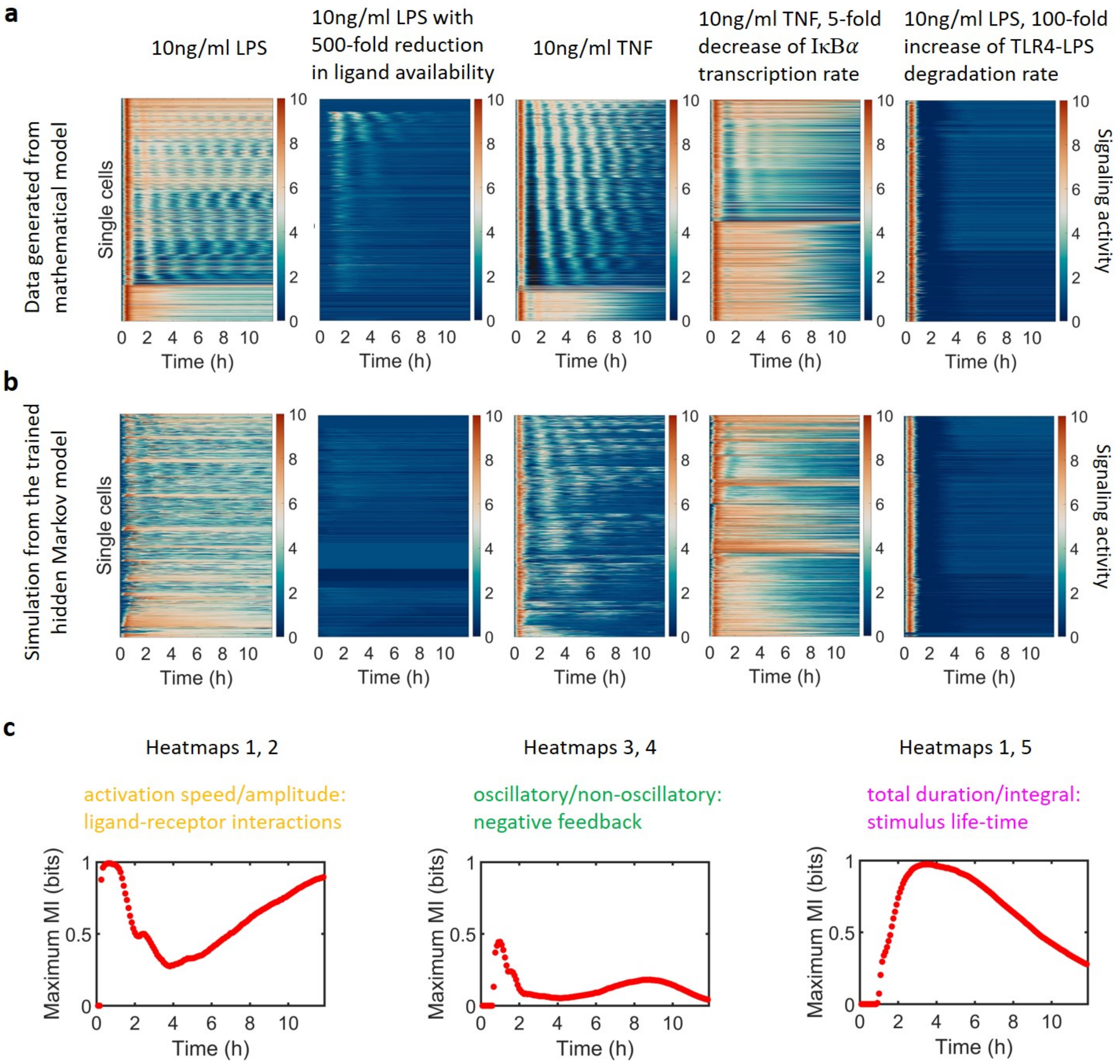

FIG. S22. (Color online) Exploring the temporal phases of dMI by using a mathematical model of NFκB signaling network, as supplemental to Fig. 5. (**a**) The data generated from a mathematical model of the NFκB signaling network [32] with distributed parameters as in [33, 34]. The heatmaps of the simulated NFκB responses have the distinct features shown in Fig. 5. The concentration and type of stimuli and the perturbation on the parameters are listed above. The colors of heatmaps illustrate the NFκB responses activity normalized to 10. (**b**) The heatmaps of the sampled trajectories from the hidden Markov model. (**c**) The dMI for the chosen pairwise conditions as listed on top. Under the pairwise conditions, the signaling responses have distinct features caused by the specific perturbation on the signaling network, as listed in the middle row. Correspondingly, the first major gain of dMI is from three temporal phases as speculated in Fig. 5: early-phase increase; intermediate-phase gain at about 1 hour; relatively-late increase at about hours.

VII. SUPPLEMENTARY TABLES

TABLE S1. A list of the mathematical symbols used in this manuscript.

Symbols	Quantities
N	Total number of time points
n	A specific number of time point
x, y	Time series of states
M	Total number of conditions
i	Index of condition
k	Index of condition, index of hidden states
m_i	Total number of trajectories for the i-th stimulus
j	Index of trajectories under each stimulus
E	Matrix of emission probability
T	Matrix of transition probability between hidden states
$\hat{T}$	Matrix of transition probability for filtered probability
B	The number of emission states
K	The number of hidden states
l,$l^{'}$	Index of the states
R	The signaling responses
S	The stimuli
$\mathbf{q}$	Probability distribution of the stimulus conditions
p, P	Various probabilities depending on the postfix
$M1$, $M1$	Index of conditions for the toy model
n_1, n_2	The number of states flips for the toy model

TABLE S2. A list of the symbols separately for the trajectory-wise and model-wise quantities.

Symbols	Quantities
H	Trajectory-wise trajectory entropy for subsamples
I	Trajectory-wise dynamical mutual information
I_{max}	Trajectory-wise maximum dynamical mutual information
$\tilde{H}$	Previous trajectory-wise trajectory entropy (entropy rate)
$\hat{H}$	Model-wise trajectory entropy with the probability as prefactor
$\hat{I}$	Model-wise mutual information
$\hat{I}_{max}$	Model-wise maximum mutual information

TABLE S3. List of experimental conditions, the number of single-cell response trajectories, the number of time points and the experimental date for the NFκB dataset.

ID	Condition	Cell counts	Time frames	Experimental date
157	100 nM CpG	501	143	11/6/2015
546	10 ng/mL Pam3CSK4	499	143	10/18/2016
548	1000 ng/mL Pam3CSK4	554	143	10/18/2016
581	100 ng/mL Pam3CSK4	641	143	2/7/2017
610	100 nM CpG	729	143	4/1/2017
650	10 ng/mL TNF	1249	143	5/29/2017
663	1 ng/mL TNF	847	143	6/1/2017
664	0.1 ng/mL TNF	524	143	6/1/2017
720	100 ng/mL Pam3CSK4	699	143	9/28/2017
754	10 ng/mL TNF	587	143	10/26/2017
755	50 μg/mL Poly(I:C)	634	143	10/26/2017
756	100 ng/mL LPS	731	143	10/26/2017
777	10 μg/mL Poly(I:C)	725	143	12/22/2017
778	33 μg/mL Poly(I:C)	585	143	12/22/2017
779	100 μg/mL Poly(I:C)	591	143	12/22/2017
780	33 μg/mL Poly(I:C)	690	143	12/22/2017
783	Control	623	143	1/18/2018
759	IκB$\alpha^{M/M}$: 100 ng/mL LPS	934	143	10/27/2017
760	IκB$\alpha^{M/M}$: 50 μg/mL Poly(I:C)	614	143	10/27/2017
761	IκB$\alpha^{M/M}$: 10 ng/mL TNF	524	143	10/27/2017

TABLE S4. List of number of single-cell response trajectories and time points for the p53 dataset [38].

Condition	Cell counts	Time frames
Without oscillation	100	129
With oscillation	101	129

TABLE S5. List of number of single-cell response trajectories and time points for the p38, JNK, ERK dataset [39].

Molecule	Cell counts	Time frames
ERK	200	150
p38	200	150
JNK	200	150
