# Supplementary material for: Quantifying information accumulation encoded in the dynamics of biochemical signaling
Source: Nat Commun. 2021 Feb 24;12:1272. doi: 10.1038/s41467-021-21562-0 (PMC7904837; doi:10.1038/s41467-021-21562-0)
Supplement: Supplementary file 3 — Reporting Summary [file 41467_2021_21562_MOESM3_ESM.pdf]

nature research

Corresponding author(s): Alexander Hoffmann

Last updated by author(s): Jan 15, 2021

Reporting Summary

Nature Research wishes to improve the reproducibility of the work that we publish. This form provides structure for consistency and transparency in reporting. For further information on Nature Research policies, see our Editorial Policies and the Editorial Policy Checklist.

Statistics

For all statistical analyses, confirm that the following items are present in the figure legend, table legend, main text, or Methods section.

n/a	Confirmed	
☐	☒	The exact sample size (n) for each experimental group/condition, given as a discrete number and unit of measurement
☐	☒	A statement on whether measurements were taken from distinct samples or whether the same sample was measured repeatedly
☐	☒	The statistical test(s) used AND whether they are one- or two-sided<br>*Only common tests should be described solely by name; describe more complex techniques in the Methods section.*
☒	☐	A description of all covariates tested
☐	☒	A description of any assumptions or corrections, such as tests of normality and adjustment for multiple comparisons
☐	☒	A full description of the statistical parameters including central tendency (e.g. means) or other basic estimates (e.g. regression coefficient) AND variation (e.g. standard deviation) or associated estimates of uncertainty (e.g. confidence intervals)
☐	☒	For null hypothesis testing, the test statistic (e.g. F, t, r) with confidence intervals, effect sizes, degrees of freedom and P value noted<br>*Give P values as exact values whenever suitable.*
☐	☒	For Bayesian analysis, information on the choice of priors and Markov chain Monte Carlo settings
☒	☐	For hierarchical and complex designs, identification of the appropriate level for tests and full reporting of outcomes
☒	☐	Estimates of effect sizes (e.g. Cohen's d, Pearson's r), indicating how they were calculated

Our web collection on statistics for biologists contains articles on many of the points above.

Software and code

Policy information about availability of computer code

Data collection	We used an automated image analysis workflow from the Github open-source code: https://github.com/Adewunmi91/MACKtrack .
Data analysis	We developed the MATLAB code package dMI for data analysis, which is available at GitHub: https://github.com/signalingsystemslab/dMI with a guideline on the website: https://sites.google.com/view/dmipackage .<br>For the decoding-based method, we used the Github open-source code: https://github.com/swainlab/mi-by-decoding .<br>The Baum-Welch algorithm was implemented by the MATLAB build-in function "hmmtrain": https://www.mathworks.com/help/stats/hmmtrain.html . The hidden Markov model was trained by the MATLAB build-in package: https://www.mathworks.com/help/stats/hidden-markov-models-hmm.html . The MATLAB build-in packages have the version R2018b.<br>All the external packages above have a single version.<br>All the simulations were done with MATLAB version R2018b

For manuscripts utilizing custom algorithms or software that are central to the research but not yet described in published literature, software must be made available to editors and reviewers. We strongly encourage code deposition in a community repository (e.g. GitHub). See the Nature Research guidelines for submitting code & software for further information.

Data

Policy information about availability of data

All manuscripts must include a data availability statement. This statement should provide the following information, where applicable:

- Accession codes, unique identifiers, or web links for publicly available datasets
- A list of figures that have associated raw data
- A description of any restrictions on data availability

The authors declare that the data supporting the findings of this study are available within the paper [and its supplementary information files].

Field-specific reporting

Please select the one below that is the best fit for your research. If you are not sure, read the appropriate sections before making your selection.

☒ Life sciences ☐ Behavioural & social sciences ☐ Ecological, evolutionary & environmental sciences

For a reference copy of the document with all sections, see nature.com/documents/nr-reporting-summary-flat.pdf

Life sciences study design

All studies must disclose on these points even when the disclosure is negative.

Sample size	There are 13 different stimulus conditions (samples). The 13 conditions come from 5 different ligands, including cytokine TNF and viral and bacterial PAMPs. Ligand concentrations were based on bioRxiv 2020.05.23.112862 (2020), and were chosen to induce a range of dynamical patterns of NFkB responses.
Data exclusions	No data were excluded.
Replication	Four replicate datasets for WT cells were generated over a period of 24 months for comparing an identical condition. Mutual information estimates were the consistent for all replicates. The date of the experiments are provided in Table.S3.
Randomization	We take all the samples together for the analysis without randomization, because we chose the 13 stimulus conditions to generate representative NFkB signaling responses and use all of them to study the stimulus discrimination. When analyzing the data, we have randomly sampled the signaling trajectories from the data under each stimulus conditions, and conducted a bootstrap procedure of the data to calculate the mutual information. The result in Fig.S13g shows that our result is robust with the random sampling on the signaling trajectories.
Blinding	Blinding was not relevant to our study.

Reporting for specific materials, systems and methods

We require information from authors about some types of materials, experimental systems and methods used in many studies. Here, indicate whether each material, system or method listed is relevant to your study. If you are not sure if a list item applies to your research, read the appropriate section before selecting a response.

Materials & experimental systems

n/a	Involved in the study	
☒	☐	Antibodies
☐	☒	Eukaryotic cell lines
☒	☐	Palaeontology and archaeology
☐	☒	Animals and other organisms
☒	☐	Human research participants
☒	☐	Clinical data
☒	☐	Dual use research of concern

Methods

n/a	Involved in the study	
☒	☐	ChIP-seq
☒	☐	Flow cytometry
☒	☐	MRI-based neuroimaging

Eukaryotic cell lines

Policy information about cell lines

Cell line source(s)	No cell lines were used, but primary cells, namely Bone Marrow-Derived Macrophages (BMDMs) were prepared by culturing bone marrow cells prepared from femurs of 8-24 weeks old mice of either sex (see below) in L929-conditioned medium.
Authentication	No cell lines were used.
Mycoplasma contamination	Cell cultures were not tested for mycoplasma contamination.
Commonly misidentified lines (See ICLAC register)	n/a.

Animals and other organisms

Policy information about studies involving animals; ARRIVE guidelines recommended for reporting animal research

Laboratory animals	The mVenus-RelA mouse line (C57BL/6J background) was generated by Ingenious Targeting Laboratory. For some experiments it was crossed with the IκBα κB/+ line (also C57BL/6J background; kind gift from Paul Chiao) that harbors mutated κB sites in the IκBα promoter, to generate double homozygous mutants. All mice strains have either sex, and are 8-24 weeks old. Mice were housed in

	an accredited UCLA Vivarium at ambient temperature and humidity with a 7am-7pm light/dark cycle.
Wild animals	The study did not involve wild animals.
Field-collected samples	The study did not involve samples collected from the field.
Ethics oversight	The UCLA Institutional Animal Care and Use Committee (IACUC) approved the protocol for animal research per guidance form the American Veterinary Medical Association.

Note that full information on the approval of the study protocol must also be provided in the manuscript.
